# Host transcriptomic profiling of CD-1 outbred mice with severe clinical outcomes following infection with *Orientia tsutsugamushi*

**DOI:** 10.1371/journal.pntd.0010459

**Published:** 2022-11-23

**Authors:** Joseph Thiriot, Yuejin Liang, James Fisher, David H. Walker, Lynn Soong

**Affiliations:** 1 Department of Microbiology and Immunology, University of Texas Medical Branch, Galveston, Texas, United States of America; 2 Institute for Human Infection and Immunity, University of Texas Medical Branch, Galveston, Texas, United States of America; 3 Department of Pathology, University of Texas Medical Branch, Galveston, Texas, United States of America; University of Peradeniya Faculty of Medicine, SRI LANKA

## Abstract

*Orientia tsutsugamushi* is an obligately intracellular bacterium with endothelial tropism and can cause mild to lethal scrub typhus in humans. No vaccine is available for this reemerging and severely neglected infection. Previous scrub typhus studies have utilized inbred mice, yet such models have intrinsic limitations. Thus, the development of suitable mouse models that better mimic human diseases is in great need for immunologic investigation and future vaccine studies. This study is aimed at establishing scrub typhus in outbred CD-1 mice and defining immune biomarkers related to disease severity. CD-1 mice received *O*. *tsutsugamushi* Karp strain via the i.v. route; major organs were harvested at 2–12 days post-infection for kinetic analyses. We found that for our given infection doses, CD-1 mice were significantly more susceptible (90–100% lethal) than were inbred C57BL/6 mice (0–10% lethal). Gross pathology of infected CD-1 mouse organs revealed features that mimicked human scrub typhus, including pulmonary edema, interstitial pneumonia, perivascular lymphocytic infiltrates, and vasculitis. Alteration in angiopoietin/receptor expression in inflamed lungs implied endothelial dysfunction. Lung immune gene profiling using NanoString analysis displayed a Th1/CD8-skewed, but Th2 repressed profile, including novel biomarkers not previously investigated in other scrub typhus models. Bio-plex analysis revealed a robust inflammatory response in CD-1 mice as evidenced by increased serum cytokine and chemokine levels, correlating with immune cell recruitment during the severe stages of the disease. This study provides an important framework indicating a value of CD-1 mice for delineating host susceptibility to *O*. *tsutsugamushi*, immune dysregulation, and disease pathogenesis. This preclinical model is particularly useful for future translational and vaccine studies for severe scrub typhus.

## Author summary

Scrub typhus is a severely neglected and potentially fatal disease caused by *Orientia tsutsugamushi*, a genetically intractable, obligately intracellular bacterium that annually infects at least one million people worldwide. There is no vaccine available, and our current understanding of the host immunological response and mechanisms remains very limited. Appropriate animal models of infection that recapitulate the disease are essential to the development of effective therapeutics and vaccines. In this study, we characterized the immunologic responses by transcriptomics and Bio-plex assays in outbred CD-1 mice with lethal *O*. *tsutsugamushi* infection. We found that CD-1 mice were highly susceptible to infection and that the high mortality correlated with a Th1/CD8-skewed, but Th2 repressed, immune profile during the acute phase of disease. This proinflammatory state was further confirmed by elevated cytokine and chemokine levels in the sera. Collectively, this study established CD-1 mice as a practical, preclinical model to define pathogenic mechanisms underlying severe scrub typhus and for future immunologic and translational studies during *O*. *tsutsugamushi* infection.

## Introduction

Scrub typhus is caused by *Orientia tsutsugamushi* (*Ot*), a Gram-negative, lipopolysaccharide-negative intracellular bacterium that preferentially replicates within the cytosol of endothelial cells and phagocytes (monocytes, macrophages, and dendritic cells) [1]. This vector-borne pathogen causes at least one million new cases per year, with a population of one billion at risk of infection [1,2]. Traditionally, scrub typhus is confined to a large area of Southeast Asia, known as the ‘tsutsugamushi triangle’ [1–3], with recent reemergence of cases in countries within this geographic region, including areas of India, Nepal, Micronesia, and the Maldives [4]. *Ot* distribution, however, has been recently reported far outside of this traditional region, such as in the Middle East [5], South America [6–8], and Africa [9–11]. Following exposure to chigger bites, some individuals exhibit an eschar at the inoculation site, and other early signs include fever and flu-like symptoms [12]. While many of these cases are self-limiting, patients with severe scrub typhus can develop interstitial pneumonia, hepatic inflammation, and meningoencephalitis [13]. These lesions may lead to acute lung damage, including edema, hemorrhage, diffuse alveolar damage, and interstitial cellular infiltration [14]. This acute tissue damage can lead to multi-organ failure, which is the cause of fatalities. Effective treatment consists of early use of antibiotics, such as doxycycline, but delayed or missed diagnosis due to its non-specific symptoms and lack of appropriate diagnostic equipment can result in up to 30% fatality rate [12]. Furthermore, reports of antibiotic treatment failure exacerbate the issue [15,16]. There is no approved vaccine available.

Murine models of *Ot* infection have been used as far back as 1948 for cross-vaccination tests [17] mostly via the intraperitoneal (i.p.) route of inoculation [18–20]. Since then, the development of more relevant scrub typhus models via the intradermal (i.d., the natural method of entry) or intravenous (i.v., representing hematogenous spread) routes of inoculation in inbred strains of mice have greatly enhanced our understanding of *Ot*-host interactions and disease pathogenesis [21–31]. Studies with inbred C57BL/6 (B6) and BALB/c mice have provided a stable and consistent background to investigate *Ot* virulence and immunological responses. The use of inbred mice is advantageous for many pilot studies that rely on low in-group variabilities. Despite these advantages, there is an ever present need to characterize how genetic diversity influences the host immunity during infection and vaccination studies, and to better mimic human responses. Such need is highlighted in a study by Martin *et al*. indicating that after protection and infection, the memory CD8 T cell pool size and rate of phenotypic progression were highly variable in individual outbred vs. inbred mice [32]. In some infection systems, inbred mice have been found to be intrinsically less resistant to bacterial and viral challenge than outbred mice, causing a confined view of an infection response [33–36]. Outbred mice have historically been used for pharmacological, toxicology, aging, and cancer studies [37]. They are also valuable in therapeutic and vaccine studies for other infectious diseases because of their broader genetic diversities [37].

Outbred CD-1 Swiss Webster (CD-1) mice, for example, have been recently used for vaccination studies against bacterial, viral, and parasitic infections [33–37]. Their use in the context of *Ot* immunology remains limited, although there are reports that use CD-1 mice to examine bacterial dissemination either via chigger bites [30,38–42] or needle-based i.d. inoculation [29,43], or to compare virulence among different *Ot* strains/isolates following i.p. inoculation [19,29,30]. To date, only one publication has used CD-1 mice for an *Ot* vaccine-related study [44]. For murine models of scrub typhus research, immunological studies have mostly focused on inbred models [45–47]. The wild-type and gene-targeted knockout mice on the B6 background have provided us with new insights into the magnitude and kinetics of host immune responses at the tissue and cellular levels during infection [27,45,48–51]. However, the CD-1 mouse model has not been extensively utilized, and comprehensive immune profiling is still lacking. Currently available studies have only examined basic antibody responses, lymph node sizes, cytokine profiles, cytokine-producing T-cells, and general pathology during lethal and non-lethal infections with different strains (i.p. and i.d. routes) [29,39,42,43]. Nevertheless, these earlier reports have helped pave the way for studies to understand CD-1 mice immunologic responses to *Ot* infection. This has led us to investigate CD-1 mouse immune responses, using our established methods for acute scrub typhus models in B6 mice (i.v. route) [46,48]. Since lethally infected B6 mice show a Th1-skewed, but Th2-suppressed, inflammatory response during late stages of acute disease, we sought to validate if this polarized immune response is specific to *Ot*-infected B6 mice or if this is a hallmark for lethal scrub typhus across mouse models.

In this study, we evaluate the benefit of using an outbred mouse model of infection with *Ot* Karp strain to explore the immune response profiles and biological hallmarks of severe scrub typhus. We first compared the susceptibility of CD-1 and B6 mice to infection and confirmed the hyper-susceptibility of CD-1 mice, as these mice completely succumbed by day 12 post-infection. Bacterial burdens were prevalent throughout major organs, showing pathological lesions resembling that in scrub typhus patients. Secondly, we performed an immunologic differential expression analysis in CD-1 mice during infection and revealed a highly Th1/CD8-skewed immune response during the acute phase of disease. These findings were complemented through quantification of relevant mRNA levels in the tissues via qRT-PCR, as well as proinflammatory cytokines and chemokines in the serum by using a Bio-Plex assay. To our knowledge, this is the first report of an i.v. CD-1 mouse model of *Ot* infection, allowing for a comprehensive study for biological hallmarks of severe scrub typhus. This study will broaden our understanding of scrub typhus pathogenesis and open new areas of mechanistic investigation.

## Materials and methods

### Ethics statement

The University of Texas Medical Branch (UTMB) complies with the USDA Animal Welfare Act (Public Law 89–544), the Health Research Extension Act of 1985 (Public Law 99–158), the Public Health Service Policy on Humane Care and Use of Laboratory Animals, and the NAS Guide for the Care and Use of Laboratory Animals (ISBN-13). UTMB is a registered Research Facility under the Animal Welfare Act. It complies with NIH policy and has current assurance with the Office of Laboratory Animal Welfare. All procedures were approved by the Institutional Biosafety Committee, in accordance with Guidelines for Biosafety in Microbiological and Biomedical Laboratories. Infections were performed following Institutional Animal Care and Use Committee approved protocols (2101001 and 1902006) at UTMB in Galveston, TX.

### Mouse infection and organ collection

Female Swiss Webster CD-1 outbred mice were purchased from Envigo (East Millstone, NJ). Female B6 mice were purchased from Jackson Laboratory (Bar Harbor, ME). Mice were maintained under specific pathogen-free conditions in the same room for 9–10 days and infected at 8–12 weeks of age. Infections were performed in the Galveston National Laboratory ABSL3 facility at UTMB. All tissue processing and analytic procedures were performed in BSL3 or BSL2 laboratories, respectively. All infections were performed using the same bacterial stock of *Ot* Karp strain prepared from Vero cells, as described in our previous studies [46,50]. Two independent studies were performed in which mice were inoculated i.v. with approximately equivalent lethal doses of 5.6 ×10^4^ or 4.32 × 10^4^ focus forming units (FFU) of the same Karp stock (200 μl), or with PBS as negative controls; our used dose ranges resulted in near identical infection outcomes and immune profiles. Mice were monitored daily for body weight, signs of disease, and disease scores. The disease score (ranged from 0–5) was based on an approved animal sickness protocol. The criteria included mobility/lethargy, hunching, fur ruffling, bilateral conjunctivitis, and weight loss: 0-normal behavior; 1- active, some weight loss (<5%); 2- weight loss (6–10%), some ruffled fur (between shoulders); 3- weight loss (11–19%), pronounced ruffled fur, hunched posture, erythema, signs of reduced food/water taken; 4- weight loss (20–25%), decreased activity, bilateral conjunctivitis, showing signs of incapable to reaching food/water; 5- non-responsive (or weight loss of greater than 25%) animal need to be humanely euthanized. Serum and tissue samples (lung, brain, liver, spleen, and kidney) were collected at days 4, 6, and 10 for the first study, and at days 2, 4, 6, 8, 10, and 12 for the second study. Samples (3-4/time point) were inactivated for immediate and subsequent analysis, with mock samples serving as the controls.

### Bacterial load quantification

Animal tissues were collected and stored in RNA*Later* (Qiagen) at 4°C overnight for inactivation and then stored at -80°C. DNA was extracted using the DNeasy Blood & Tissue Kit (Qiagen) following the manufacturer’s instructions. Less than or equal to 30 mg of tissue was used for each extraction. Tissue bacterial burdens were quantified via qPCR and normalized to total nanogram (ng) of DNA per μL of sample as described previously [48]. Data were expressed as the copy number of 47-kDa gene per ng of DNA. The copy number for the 47-kDa gene was determined by serial dilution of known concentrations of a control plasmid containing a single-copy insert of the gene.

### Nanostring gene expression profiling

Lung tissues were collected and stored in RNA*Later* (Qiagen) at 4°C overnight for inactivation and then stored at -80°C. Total RNA was extracted from mock and *Ot*-infected tissues at days 4, 8, and 12 (3 samples/group) using the RNeasy Kit (Qiagen), according to the manufacturer’s instructions. Samples were processed at the Baylor College of Medicine Genomic and RNA Expression Profiling Core (Houston, TX). The Nanostring nCounter platform and the Mouse Immunology Panel were used to quantify transcripts of 561 immune genes plus 14 housekeeping genes (NanoString Technologies, Seattle, WA). Gene expression was normalized to housekeeping gene expression, and the data were analyzed using the nSolver Software Version 4 and Advanced Analysis Version 2.0 (NanoString Technologies), as described in our previous study [52].

### Quantitative reverse transcriptase PCR (qRT-PCR)

Total RNA was extracted from tissues using the RNeasy mini kit (Qiagen) and treated with DNase according to the manufacturer’s protocol (Qiagen). cDNA was synthesized via the iScript cDNA synthesis kit (Bio-Rad Laboratories). Target gene abundance was measured by qRT-PCR using a Bio-Rad CFX96 real-time PCR apparatus. SYBR Green Master mix (Bio-Rad) was used for all PCR reactions. The assay included: denaturing at 95°C for 3 min followed with 40 cycles of: 10s at 95°C and 30s at 60°C. The 2^−ΔΔCT^ method was used to calculate relative abundance of mRNA expression. Glyceraldehyde-3-phosphate dehydrogenase (GAPDH) was used as the housekeeping gene for all analyses. Primer sequences used are listed in S1 Table.

### Histology

All tissues were fixed in 10% neutral buffered formalin and embedded in paraffin. Tissue sections (5 μm thickness) were stained with hematoxylin and eosin and mounted on slides, as in our previous reports [46,48]. Images were captured by using the cellSens Software (Olympus).

### Serum cytokine and chemokine levels

Whole blood was collected from CD-1 and B6 mice at days 2, 4, 6, 8, 10, and 12 of infection and compared with mock controls. Serum was isolated and inactivated, as described in our previous study [46]. The Pro Mouse Cytokine 23-Plex Kit (Bio-Rad) was used to measure cytokine and chemokine levels. The Bio-Rad Bio-Plex Plate Washer and Bio-Plex 200 machines were used for sample processing and analysis. All processes were completed following the manufacturer’s instructions.

### Statistical analyses

Gene expression profiling data were analyzed using the nSolver Software (NanoString Technologies) and presented graphically as mean ± standard error of the mean (SEM). Adjusted *p*-values were obtained using the Benjamini-Yekutieli procedure to test for significance. Data presented from survival curves, qPCR and qRT-PCR assays were analyzed using GraphPad Prism software. qPCR and qRT-PCR assays are presented as mean ± SEM. Differences between control and infected groups were analyzed using Student’s t test and one-way ANOVA (parametric and non-parametric) where appropriate. Differences between survival curves were analyzed using the Log-ranked (Mantel-Cox) test. Statistically significant values are denoted as *p < 0.05, ** *p* < 0.01, *** *p* < 0.001, and **** *p* < 0.0001, respectively.

## Results

### High susceptibility of CD-1 mice to *Ot* infection

**Fig 1:**
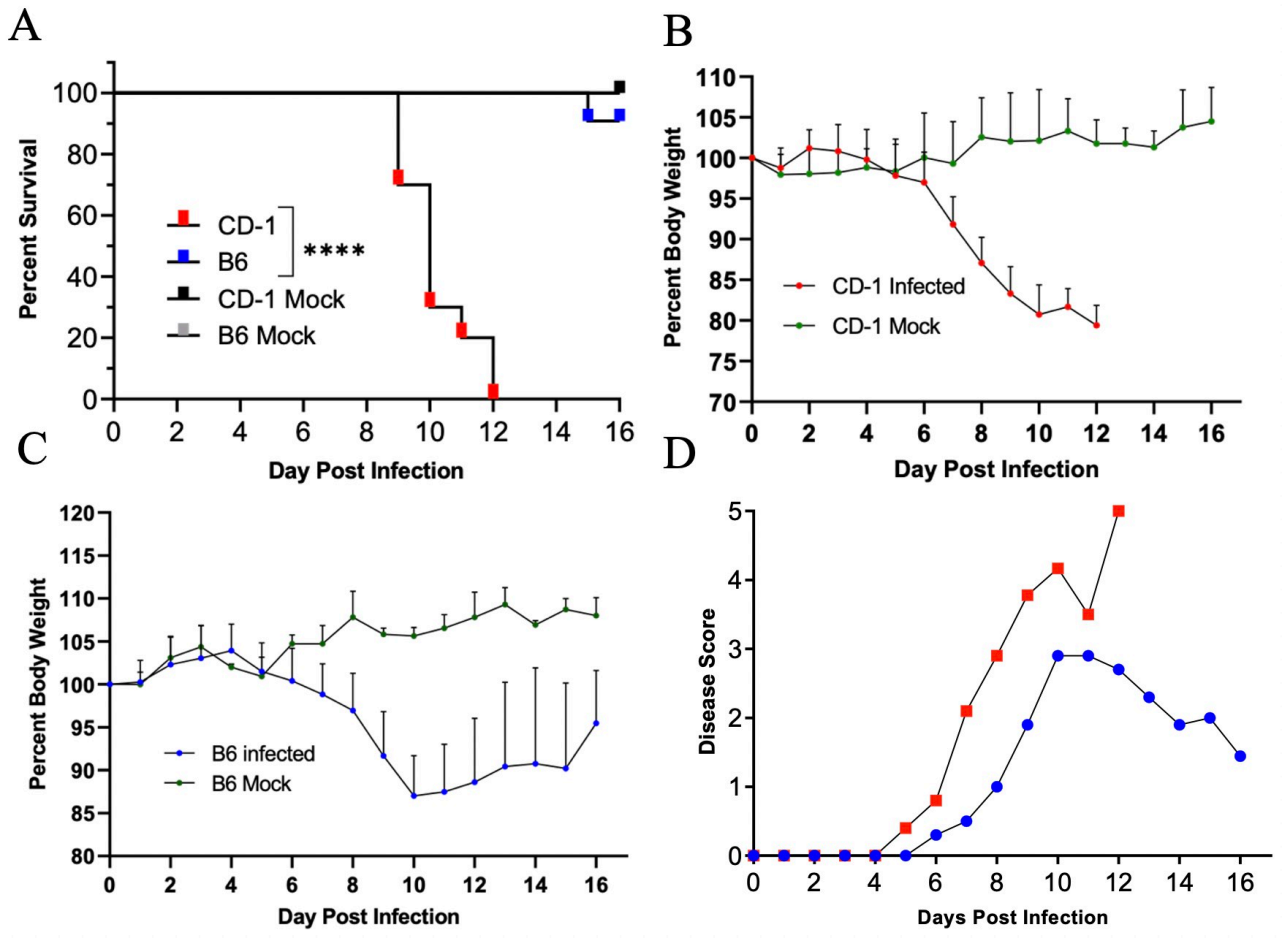
Outbred CD-1 mice were highly susceptible to *O*. *tsutsugamushi* infection. CD-1 and C57BL/6 mice were inoculated i.v. with *O*. *tsutsugamushi* Karp strain (5.6×10^4^ FFU, 10/group) or PBS (mock, 3/group) and monitored daily for signs of illness. (**A**) CD-1 mice began to succumb to infection on day 9, and all died by day 12. Only 10% of C57BL/6 mice died by day 15, with the rest surviving. (**B, C**) Body weight loss in CD-1 mice was rapid and progressive, in comparison to that of C57BL/6 mice. (**D**) CD-1 mice showed disease score on day 5 and had the highest score on day 12. In contrast, C57BL/6 mice started to recover at day 11. Log-ranked (Mantel Cox) test was used for statistical analysis of survival. ****, *p* < 0.0001.

**Fig 2:**
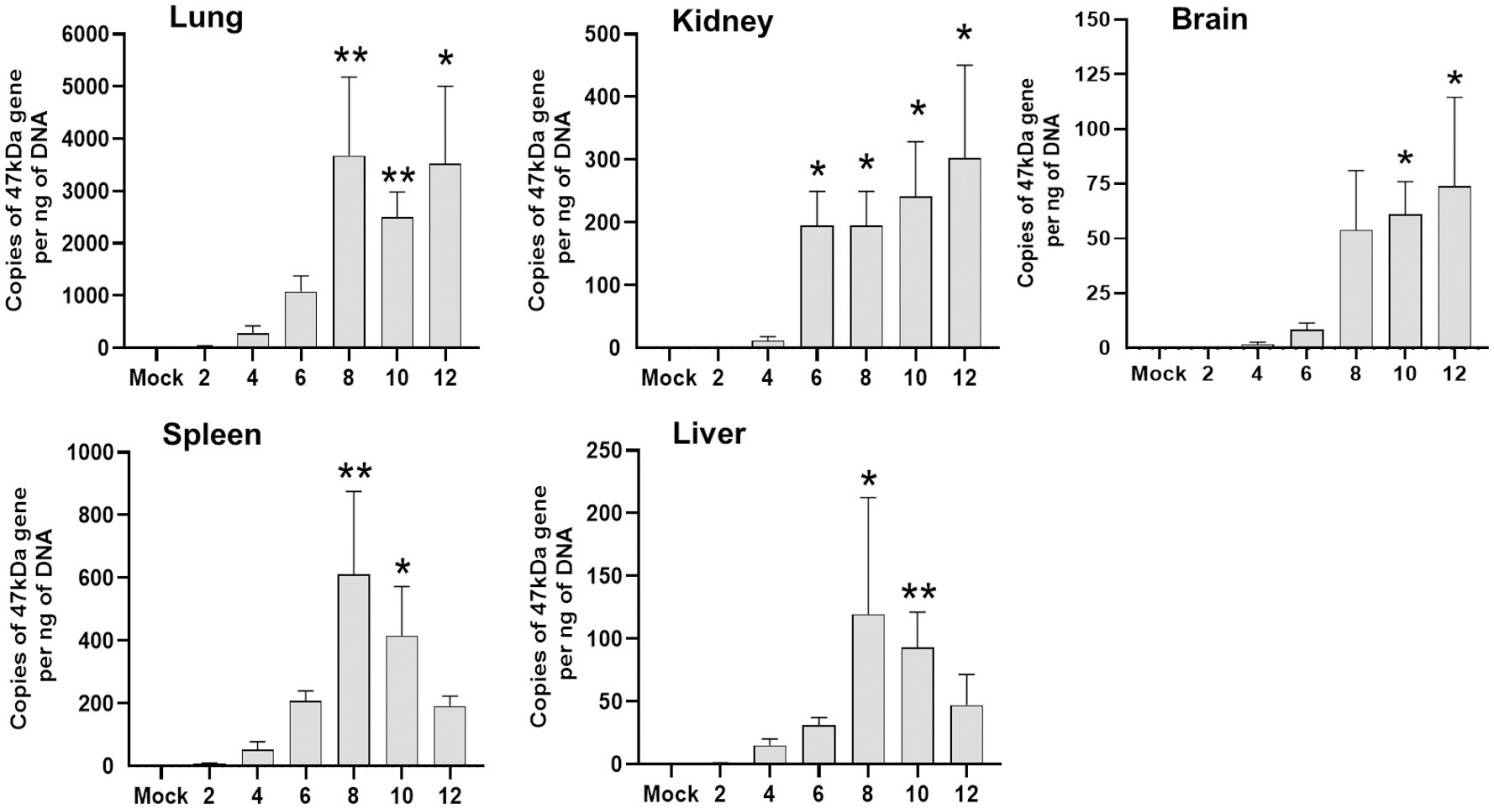
Organ bacterial burden in CD-1 mice following infection with *O*. *tsutsugamushi*. CD-1 mice were inoculated i.v. with *O*. *tsutsugamushi* Karp strain (4.32×10^4^ FFU) or PBS (mock). At indicated days post-infection, organs were collected (3-4/group) for DNA extraction and bacterial burden analysis by qPCR. Data are presented as mean ± SEM. One-way ANOVA (non-parametric) was used for statistical analysis. *, *p* < 0.05; **, *p* < 0.01.

To-date, no reported studies have investigated the susceptibility of CD-1 and B6 mice in parallel via the i.v. route of *Ot* inoculation. We addressed this by performing a side-by-side infection with both mouse strains and monitoring for signs of disease. After i.v. injection with 5.6 ×10^4^ FFU of bacteria, CD-1 mice began to succumb to infection by day 9 (30%) and were completely moribund by day 12 (Fig 1A). In contrast, only 10% of B6 mice succumbed on day 15 and the remaining mice (90%) recovered and survived (Fig 1A). Body weight loss began in CD-1 and B6 mice on days 5 and 7, respectively, and continued in both groups until day 10. While B6 mice began to gain weight from day 11 through day 16, CD-1 mice briefly plateaued until they succumbed to disease by day 12 (Fig 1B and 1C). The weight loss patterns coincided closely with those of disease scores (Fig 1D). Lung tissues from B6 and CD-1 mice revealed a sharp contrast in bacterial burdens (S1 Fig). Higher bacterial burden in CD-1 mice than in B6 mice was observed at day 4 and was more evident by day 6. At day 10, lung bacterial burden in CD-1 mice had a sharp increase, while those in B6 mice appeared to be under control. This statistically significant difference in lung bacterial burden at day 10 closely correlated with our observed trends in disease scores and body weight. It is important to mention that this inoculation dose (5.6 × 10^4^ FFU) was much lower than that previously used in our reports of lethal B6 models (1.25 × 10^6^ FFU) [46,48], in which tissue bacterial burdens reached a peak at day 6 [46]. As shown in Fig 2 during a detailed study of CD-1 mice (4.32×10^4^ FFU), while virtually no bacteria were detected at days 2 and 4, bacterial burdens were significantly increased at day 6 (kidneys). By day 8, bacterial burdens were significantly increased in nearly all organs (lungs, kidneys, spleen, liver), except for the brain (which reached statistical significance at day 10). The lung and brain samples contained the highest vs. the lowest bacterial burdens, respectively. While spleen and liver bacterial burdens reduced to non-significant levels at day 12, other organs (lungs, kidney, and brain) maintained significantly high levels of bacteria (*p* < 0.05). These results corroborated our previous reports utilizing a lethal B6 model [46,48]. These findings indicate a systemic infection in CD-1 mice, but more importantly, a significantly higher susceptibility of CD-1 mice to *Ot* infection than B6 mice.

### Pathological changes and endothelial dysfunction during acute infection in CD-1 mice

**Fig 3:**
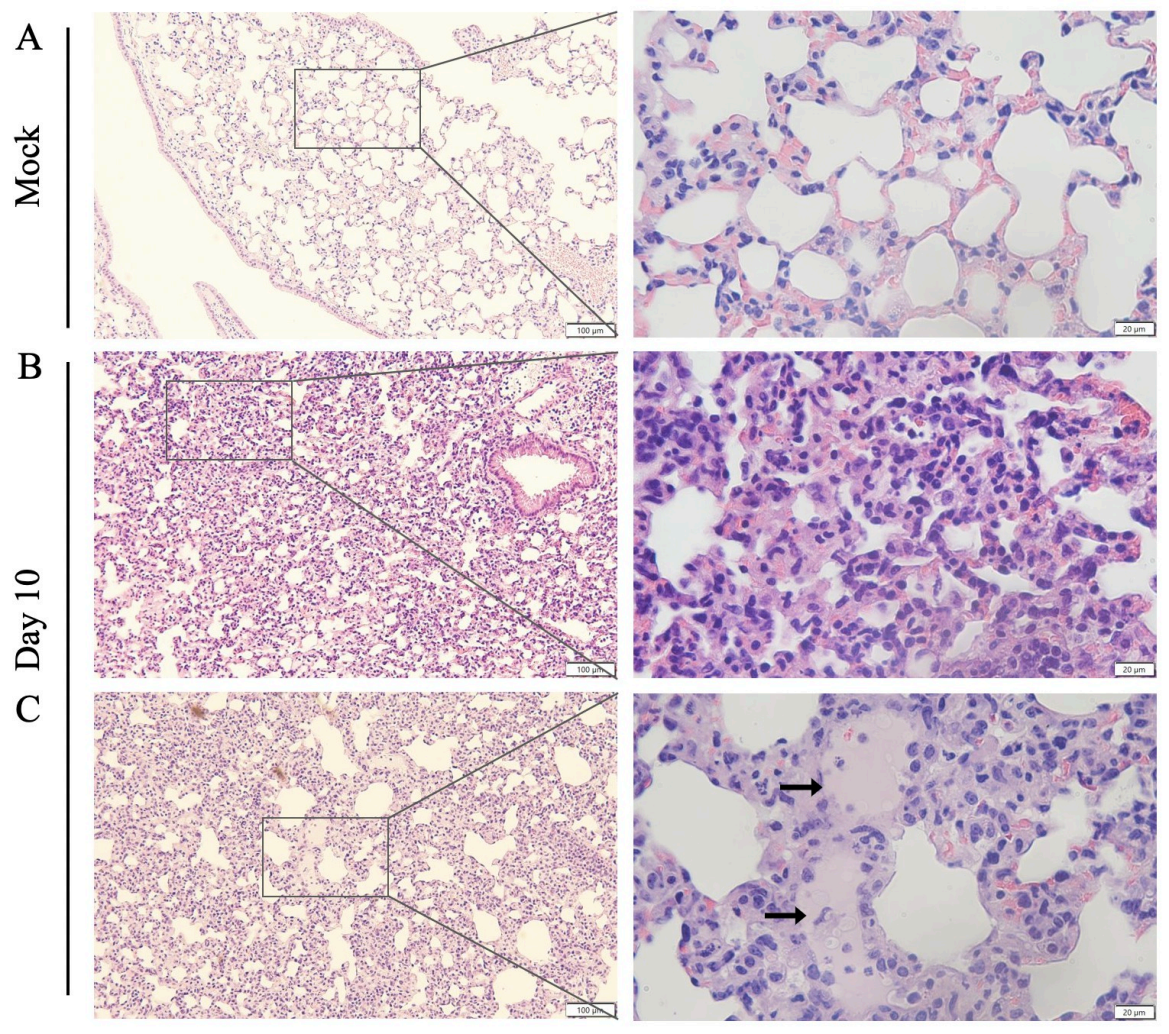
Cellular infiltration and tissue injury in *O*. *tsutsugamushi-*infected CD1 mice. CD-1 mice were infected, as described in Fig 1. Lung tissues (3–4 mice/group) were collected from mock (**A**) and at day 10 and subjected to hematoxylin and eosin staining. Cellular infiltration was present with interstitial pneumonia (**B, C**) and pulmonary edema (arrows) observed in the lung tissues of infected mice. Scale bars: 100 μm and 20 μm (zoomed).

**Fig 4:**
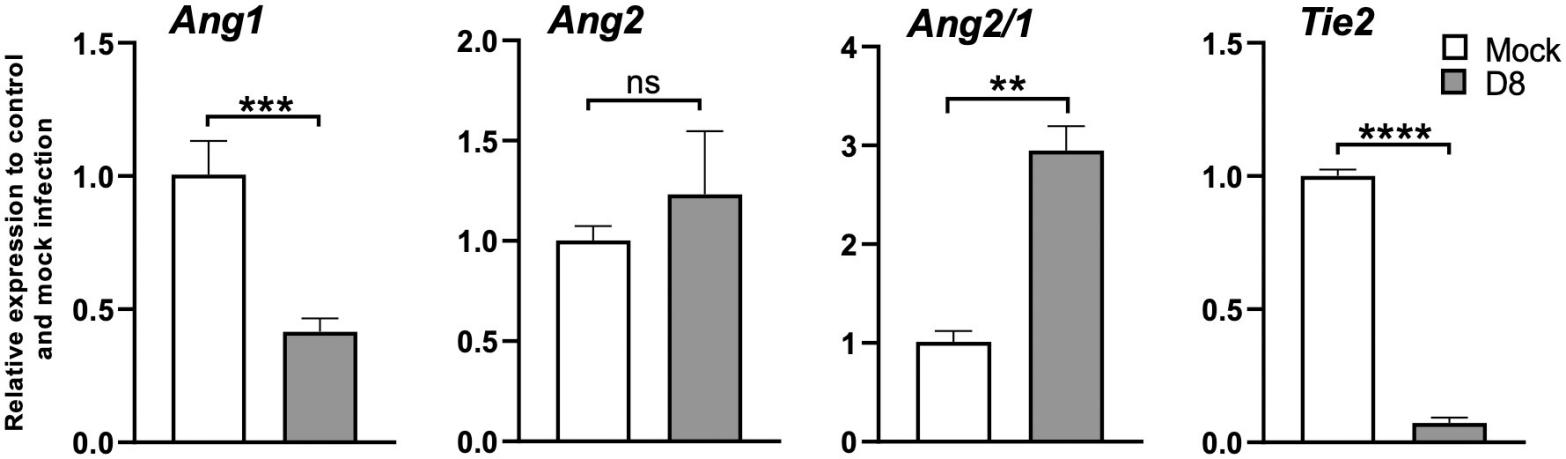
Lung endothelial dysfunction in CD-1 mice during acute infection. CD-1 mice were infected, as described in Fig 2. Lung tissues were collected from mock and day 8 infected mice (3–4 mice/group), and RT-qPCR assays were performed to measure *Ang1*, *Ang2*, and *Tie2* levels, shown as relative to GAPDH values. The Ang2/Ang1 ratio of individual samples was calculated based on RT-qPCR data and compared to mock controls. Data are presented as mean ± SEM. Students T-test was used for statistical analysis. ns = not significant; **, *p* < 0.01; ***, *p* < 0.001; ****, *p* < 0.0001.

Pathologic findings of human scrub typhus commonly include disseminated endothelial injury and lymphohistiocytic vasculitis, leading to interstitial pneumonitis, encephalitis, and hepatic damage [53–55]. Our established inbred mouse models mimic certain pathologic features of severe human disease [27,46,48,50,51]. As compared to mock infection (Fig 3A), CD-1 mice exhibited interstitial pneumonia (Fig 3B and 3C) and pulmonary alveolar edema (Fig 3C) indicating increased vascular permeability, consistent with our previous lethal B6 models [46,48]. CD-1 mice also showed perivascular lymphocytic infiltrates and vasculitis in the liver (S2 Fig), as described in our previous reports [46,48], as well as multifocal mononuclear infiltrates in the cortex of the kidneys (S3 Fig). Since *Ot* shows a tropism for replication in endothelial cells [14,53], endothelial cell activation and injury can promote recruitment, adherence, and transmigration of immune cells for immune mediated pathogen clearance and increased vascular permeability leading to edema [56]. To promote endothelial cell quiescence and vascular barrier integrity in healthy microenvironments, constitutive angiopoietin-1 (Ang1) binds the Tie2 receptor on the cell surface [57]. During infectious or inflammatory processes, Ang2 is induced and disrupts this axis, binding to Tie2 and promoting vascular barrier destabilization [58]. Therefore, the levels of *Ang1*, *Ang2* and *Tie2* transcripts can be used to evaluate endothelial activation/injury during infection. As shown in Fig 4, lung *Ang1* and *Tie2* levels were significantly decreased on day 8, while *Ang2* was insignificantly increased. The *Ang2/Ang1* ratio showed a statistically significant difference between day 8 and mock samples (Fig 4). This indication of endothelial dysfunction and inflammation agrees with our previous findings in the lethal B6 mouse model [48,50,51]. Together, our study demonstrates that the CD-1 infection model mimics certain pathologic lesions of severe human scrub typhus in humans and inbred mouse models, offering additional value to examine biomarkers of host susceptibility and immune alterations.

### Immune profile of *O*. *tsutsugamushi*-infected lungs during acute infection

**Fig 5:**
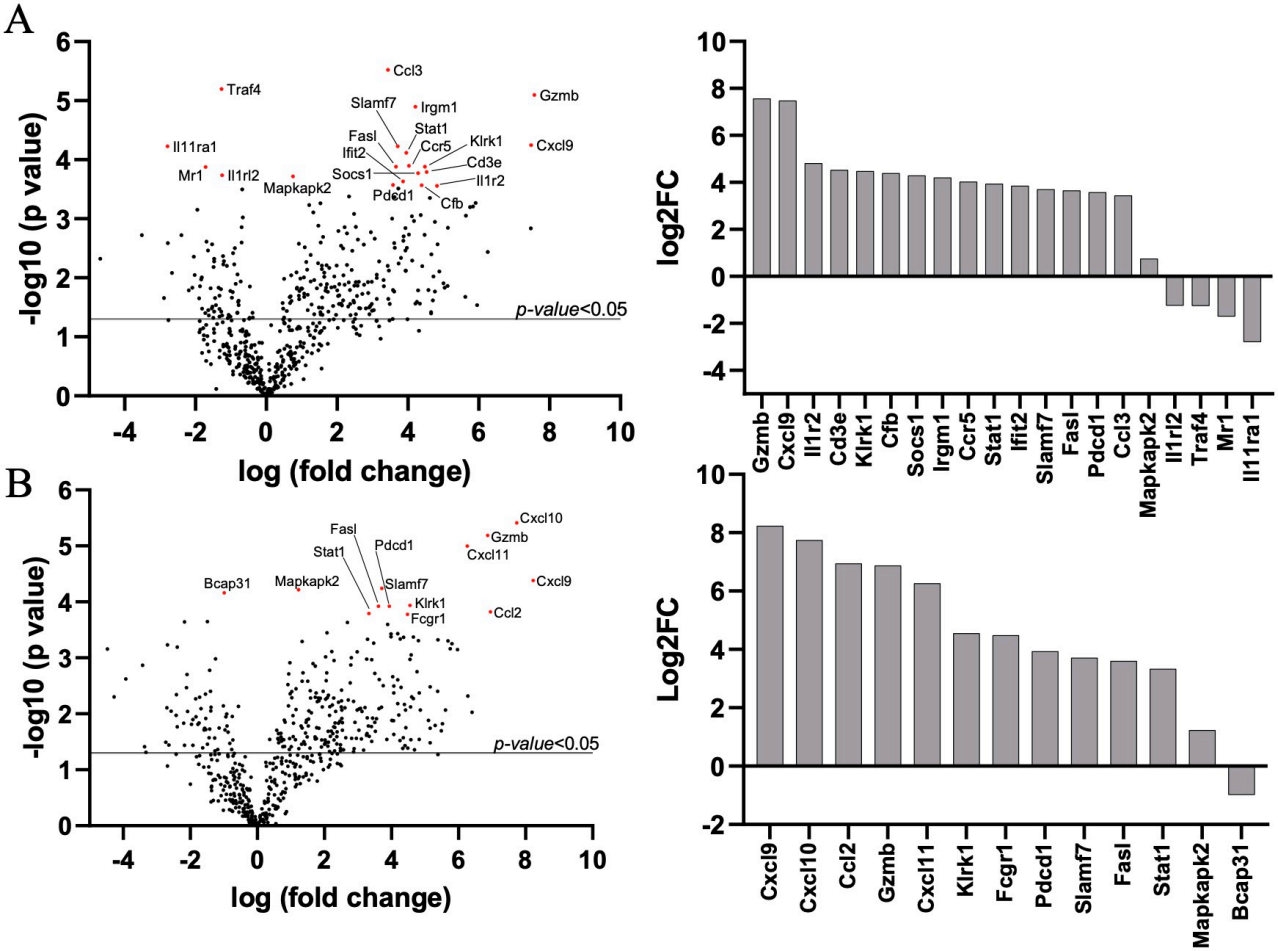
Lung transcriptomic profiling during *O*. *tsutsugamushi* infection. CD-1 mice were infected, as described in Fig 2. Lung tissues were collected at different times (0, 4, 8, and 12 days, 3–4 mice/group) and used for total RNA extraction and Nanostring analyses. At 8 days (**A**) and 12 days (**B**), Volcano plots show significantly up- or down-regulated expression of genes (*p-value* <0.05) in black above the solid line, and significantly up- or down-regulated expression of genes with *adjusted p-value* <0.05 in red or presented in column graphs shown as Log2fold change (Log2FC).

**Fig 6:**
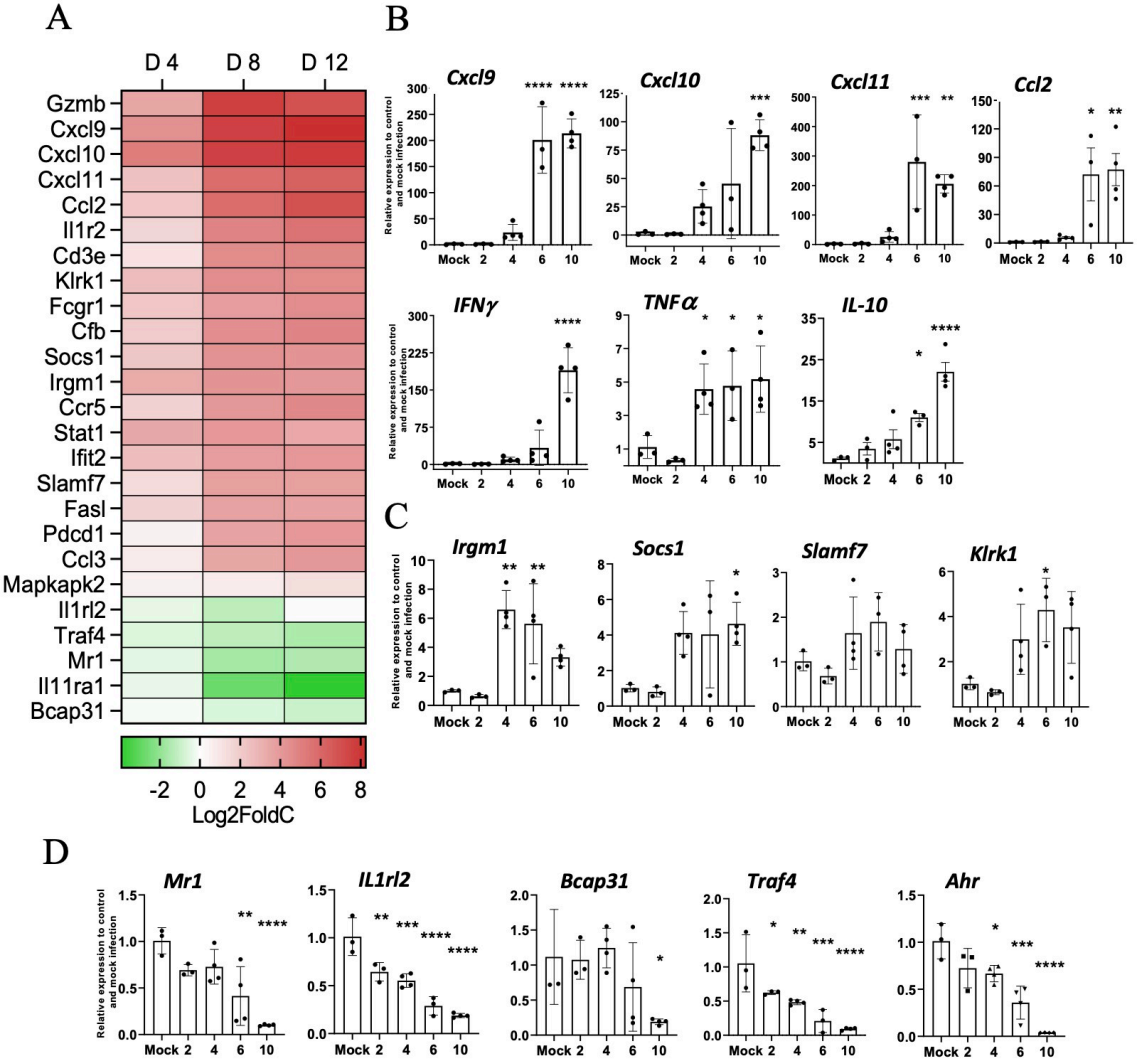
CD-1 mice displayed type 1-skewed immune profiles during acute infection. Lung transcriptomic profiling analyses were performed, as described in Fig 5. (**A**) NanoString data show Th1 and Th2 signature genes (in Log2Fold change) at indicated days post-infection as compared to mock samples. Lung qRT-PCR assays were performed and shown as relative to GAPDH values highlighting pro-inflammatory (**B**) and novel upregulated (**C**) and downregulated (**D**) markers. Data are presented as mean ± SEM. One-way ANOVA was used for statistical analysis. *, *p* < 0.05; **, *p* < 0.01; ***, *p* < 0.001; ****, *p* < 0.0001.

To gain a broad picture of immune responses in the lung during *Ot* infection in CD-1 mice, we performed pulmonary differential expression analysis on 562 immunology-related genes via NanoString. After injection with 4.32 ×10^4^ FFU of bacteria, tissues from days 4, 6, and 12 were analyzed, with mock animals serving as the baseline. Day 4 samples did not show any statistically significant changes in gene expression (see S2 Table for complete list), which was not surprising, given the relatively low bacterial burden in tested organs (Fig 2, [48]), disease course (Fig 1D, [46]), and minimal immune responses at early stages of infection [46,48,59]. As shown in Fig 5A and Table 1, day 8 samples had 16 upregulated and 4 downregulated genes of statistical significance (adj. *p-value* <0.05). At day 12, we found significantly upregulated expression of 12 genes and 1 significantly down-regulated gene (adj. *p-value* <0.05) (Fig 5B, Table 2). The expression kinetics of infected lungs displayed Th1-skewed immune responses during disease progression with markedly increased levels of *Cxcl9*, *Cxcl10*, *Cxcl11*, and *Gzmb* (Fig 6A). Other cytotoxicity-associated genes, including *Slamf7*, *Fasl*, and *Klrk1*, were upregulated at both days 8 and 12 (Tables 1 and 2). Meanwhile, *Il11ra1*, *Traf4*, *Illrl2*, and *Mr1* were downregulated at day 8, while *Bcap31* was downregulated at day 12 (Tables 1 and 2). Inflammatory markers identified through our differential expression analysis were verified through qRT-PCR, confirming the high expression of *Cxcl9*, *Cxcl10*, *Cxcl11*, *IFNγ*, and *TNFα* on day 10 (Fig 6B). Selectively upregulated or downregulated genes were also verified through qRT-PCR (Fig 6C and 6D), respectively. Our findings of type 1-skewed responses in lethal CD-1 models corroborated with our previous reports in the lethal B6 models [27,48,50,52].

**Table 1:** Differentially expressed genes filtered by adjusted test statistic (D8 vs. Mock).

Gene	Name/Encoded Protein	Log2 fold change	P-value	B.Y. P-value
*Gzmb*	Granzyme B	7.57	0.00000798	0.01
*Cxcl9*	C-X-C Motif Chemokine Ligand 9	7.48	0.00005644	0.03
*Il1r2*	Interleukin 1 Receptor Type 2	4.82	0.0002755	0.05
*Cd3e*	T-Cell Surface Glycoprotein CD3 Epsilon Chain	4.53	0.00016095	0.05
*Klrk1*	Killer Cell Lectin Like Receptor K1	4.48	0.00013152	0.04
*Cfb*	Complement Factor B	4.39	0.00027052	0.05
*Socs1*	Suppressor Of Cytokine Signaling 1	4.29	0.00016887	0.05
*Irgm1*	Immunity-related GTPase family M protein 1	4.21	0.00001265	0.01
*Ccr5*	C-C Motif Chemokine Receptor 5	4.03	0.00012622	0.04
*Stat1*	Signal Transducer and Activator of Transcription 1	3.95	0.00007652	0.04
*Ifit2*	Interferon-Induced Protein with Tetratricopeptide Repeats 2	3.86	0.00023174	0.05
*Slamf7*	SLAM Family Member 7	3.71	0.000059	0.03
*Fasl*	Fas Cell Surface Death Ligand	3.66	0.00013148	0.04
*Pdcd1*	Programmed Cell Death 1	3.58	0.0002674	0.05
*Ccl3*	C-C Motif Chemokine Ligand 3	3.44	0.00000299	0.01
*Mapkapk2*	MAPK Activated Protein Kinase 2	0.75	0.0001917	0.05
*Il1rl2*	Interleukin 1 Receptor Like 2	-1.25	0.00018397	0.05
*Traf4*	TNF Receptor Associated Factor 4	-1.26	0.00000635	0.01
*Mr1*	Major Histocompatibility Complex, Class I-Related	-1.71	0.00013216	0.04
*Il11ra1*	Interleukin 11 Receptor Subunit Alpha chain 1	-2.79	0.00005951	0.03

**Table 2:** Differentially expressed genes filtered by adjusted test statistic (D12 vs. Mock).

Gene	Name/Encoded Protein	Log2 fold change	P-value	BY P-value
*Cxcl9*	C-X-C Motif Chemokine Ligand 9	8.23	0.00004141	0.04
*Cxcl10*	C-X-C Motif Chemokine Ligand 10	7.74	0.00000386	0.01
*Ccl2*	C-C Motif Chemokine Ligand 2	6.95	0.00015106	0.05
*Gzmb*	Granzyme B	6.87	0.00000649	0.01
*Cxcl11*	C-X-C Motif Chemokine Ligand 10	6.26	0.0000101	0.01
*Klrk1*	Killer Cell Lectin Like Receptor K1	4.55	0.00011555	0.05
*Fcgr1*	Fc Fragment of IgG Receptor I	4.48	0.0001675	0.05
*Pdcd1*	Programmed Cell Death 1	3.94	0.00011924	0.05
*Slamf7*	SLAM Family Member 7	3.71	0.00005722	0.04
*Fasl*	Fas Cell Surface Death Ligand	3.61	0.00012019	0.05
*Stat1*	Signal Transducer and Activator of Transcription 1	3.33	0.00016157	0.05
*Mapkapk2*	MAPK Activated Protein Kinase 2	1.23	0.00006074	0.04
*Bcap31*	B Cell Receptor Associated Protein 31	-0.99	0.00006868	0.04

### Other potentially important biomarkers of immune dysregulation

Considering the inherent variability associated with an outbred mouse model [37], we also parsed our gene profiling data for differentially expressed markers deemed significant (*p* < 0.05), based on unadjusted test statistics. Lung tissue expression of endothelial activation markers and receptors *VCAM-1*, *ICAM-1*, *E-selectin*, *Itgam*, *Itgal*, *Itga4*, and *Itgb2*, as well as endothelial-associated markers *Cd6* and *L-selectin*, were upregulated day 12, suggesting endothelial activation and damage (S4 Fig), which were consistent with our previous reports of lethal B6 models [50]. Furthermore, expression of several cellular stress markers (*H60a*, *Cybb*, *Pdcd1*, *Fasl*, *Bid*, and *Fas*) and cytotoxicity-associated genes (*lcos*, *Ctla4*, *Klrc1*, and *Klrd1*) were upregulated, while macrophage scavenger (*MARCO*, and *Msr1*) and C-type lectin receptors (*Clec4e*, *Clec4a4*, *Clec5a*, *Card9)* were highly upregulated (S4 Fig). These new findings validated and expanded our recent report that Clec4e/Mincle plays an important role in sensing *Ot* and in stimulating type 1 cytokines and chemokines *in vivo* and *in vitro* [52]. In comparison, expression of Toll-like receptors was marginally or not upregulated (S4 Table). In contrast to Th1-related markers, expression of Th2-associated markers (*IL-5*, *IL-13*, *IL-9*, *IL-25*, and *Ahr*) was downregulated at day 12 (S4 Fig). Together, these findings correlate with our previous studies and reveal important biomarkers and/or pathways for future studies.

### Differential cytokine/chemokine levels in sera of infected CD-1 and B6 mice

**Fig 7:**
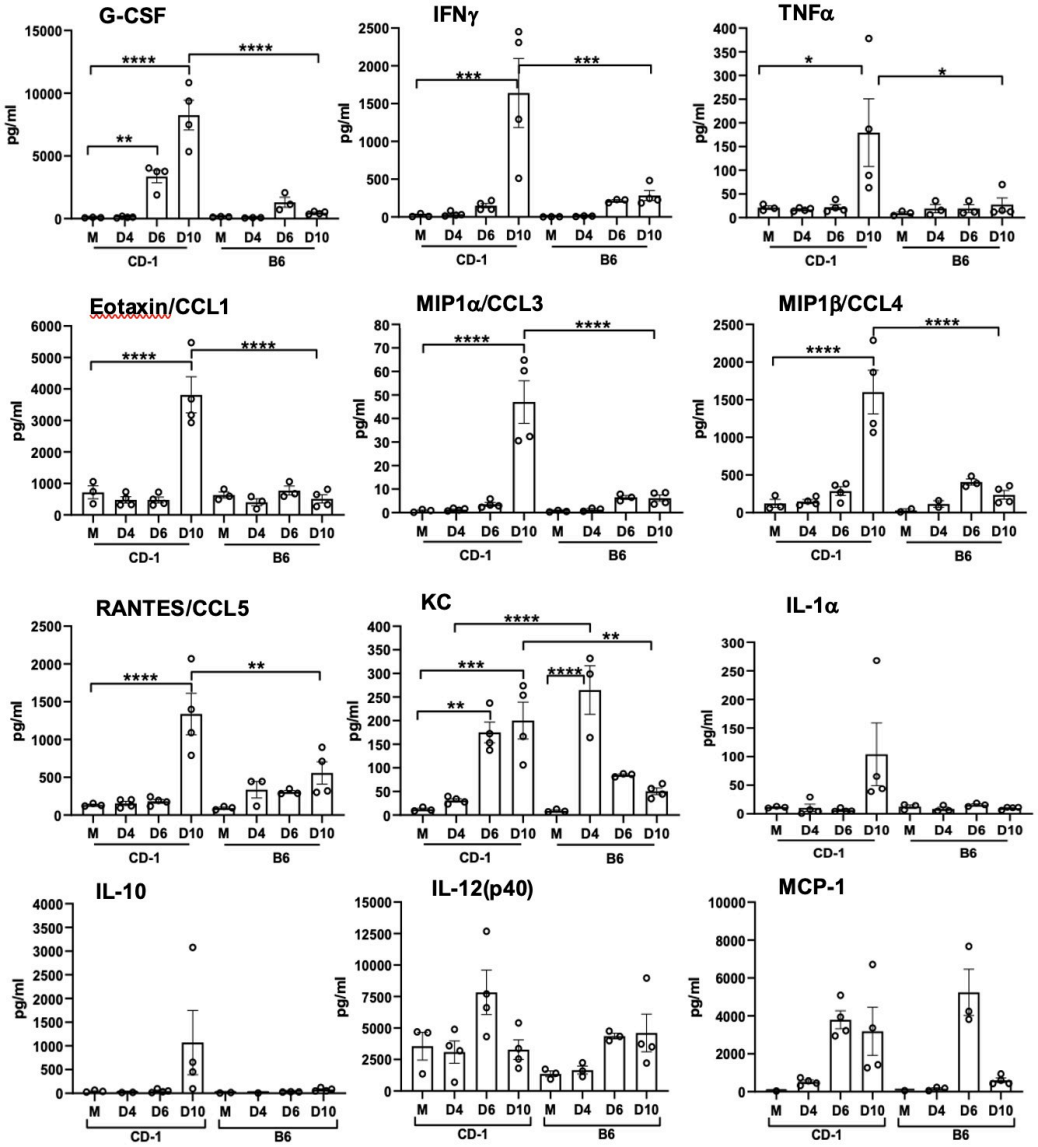
Serum cytokines and chemokines protein levels reveal proinflammatory responses in CD-1 mice versus C57BL/6 mice. CD-1 mice were infected, as described in Fig 1. Whole blood was collected for serum preparation (3–4 mice/group). A Bioplex assay was performed to determine cytokine and chemokine levels. Data are presented as mean ±SEM. One-way ANOVA was used for statistical analysis. *, *p* < 0.05; **, *p* < 0.01; ***, *p* < 0.001; ****, *p* < 0.0001.

Having documented differential susceptibilities between the two strains of mice, we then analyzed cytokine and chemokine protein levels in sera by using the Bio-Plex assay. As shown in Fig 7, CD-1 mice, but not B6 mice, showed statistically significantly elevated levels of G-CSF at days 6 and 10, as well as significantly high levels of cytokines and chemokines at day 10 (IFNγ, TNFα, Eotaxin/CCL1, MIP-1α/CCL3, MIP-1β/CCL4, RANTES/CCL5), implying high levels of proinflammatory responses at the late stages of infection in CD-1 mice. Of note, while KC levels were highly increased in both groups, the relatively resistant B6 mice had a significant increase in KC levels at day 4 (*p* < 0.0001). In contrast, susceptible CD-1 mice showed a significant increase in KC levels at days 6 and 10 (*p* < 0.01), revealing distinct expression kinetics of this neutrophil-recruiting chemokine. At day 10, there were statistically significant differences in levels of G-CSF, IFNγ, TNFα, Eotaxin/CCL1, MIP-1α/CCL3, MIP-1β/CCL4, RANTES/CCL5, and KC protein levels between CD-1 and B6 mice. The expression levels of IL-1α, IL-10, IL-12(p40), and MCP-1 were not significantly different. Other tested cytokines and chemokines were below detection limits, including IL-1β, IL-2, IL-3, IL-4, IL-5, IL-6, IL-9, IL-12(p70), IL-13, IL-17, and GM-CSF. Therefore, CD-1 mice generated a robust inflammatory response for cellular recruitment during the late/severe stages of the disease, in a magnitude that was much greater than similarly infected B6 mice.

## Discussion

Scrub typhus is one of the most neglected tropical infectious diseases. Obstacles to studying the biology and immunology of *Ot* bacteria have been persistent and impactful. Historically, animal model research on *Ot* has utilized the i.p. route of infection, which often causes progressive peritoneal inflammation [18,60]. Such an approach has intrinsic limitations and cannot accurately mimic lesions in human disease, the disease severity produced, and translational application to humans. Here, we provide the first report of a CD-1 mouse i.v. infection model of *Ot*, as well as several lines of key findings that are important to our understanding of scrub typhus immunology and disease pathogenesis.

First, we have shown that CD-1 mice are significantly more susceptible than B6 mice to a relatively low-dose of *Ot* inoculation, judged by progressive loss of body weight, elevated disease scores, and high mortality rates (Fig 1). The greater susceptibility of CD-1 mice was surprising, as we initially expected better infection control due to its outbred nature [33–36]. CD-1 mice did exhibit greater spectrum and levels of proinflammatory responses than B6 mice (Fig 7); however, host responses in CD-1 mice were either insufficient at the early stages (innate immunity) or imbalanced at the later infection stage (immune pathogenesis). Regardless of the mechanisms, our observation of mouse strain-related differences in susceptibility calls for investigation at the molecular, cellular, and organ levels. Along this line, a comparative study has indicated that CD-1-derived microglial cells demonstrated a higher scale of inflammatory responses than B6-derived microglial cells, determined by the mRNA expression levels of TNFα and IL-6 [61]. The pathogenic mechanisms responsible for the disparity in susceptibility between CD-1 and B6 mice warrant further investigation.

Second, it is evident this CD-1 mouse model closely resembles certain features of severe scrub typhus observed in human patients [54,55,62], as well as those seen in our previously reported lethal B6 mouse models (high dose inoculation) [46,48] regarding tissue bacterial distribution and burdens (Fig 2), pathological lesions (Fig 3), and endothelial cell dysfunction (Fig 4). While the highest and most sustained bacterial burdens were detected in the lungs, bacterial burdens decreased to insignificant levels in the spleen and liver by day 12. Similar *Ot* distribution trends across organs were observed in previous CD-1 mouse studies that employed mite bite [30,40] or i.p. injection [19], or in B6 and BALB/c mouse infection via the i.d. route [29,43]. Our findings of highest bacterial burdens in the lungs were consistent with reports of Karp-infected CD-1 models (i.p. and i.d. routes) and other *Ot* strains [29], as well as in Karp-infected B6 mice i.v. models [46,48,59]. Until the present, there were no detailed reports of CD-1 mouse infection via the i.v. route. We found that organ damage in CD-1 mice mimicked key pathological features of human disease in the lungs [62], including pulmonary edema and interstitial pneumonia with cellular infiltrates (Fig 3), some of which have been documented in previous reports of lethal infection in inbred mouse models [46,48,50,51]. Importantly, the pathological findings in Fig 3 of pulmonary alveolar edema were not evident in an i.d.-inoculated, self-limited B6 mouse model [27]. Liver samples showed perivascular lymphocyte infiltrates and vasculitis (S2 Fig), while kidney samples showed mild inflammation with multifocal mononuclear interstitial infiltrates (S3 Fig). These pathologic features have been reported in scrub typhus patients [54,55]. Like our previous reports on B6 lethal scrub typhus [48,50,63], the CD-1 lungs showed evidence of endothelial dysfunction (high *Ang2/Ang1* ratio, but low *Tie2*) and alveolar edema during the acute disease phase (Figs 3 and 4). This finding is important, as it reemphasizes that reduced Tie2 levels and elevated Ang1/Ang2 ratios are hallmarks of vascular dysfunction during severe stages of scrub typhus in outbred and inbred mouse strains, as well as in human patients [50]. Given its broader genetic diversity compared with inbred mouse strains, *Ot*-infected CD-1 mice may serve as an excellent model for scrub typhus immunology research.

Third, we have shown that lethally infected CD-1 mice exhibit similar type 1/CD8-skewed immune profiles as in lethally infected B6 mice [48,59] and BALB/C mice [45,47]. Our transcriptomic analysis revealed a Th1-skewed immune response during the acute phase of disease (Fig 5), which was further supported by significantly higher serum protein levels of IFNγ, G-CSF, IL-1α, and TNFα on day 10 (Fig 7). This finding was also surprising to us, as we expected a more balanced immune response due to the heterogenous nature of this mouse genetics. By providing characterization at the transcriptional and protein level of an infected outbred model, we have added additional credence to the prevalent inflammatory profile evident during lethal *Ot* infection, which helps dispel concerns that the strong type 1/weak type 2 profile is a trait solely of inbred murine models.

Our transcriptomic analysis has revealed several key biomarkers in the context of *Ot* infection. For example, the cytotoxicity gene *Gzmb* was highly upregulated at both days 8 and 12 (Fig 5). It is known that scrub typhus patients show high serum levels of granzymes A and B during the acute disease phase [64–68], which indicate increased cytotoxic activities and acute tissue injury [65–67]. Other cytotoxicity-associated genes revealed in our analysis (*Slamf7*, *Fasl*, and *Klrk1)* were also upregulated at days 8 and 12 (Tables 1 and 2). It is possible that local production and accumulation of these cytotoxic/effector molecules contribute to endothelial cell damage and vascular dysfunction. The significantly increased CCL3, CCL4, CCL5, and CCL11 protein levels in the sera imply a robust recruitment and activation of inflammatory monocytes and macrophages at late, but not early, stages of disease (Fig 7). The high levels of CXCL1/2/3 protein levels in sera imply neutrophil recruitment at late stages of disease, consistent with our findings for a pathological role of neutrophils in the lethal B6 model [46]. These findings collectively suggest a strong cytotoxic response, which is an important immune response required to combat cytosolic pathogens such as *Ot* bacteria. It is known that following *Ot* infection, CD8^+^ T cells are activated in humans [69], as well as inbred mice [31], as CD8^+^ T cells are essential for survival [70] and protection against *Ot* infection [31]. The role of multifunctional CD8^+^ T-cells in the CD-1 murine model was also recently investigated in an i.p. lethal *Ot* CD-1 murine model, in which an increase in TNF-producing populations was observed at day 14 pi (severe illness) [29]. However, the non-lethal *Ot* Woods strain i.p. and i.d. models had highly increased IL-2/TNF-producing populations by day 21 pi, when compared to the lethal model [29]. Our gene profiling studies support the development of a robust cytotoxic response to *Ot* infection and lay the framework for future studies to explore the host protective versus cellular damage roles of CD8^+^ T cells during sublethal and lethal scrub typhus.

Finally, our data from CD-1 mice have provided new evidence to define biomarkers of severe scrub typhus. Our Nanostring analysis revealed several previously unidentified markers. For example, *Mr1* was one of three significantly downregulated genes during the acute phase of disease (Fig 6A and 6D). This gene encodes for an antigen-presenting protein that presents metabolites of microbial vitamin B to mucosal-associated invariant T (MAIT) cells [71]. The suppression of *Mr1* by viral infection has been shown to inhibit bacterial driven MAIT TCR-activation [72]. It is known that the *Ot* lifecycle has certain viral-like features, like closely related rickettsial bacteria [73]. A recent study of scrub typhus patients demonstrated MAIT cell activation early in infection, but MAIT cell levels were diminished at later phases of disease course [74]. *Ot* may inhibit *Mr1*, disrupt activation of MAIT cells and/or impair innate-like responses. Given that the mouse and human *Mr1* genes are conserved, and that mouse MAIT cells closely resemble human counterparts [75], mouse models will be beneficial for *Ot* research through characterizing the physiological and pathological roles of *Mr1* gene expression in its related cells. Moreover, we found that *Il11ra1*and *Bcap31* genes were also significantly downregulated during the acute phase of disease (Tables 1 and 2). IL-11, a cytokine in the IL-6 family, has traditionally been known to have an anti-inflammatory role, regulating fibrosis and tissue damage. A recent study has shown that therapeutic targeting of IL-11 can reduce pulmonary fibrosis and inflammation, while another showed that blocking IL-11 signaling has anti-inflammatory effects, suggesting a complex role for IL-11 in sustained lung inflammation [76]. Given the downregulation of *Il11ra1* during infection (Fig 5A), it will be interesting to investigate whether enhanced expression of IL-11 and its related cytokines promote tissue repair. At day 12, *Bcap31* gene was suppressed (Fig 5B). B-cell receptor protein 31 (BCAP31) is a ubiquitously expressed transmembrane protein with a myriad of functions and is an important membrane transport protein for the endoplasmic reticulum [77]. The suppression of *Bcap31* may be relevant to *Ot* biology, as *Ot* is known to modulate the unfolded protein response pathway through the inhibition of the endoplasmic reticulum-associated degradation pathway [78], which results in the buildup of cellular stress. Importantly, *Ot* bacteria can take advantage of this stress for its stable growth in the cytosol [78]. Therefore, active suppression of *Bcap31* may be a potential mechanism for *Ot* to promote inflammation and immune dysregulation during its establishment and replication within the host cells.

Our study also revealed several genes whose expression levels were statistically significantly different by the unadjusted test statistic (*p* < 0.05). For example, we observed that Th2-associated markers were downregulated, including *Il-4*, *IL-5*, *Il-9*, *Il-13*, *Il-25*, *Ccrl1*, and *Ahr* (S4 Fig), like lethally infected B6 mice [52]. Whether such downregulation was due to the potent Th1-skewed proinflammatory responses, or to *Ot*-mediated active repression warrants further investigation.

While this is a pilot study mostly focusing on transcriptomic profiles in the lungs, it offers a broad view of the immune responses during severe scrub typhus. There are several caveats to this study, some of which will be addressed in future studies. For example, the increased sample size for CD-1 mice, especially at late/lethal stages of the disease, would greatly enhance the statistical power in our profiling analysis. The inclusion of protein-based analysis at the tissue level (immunofluorescent staining) and/or at the single-cell level (flow cytometry) would validate and expand the transcriptomic profiling data. While this study and our previous reports suggest that *Ot* infection drives a pro-inflammatory environment, mechanistic studies are needed to show how this bacterium modulates the immune response. The use of primary cell cultures would offer needed evidence to corroborate our findings of identified novel markers. Further studies will also be needed to reveal the molecular basis underlying mouse strain-specific differences in host susceptibility and immune responses following exposure to different *Ot* inoculation doses.

In summary, we have shown that CD-1 mice are more susceptible to *Ot* infection than B6 mice, showing pathologic features that mimic previous murine models and scrub typhus patients. Our results highlight the value of CD-1 mice as a good preclinical model of scrub typhus for defining host immune responses and dysregulation during severe infection. As the field of *Ot* research advances, the addition of an outbred murine model that mimics disease course, pathology, and immune response will be a vital tool for cost-effective studies to explore therapy and vaccines. Compared with inbred strains of mice, this outbred model provides a more genuine reflection of the genetically heterogeneous nature of the scrub typhus patient population. Our findings of Th1/CD8-skewed, but Th2-repressed, immunologic responses may help explain the increased susceptibility of CD-1 mice and pathogenesis of severe scrub typhus. This study will open new avenues for future mechanistic and translational studies by taking advantage of the genetic diversity in CD-1 mice to dissect the interplay between *Ot* and the host immune system.

## Supporting information

S1 Fig Lung bacterial burden in CD-1 mice following infection with *O*. *tsutsugamushi*. B6 and CD-1 mice were inoculated i.v. with *O*. *tsutsugamushi* Karp strain (5.6×10^4^ FFU) or PBS (mock). At indicated days post infection, organs were collected (3-4/group) for DNA extraction and bacterial burden analysis by qPCR. Data are presented as mean ± SEM. One-way ANOVA (non-parametric) was used for statistical analysis. ***, *p* < 0.001.
(TIFF)

S2 Fig Cellular infiltration and tissue injury in *O*. *tsutsugamushi-*infected CD-1 mice. CD-1 mice were infected as described in Fig 1. Liver tissues were collected at days 0 and 10 post-infection and subjected to hematoxylin and eosin staining. Perivascular lymphocytic inflitrates and vasculitis (asterisk) were observed in the liver tissue of infected mice. Scale bar 100 μm and 20 μm (zoomed).
(TIFF)

S3 Fig Cellular infiltration and tissue injury in *O*. *tsutsugamushi-*infected CD-1 mice. CD-1 mice were infected as described in Fig 1. Kidney tissues were collected at days 0 and 10 post-infection and subjected to hematoxylin and eosin staining. Foci of interstitial mononuclear inflammation (dagger) were observed in the cortex of the kidney of infected mice. Scale bar 100 μm and 20 μm (zoomed).
(TIFF)

S4 Fig Important biomarkers of immune dysregulation of CD-1 mice during acute infection. Lung transcriptomic profiling analyses were performed, as described in Fig 5. NanoString data show differentially increased or decreased gene expression (in Log2Fold change) of distinct cellular and immune responses at indicated days post-infection as compared to mock samples.
(TIFF)

S1 Table Primers used for bacterial and mRNA quantification via qPCR and RT-qPCR, respectively. (DOCX)

S2 Table Complete gene list from Nanostring analysis of CD-1 mouse lungs on Day 4. (DOCX)

S3 Table Complete gene list from Nanostring analysis of CD-1 mouse lungs on Day 8. (DOCX)

S4 Table Complete gene list from Nanostring analysis of CD-1 mouse lungs on Day 12. (DOCX)

## References

[1] Walker DH, Paris DH, Day NP, Shelite TR. Unresolved Problems Related to Scrub Typhus: A Seriously Neglected Life-Threatening Disease. Am J Trop Med Hyg. 2013;89: 301–307. doi: 10.4269/ajtmh.13-0064 23926142 PMC3741252

[2] Kelly DJ, Fuerst PA, Ching WM, Richards AL. Scrub typhus: the geographic distribution of phenotypic and genotypic variants of *Orientia tsutsugamushi*. Clin Infect Dis. 2009;48 Suppl 3. doi: 10.1086/596576 19220144

[3] Kim G, Ha NY, Min CK, Kim H il, Yen NTH, Lee KH, et al. Diversification of *Orientia tsutsugamushi* genotypes by intragenic recombination and their potential expansion in endemic areas. PLoS Negl Trop Dis. 2017. doi: 10.1371/journal.pntd.0005408 28248956 PMC5348041

[4] Walker DH. Scrub Typhus—Scientific Neglect, Ever-Widening Impact. N Engl J Med. 2016;375: 913–5. doi: 10.1056/NEJMp1608499 27602663

[5] Izzard L, Fuller A, Blacksell SD, Paris DH, Richards AL, Aukkanit N, et al. Isolation of a Novel *Orientia* Species (*O*. *chuto* sp. nov.) from a Patient Infected in Dubai. J Clin Microbiol. 2010;48: 4404–9. doi: 10.1128/JCM.01526-10 20926708 PMC3008486

[6] Weitzel T, Dittrich S, López J, Phuklia W, Martinez-Valdebenito C, Velásquez K, et al. Endemic Scrub Typhus in South America. N Engl J Med. 2016;375: 954–61. doi: 10.1056/NEJMoa1603657 27602667

[7] Kocher C, Jiang J, Morrison AC, Castillo R, Leguia M, Loyola S, et al. Serologic Evidence of Scrub Typhus in the Peruvian Amazon. Emerging Infectious Diseases. 2017;23: 1389–1391. doi: 10.3201/eid2308.170050 28726619 PMC5547797

[8] Balcells ME, Rabagliati R, García P, Poggi H, Oddó D, Concha M, et al. Endemic Scrub Typhus–like Illness, Chile. Emerg Infect Dis. 2011;17: 1659–63. doi: 10.3201/eid1709.100960 21888791 PMC3322051

[9] Thiga JW, Mutai BK, Eyako WK, Ng’Ang’A Z, Jiang J, Richards AL, et al. High Seroprevalence of Antibodies against Spotted Fever and Scrub Typhus Bacteria in Patients with Febrile Illness, Kenya. Emerging Infectious Diseases. 2015;21: 688–91. doi: 10.3201/eid2104.141387 25811219 PMC4378494

[10] Maina AN, Farris CM, Odhiambo A, Jiang J, Laktabai J, Armstrong J, et al. Q Fever, Scrub Typhus, and Rickettsial Diseases in Children, Kenya, 2011–2012. Emerging Infectious Diseases. 2016;22: 883–6. doi: 10.3201/eid2205.150953 27088502 PMC4861507

[11] Horton KC, Jiang J, Maina A, Dueger E, Zayed A, Ahmed AA, et al. Evidence of Rickettsia and Orientia Infections among Abattoir Workers in Djibouti. The American Journal of Tropical Medicine and Hygiene. 2016;95: 462–5. doi: 10.4269/ajtmh.15-0775 27273647 PMC4973201

[12] Rajapakse S, Rodrigo C, Fernando D. Scrub typhus: pathophysiology, clinical manifestations and prognosis. Asian Pac J Trop Med. 2012;5: 261–4. doi: 10.1016/S1995-7645(12)60036-4 22449515

[13] Varghese GM, Janardhanan J, Trowbridge P, Peter J v., Prakash JAJ, Sathyendra S, et al. Scrub typhus in South India: clinical and laboratory manifestations, genetic variability, and outcome. Int J Infect Dis. 2013;17: e981–7. doi: 10.1016/j.ijid.2013.05.017 23891643

[14] Hsu YH, Chen HI. Pulmonary pathology in patients associated with scrub typhus. Pathology. 2008;40: 268–271. doi: 10.1080/00313020801911488 18428046

[15] Wangrangsimakul T, Phuklia W, Newton PN, Richards AL, Day NPJ. Scrub Typhus and the Misconception of Doxycycline Resistance. Clin Infect Dis. 2020;70: 2444–9. doi: 10.1093/cid/ciz972 31570937 PMC7245148

[16] Watt G, Chouriyagune C, Ruangweerayud R, Watcharapichat P, Phulsuksombati D, Jongsakul K, et al. Scrub typhus infections poorly responsive to antibiotics in northern Thailand. The Lancet. 1996;348: 86–9. doi: 10.1016/s0140-6736(96)02501-9 8676722

[17] Rights FL, Smadel JE. Studies on scrub typhus; tsutsugamushi disease; heterogenicity of strains of *R*. *tsutsugamushi* as demonstrated by cross-vaccination studies. J Exp Med. 1948;87: 339–51. doi: 10.1084/JEM.87.4.339 18904219 PMC2135778

[18] Wd Kundin, Liu C, Harmon P, Rodina P. Pathogenesis of scrub typhus infection (*rickettsia tsutsugamushi*) as studied by immunofluorescence. J Immunol. 1964;93: 772–81. 14239936

[19] Lurchachaiwong W, McCardle W, Chan TC, Schuster AL, Richards AL. Development of an *Orientia tsutsugamushi* Lc-1 Murine Intraperitoneal Challenge Model for Scrub Typhus: Determination of Murine Lethal Dose (MuLD 50), Tissue Bacterial Loads, and Clinical Outcomes. Vector-Borne and Zoonotic Diseases. 2015;15: 539–544. doi: 10.1089/vbz.2015.1773 26378973

[20] Gharaibeh M, Hagedorn M, Lilla S, Hauptmann M, Heine H, Fleischer B, et al. Toll-Like Receptor 2 Recognizes *Orientia tsutsugamushi* and Increases Susceptibility to Murine Experimental Scrub Typhus. Infection and Immunity. 2016;84: 3379–87. doi: 10.1128/iai.00185-16 27620720 PMC5116716

[21] Jerrells TR, Eisemann CS. Role of T-lymphocytes in production of antibody to antigens of *Rickettsia tsutsugamushi* and other Rickettsia species. Infect Immun. 1983;41: 666–74. doi: 10.1128/IAI.41.2.666-674.1983 6223883 PMC264694

[22] Jerrells T, Osterman JV. Host defenses in experimental scrub typhus: delayed-type hypersensitivity responses of inbred mice. Infect Immun. 1982;35: 117–123. doi: 10.1128/iai.35.1.117-123.1982 6797944 PMC351004

[23] Seong SY, Huh MS, Jang WJ, Park SG, Kim JG, Woo SG, et al. Induction of homologous immune response to *Rickettsia tsutsugamushi* Boryong with a partial 56-kilodalton recombinant antigen fused with the maltose-binding protein MBP-Bor56. Infect Immun. 1997;65: 1541–1545. doi: 10.1128/IAI.65.4.1541-5.1997 9119501 PMC175167

[24] Murata M, Sudo K, Suzuki K, Aoyama Y, Nogami S, Tanaka H, et al. Proliferating sites of *Rickettsia tsutsugamushi* in mice by different routes of inoculation evidenced with immunofluorescence. Jpn J Exp Med. 1985;55: 193–9. 3938824

[25] Fukuhara M, Fukazawa M, Tamura A, Nakamura T, Urakami H. Survival of two *Orientia tsutsugamushi* bacterial strains that infect mouse macrophages with varying degrees of virulence. Microb Pathog. 2005;39: 177–87. doi: 10.1016/j.micpath.2005.08.004 16165341

[26] Kock F, Hauptmann M, Osterloh A, Schäberle TF, Poppert S, Frickmann H, et al. *Orientia tsutsugamushi* Is Highly Susceptible to the RNA Polymerase Switch Region Inhibitor Corallopyronin A In Vitro and In Vivo. Antimicrobial Agents and Chemotherapy. 2018;62. doi: 10.1128/AAC.01732-17 29358295 PMC5913972

[27] Soong L, Mendell NL, Olano JP, Rockx-Brouwer D, Xu G, Goez-Rivillas Y, et al. An Intradermal Inoculation Mouse Model for Immunological Investigations of Acute Scrub Typhus and Persistent Infection. PLoS Negl Trop Dis. 2016;10. doi: 10.1371/journal.pntd.0004884 27479584 PMC4968841

[28] Mendell NL, Bouyer DH, Walker DH. Murine models of scrub typhus associated with host control of *Orientia tsutsugamushi* infection. PLoS Negl Trop Dis. 2017;11. doi: 10.1371/journal.pntd.0005453 28282373 PMC5362142

[29] Luce-Fedrow A, Chattopadhyay S, Chan TC, Pearson G, Patton JB, Richards AL. Comparison of lethal and nonlethal mouse models of *Orientia tsutsugamushi* infection reveals t-cell population-associated cytokine signatures correlated with lethality and protection. Tropical Medicine and Infectious Disease. 2021;6. doi: 10.3390/tropicalmed6030121 34287349 PMC8293330

[30] Lurchachaiwong W, Chan TC, Richards AL, McCardle W, Schuster AL. Establishment of *Orientia tsutsugamushi* Lc-1 (Rickettsiales: Rickettsiaceae) infection in ICR outbred mice (Rodentia: Muridae) by needle challenge. Journal of Medical Entomology. 2014;51: 658–660. doi: 10.1603/ME13025 24897859

[31] Hauptmann M, Kolbaum J, Lilla S, Wozniak D, Gharaibeh M, Fleischer B, et al. Protective and Pathogenic Roles of CD8+ T Lymphocytes in Murine *Orientia tsutsugamushi* Infection. PLoS Negl Trop Dis. 2016;10: e0004991. doi: 10.1371/journal.pntd.0004991 27606708 PMC5015871

[32] Martin MD, Danahy DB, Hartwig SM, Harty JT, Badovinac VP. Revealing the Complexity in CD8 T Cell Responses to Infection in Inbred C57B/6 versus Outbred Swiss Mice. Front Immunol. 2017;8. doi: 10.3389/fimmu.2017.01527 29213267 PMC5702636

[33] Carreras E, Velasco de Andrés M, Orta-Mascaró M, Simões IT, Català C, Zaragoza O, et al. Discordant susceptibility of inbred C57BL/6 versus outbred CD1 mice to experimental fungal sepsis. Cell Microbiol. 2019;21. doi: 10.1111/cmi.12995 30577088

[34] Sunagar R, Kumar S, Namjoshi P, Rosa SJ, Hazlett KRO, Gosselin EJ. Evaluation of an outbred mouse model for *Francisella tularensis* vaccine development and testing. PLoS One. 2018;13. doi: 10.1371/journal.pone.0207587 30533047 PMC6289435

[35] Borenshtein D, Nambiar PR, Groff EB, Fox JG, Schauer DB. Development of fatal colitis in FVB mice infected with *Citrobacter rodentium*. Infect Immun. 2007;75: 3271–3281. doi: 10.1128/iai.01810-06 17470543 PMC1932959

[36] Ilmonen P, Penn DJ, Damjanovich K, Clarke J, Lamborn D, Morrison L, et al. Experimental infection magnifies inbreeding depression in house mice. J Evol Biol. 2008;21: 834–841. doi: 10.1111/j.1420-9101.2008.01510.x 18312317

[37] Lutz CM, Linder CC, Davisson MT. Strains, Stocks and Mutant Mice. The Laboratory Mouse. 2012; 37–56. doi: 10.1016/B978-0-12-382008-2.00003-9

[38] Shirai A, Huxsoll DL, Dohany AL, Montrey RD, Werner RM, Gan E. Characterization of *Rickettsia Tsutsugamushi* Strains in Two Species of Naturally Infected, Laboratory-Reared Chiggers. The American Journal of Tropical Medicine and Hygiene. 1982;31: 395–402. doi: 10.4269/ajtmh.1982.31.395 6176132

[39] Lerdthusnee K, Jenkitkasemwong S, Insuan S, Leepitakrat W, Monkanna T, Khlaimanee N, et al. Comparison of Immune Response against *Orientia tsutsugamushi*, a Causative Agent of Scrub Typhus, in 4-Week-Old and 10-Week-Old Scrub Typhus-Infected Laboratory Mice Using Enzyme-Linked Immunosorbent Assay Technique. Ann N Y Acad Sci. 2006;1078: 607–612. doi: 10.1196/annals.1374.121 17114788

[40] Lurchachaiwong W, Monkanna T, Leepitakrat S, Ponlawat A, Sattabongkot J, Schuster AL, et al. Variable clinical responses of a scrub typhus outbred mouse model to feeding by *Orientia tsutsugamushi* infected mites. Exp Appl Acarol. 2012;58: 23–34. doi: 10.1007/S10493-012-9563-8 22527840

[41] Takhampunya R, Tippayachai B, Promsathaporn S, Leepitakrat S, Monkanna T, Schuster AL, et al. Characterization Based on the 56-Kda Type-Specific Antigen Gene of *Orientia tsutsugamushi* Genotypes Isolated from *Leptotrombidium* Mites and the Rodent Host Post-Infection. The American Journal of Tropical Medicine and Hygiene. 2014;90: 139. doi: 10.4269/ajtmh.13-0393 24297814 PMC3886410

[42] Chao CC, Ingram BO, Lurchachaiwong W, Ching WM. Metabolic characterization of serum from mice challenged with *Orientia tsutsugamushi*–infected mites. New Microbes and New Infections. 2018;23: 70. doi: 10.1016/j.nmni.2018.01.005 29692908 PMC5913361

[43] Sunyakumthorn P, Paris DH, Chan T-C, Jones M, Luce-Fedrow A, Chattopadhyay S, et al. An intradermal inoculation model of scrub typhus in Swiss CD-1 mice demonstrates more rapid dissemination of virulent strains of *Orientia tsutsugamushi*. PLoS One. 2013;8: e54570. doi: 10.1371/journal.pone.0054570 23342173 PMC3546997

[44] Xu G, Chattopadhyay S, Jiang J, Chan TC, Chao CC, Ching WM, et al. Short- and long-term immune responses of CD-1 outbred mice to the scrub typhus DNA vaccine candidate: p47Kp. Ann N Y Acad Sci. 2005;1063: 266–269. doi: 10.1196/annals.1355.043 16481526

[45] Keller CA, Hauptmann M, Kolbaum J, Gharaibeh M, Neumann M, Glatzel M, et al. Dissemination of *Orientia tsutsugamushi* and inflammatory responses in a murine model of scrub typhus. PLoS Negl Trop Dis. 2014;8. doi: 10.1371/journal.pntd.0003064 25122501 PMC4133189

[46] Shelite TR, Saito TB, Mendell NL, Gong B, Xu G, Soong L, et al. Hematogenously disseminated *Orientia tsutsugamushi*-infected murine model of scrub typhus [corrected]. PLoS Negl Trop Dis. 2014;8. doi: 10.1371/journal.pntd.0002966 25010338 PMC4091938

[47] Yun J-H, Koh Y-S, Lee K-H, Hyun J-W, Choi Y-J, Jang W-J, et al. Chemokine and cytokine production in susceptible C3H/HeN mice and resistant BALB/c mice during *Orientia tsutsugamushi* infection. Microbiol Immunol. 2005;49: 551–7. doi: 10.1111/j.1348-0421.2005.tb03761.x 15965303

[48] Soong L, Wang H, Shelite TR, Liang Y, Mendell NL, Sun J, et al. Strong type 1, but impaired type 2, immune responses contribute to *Orientia tsutsugamushi*-induced pathology in mice. PLoS Negl Trop Dis. 2014;8. doi: 10.1371/journal.pntd.0003191 25254971 PMC4177881

[49] Soong L, Shelite TR, Xing Y, Kodakandla H, Liang Y, Trent BJ, et al. Type 1-skewed neuroinflammation and vascular damage associated with *Orientia tsutsugamushi* infection in mice. PLoS Negl Trop Dis. 2017;11: e0005765. doi: 10.1371/journal.pntd.0005765 28742087 PMC5542690

[50] Trent B, Liang Y, Xing Y, Esqueda M, Wei Y, Cho NH, et al. Polarized lung inflammation and Tie2/angiopoietin-mediated endothelial dysfunction during severe *Orientia tsutsugamushi* infection. PLoS Negl Trop Dis. 2020;14. doi: 10.1371/journal.pntd.0007675 32119672 PMC7067486

[51] Shelite TR, Liang Y, Wang H, Mendell NL, Trent BJ, Sun J, et al. IL-33-Dependent Endothelial Activation Contributes to Apoptosis and Renal Injury in *Orientia tsutsugamushi*-Infected Mice. PLoS Neglected Tropical Diseases. 2016;10. doi: 10.1371/journal.pntd.0004467 26943125 PMC4778942

[52] Fisher J, Card G, Liang Y, Trent B, Rosenzweig H, Soong L. *Orientia tsutsugamushi* selectively stimulates the C-type lectin receptor Mincle and type 1-skewed proinflammatory immune responses. PLoS Pathog. 2021;17. doi: 10.1371/journal.ppat.1009782 34320039 PMC8351992

[53] Moron CG, Popov VL, Feng HM, Wear D, Walker DH. Identification of the target cells of *Orientia tsutsugamushi* in human cases of scrub typhus. Mod Pathol. 2001;14: 752–759. doi: 10.1038/modpathol.3880385 11504834

[54] Allen AC, Spitz S. A Comparative Study of the Pathology of Scrub Typhus (Tsutsugamushi Disease) and Other Rickettsial Diseases. The American Journal of Pathology. 1945;21: 603. 19970829 PMC1934171

[55] Berman SJ, Kundin WD. Scrub typhus in South Vietnam. A study of 87 cases. Ann Intern Med. 1973;79: 26. doi: 10.7326/0003-4819-79-1-26 4198459

[56] Grommes J, Soehnlein O. Contribution of neutrophils to acute lung injury. Mol Med. 2011;17: 293–307. doi: 10.2119/molmed.2010.00138 21046059 PMC3060975

[57] Thurston G, Rudge JS, Ioffe E, Zhou H, Ross L, Croll SD, et al. Angiopoietin-1 protects the adult vasculature against plasma leakage. Nat Med. 2000;6: 460–463. doi: 10.1038/74725 10742156

[58] Fiedler U, Scharpfenecker M, Koidl S, Hegen A, Grunow V, Schmidt JM, et al. The Tie-2 ligand angiopoietin-2 is stored in and rapidly released upon stimulation from endothelial cell Weibel-Palade bodies. Blood. 2004;103: 4150–4156. doi: 10.1182/blood-2003-10-3685 14976056

[59] Soong L, Shelite HTR, Xing Y, Kodakandla H, Liang Y, Trent BJ, et al. Type 1-skewed neuroinflammation and vascular damage associated with *Orientia tsutsugamushi* infection in mice. PLoS Neglected Tropical Diseases. 2017;11. doi: 10.1371/journal.pntd.0005765 28742087 PMC5542690

[60] Seong SY, Choi MS, Kim IS. *Orientia tsutsugamushi* infection: overview and immune responses. Microbes Infect. 2001;3: 11–21. doi: 10.1016/s1286-4579(00)01352-6 11226850

[61] Nikodemova M, Watters JJ. Outbred ICR/CD1 mice display more severe neuroinflammation mediated by microglial TLR4/CD14 activation than inbred C57Bl/6 mice. Neuroscience. 2011;190: 67–74. doi: 10.1016/j.neuroscience.2011.06.006 21683771 PMC3156380

[62] Yeon JJ, Kim S, Yeh DW, Jun WL, Kim K il, Sun HL. Scrub typhus: clinical, pathologic, and imaging findings. Radiographics. 2007;27: 161–172. doi: 10.1148/rg.271065074 17235005

[63] Shelite TR, Liang Y, Wang H, Mendell NL, Trent BJ, Sun J, et al. IL-33-Dependent Endothelial Activation Contributes to Apoptosis and Renal Injury in *Orientia tsutsugamushi*-Infected Mice. Small PLC, editor. PLoS Negl Trop Dis. 2016;10: e0004467. doi: 10.1371/journal.pntd.0004467 26943125 PMC4778942

[64] de Fost M, Chierakul W, Pimda K, Dondorp AM, White NJ, van der Poll T. Activation of cytotoxic lymphocytes in patients with scrub typhus. The American Journal of Tropical Medicine and Hygiene. 2005;72: 465–467. doi: 10.4269/ajtmh.2005.72.465 15827287

[65] Chung DR, Lee YS, Lee SS. Kinetics of inflammatory cytokines in patients with scrub typhus receiving doxycycline treatment. J Infect. 2008;56: 44–50. doi: 10.1016/j.jinf.2007.09.009 17976731

[66] Kramme S, van An L, Nguyen DK, van Trin L, Tannich E, Rybniker J, et al. Orientia tsutsugamushi bacteremia and cytokine levels in Vietnamese scrub typhus patients. J Clin Microbiol. 2009;47: 586–589. doi: 10.1128/JCM.00997-08 19144812 PMC2650899

[67] Iwasaki H, Mizoguchi J, Takada N, Tai K, Ikegaya S, Ueda T. Correlation between the concentrations of tumor necrosis factor-alpha and the severity of disease in patients infected with *Orientia tsutsugamushi*. Int J Infect Dis. 2010;14. doi: 10.1016/J.IJID.2009.06.002 19699129

[68] Hwang J-H, Hwang J-H, Lee C-S. Elevated Extracellular Levels of Granzymes in Patients with Scrub Typhus. Am J Trop Med Hyg. 2021;105: 1680–1683. doi: 10.4269/ajtmh.20-1369 34634775 PMC8641366

[69] Cho BA, Ko Y, Kim YS, Kim S, Choi MS, Kim IS, et al. Phenotypic characterization of peripheral T cells and their dynamics in scrub typhus patients. PLoS Negl Trop Dis. 2012;6. doi: 10.1371/journal.pntd.0001789 22905277 PMC3419201

[70] Xu G, Mendell NL, Liang Y, Shelite TR, Goez-Rivillas Y, Soong L, et al. CD8+ T cells provide immune protection against murine disseminated endotheliotropic *Orientia tsutsugamushi* infection. 2017;11: e0005763. doi: 10.1371/journal.pntd.0005763 28723951 PMC5536391

[71] Kjer-Nielsen L, Patel O, Corbett AJ, le Nours J, Meehan B, Liu L, et al. MR1 presents microbial vitamin B metabolites to MAIT cells. Nature. 2012;491: 717–723. doi: 10.1038/nature11605 23051753

[72] McSharry BP, Samer C, McWilliam HEG, Ashley CL, Yee MB, Steain M, et al. Virus-Mediated Suppression of the Antigen Presentation Molecule MR1. Cell Rep. 2020;30: 2948–2962.e4. doi: 10.1016/j.celrep.2020.02.017 32130899 PMC7798347

[73] Uchiyama T. Tropism and pathogenicity of rickettsiae. Front Microbiol. 2012;3. doi: 10.3389/fmicb.2012.00230 22737150 PMC3382384

[74] Kang S-J, Jin H-M, Won EJ, Cho Y-N, Jung H-J, Kwon Y-S, et al. Activation, Impaired Tumor Necrosis Factor-α Production, and Deficiency of Circulating Mucosal-Associated Invariant T Cells in Patients with Scrub Typhus. 2016;10: e0004832. doi: 10.1371/journal.pntd.0004832 27463223 PMC4963088

[75] Rahimpour A, Koay HF, Enders A, Clanchy R, Eckle SBG, Meehan B, et al. Identification of phenotypically and functionally heterogeneous mouse mucosal-associated invariant T cells using MR1 tetramers. J Exp Med. 2015;212: 1095–1108. doi: 10.1084/jem.20142110 26101265 PMC4493408

[76] Ng B, Cook SA, Schafer S. Interleukin-11 signaling underlies fibrosis, parenchymal dysfunction, and chronic inflammation of the airway. Experimental & Molecular Medicine 2020 52:12. 2020;52: 1871–1878. doi: 10.1038/s12276-020-00531-5 33262481 PMC7705429

[77] Quistgaard EM. BAP31: Physiological functions and roles in disease. Biochimie. 2021;186: 105–129. doi: 10.1016/j.biochi.2021.04.008 33930507

[78] Rodino KG, VieBrock L, Evans SM, Ge H, Richards AL, Carlyon JA. *Orientia tsutsugamushi* Modulates Endoplasmic Reticulum-Associated Degradation To Benefit Its Growth. Infect Immun. 2017;86. doi: 10.1128/IAI.00596-17 29109174 PMC5736817

