# Correction: Host transcriptomic profiling of CD-1 outbred mice with severe clinical outcomes following infection with *Orientia tsutsugamushi*

**DOI:** 10.1371/journal.pntd.0011267

**Published:** 2023-04-06

**Authors:** Joseph Thiriot, Yuejin Liang, James Fisher, David H. Walker, Lynn Soong

The Funding statement is incorrect. The correct Funding statement should read:

This study was funded partly by NIAID R01 AI132674 (to LS), NIAID R21 AI156536 (to LS), NIAID R21 AI153586 (to YL), and NIAID T32 training grant AI007526-22 (to LS). JT and JF were partially supported by the T32 predoctoral fellowships. The funders had no role in study design, data collection and analysis, decision to publish, or preparation of the manuscript.
