# Supplementary figures and images for: Host transcriptomic profiling of CD-1 outbred mice with severe clinical outcomes following infection with Orientia tsutsugamushi
Source: PLoS Negl Trop Dis. 2022 Nov 23;16(11):e0010459. doi: 10.1371/journal.pntd.0010459 (PMC9683618; doi:10.1371/journal.pntd.0010459)

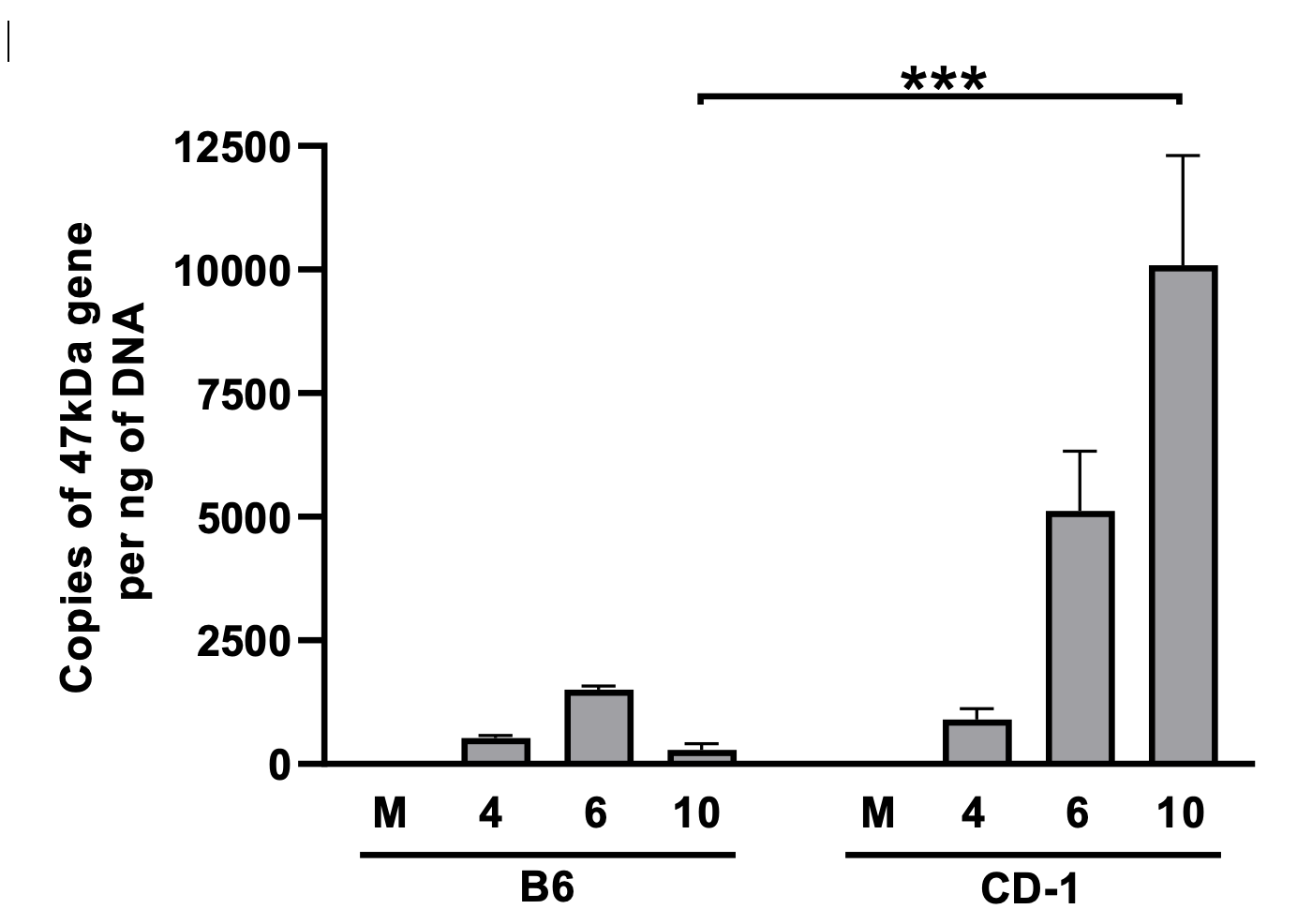

Supplement: S1 Fig — B6 and CD-1 mice were inoculated i.v. with O. tsutsugamushi Karp strain (5.6×10^4^ FFU) or PBS (mock). At indicated days post infection, organs were collected (3-4/group) for DNA extraction and bacterial burden analysis by qPCR. Data are presented as mean ± SEM. One-way ANOVA (non-parametric) was used for statistical analysis. ***, p < 0.001. (TIFF) [file pntd.0010459.s001.tiff]

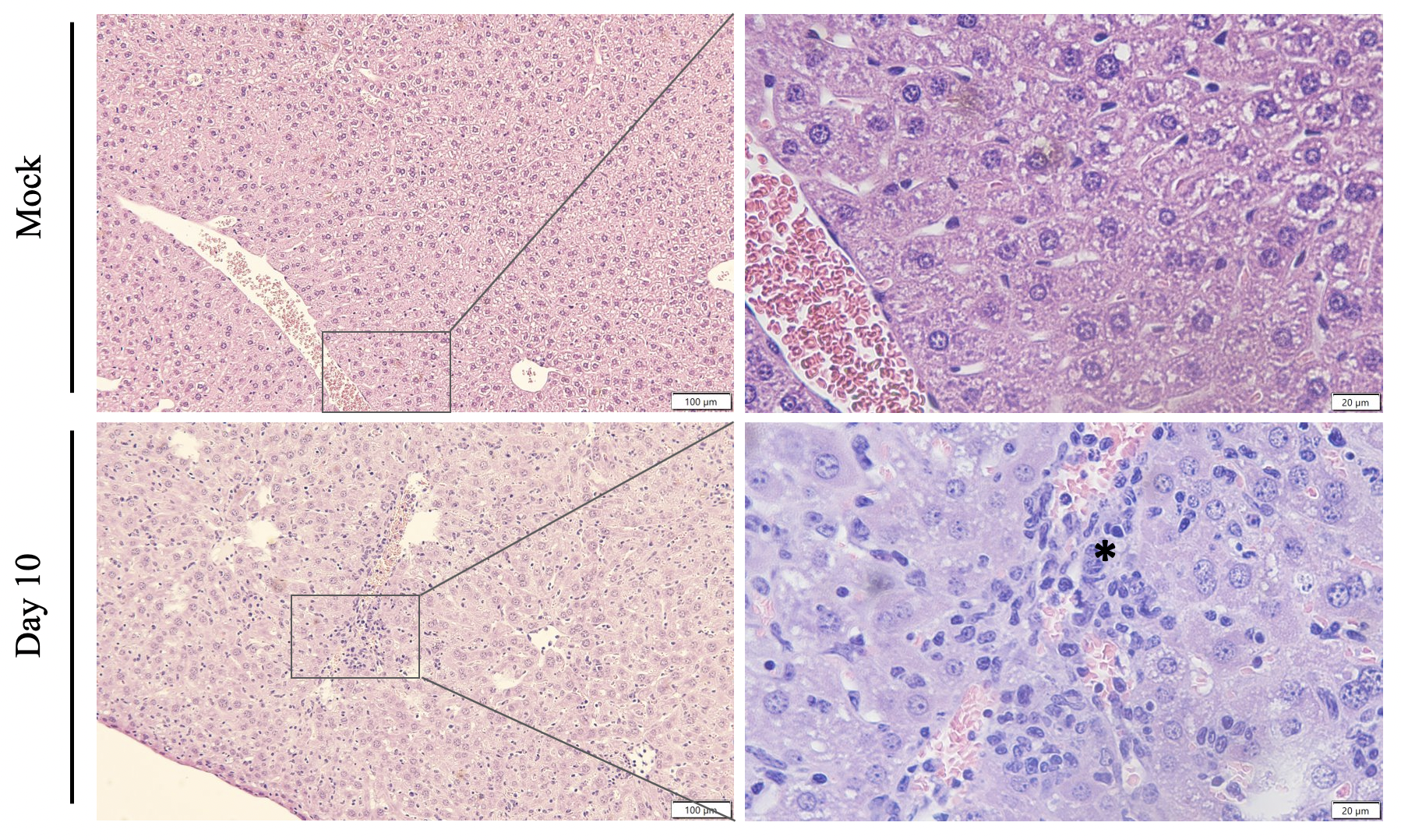

Supplement: S2 Fig — CD-1 mice were infected as described in Fig 1. Liver tissues were collected at days 0 and 10 post-infection and subjected to hematoxylin and eosin staining. Perivascular lymphocytic inflitrates and vasculitis (asterisk) were observed in the liver tissue of infected mice. Scale bar 100 μm and 20 μm (zoomed). (TIFF) [file pntd.0010459.s002.tiff]

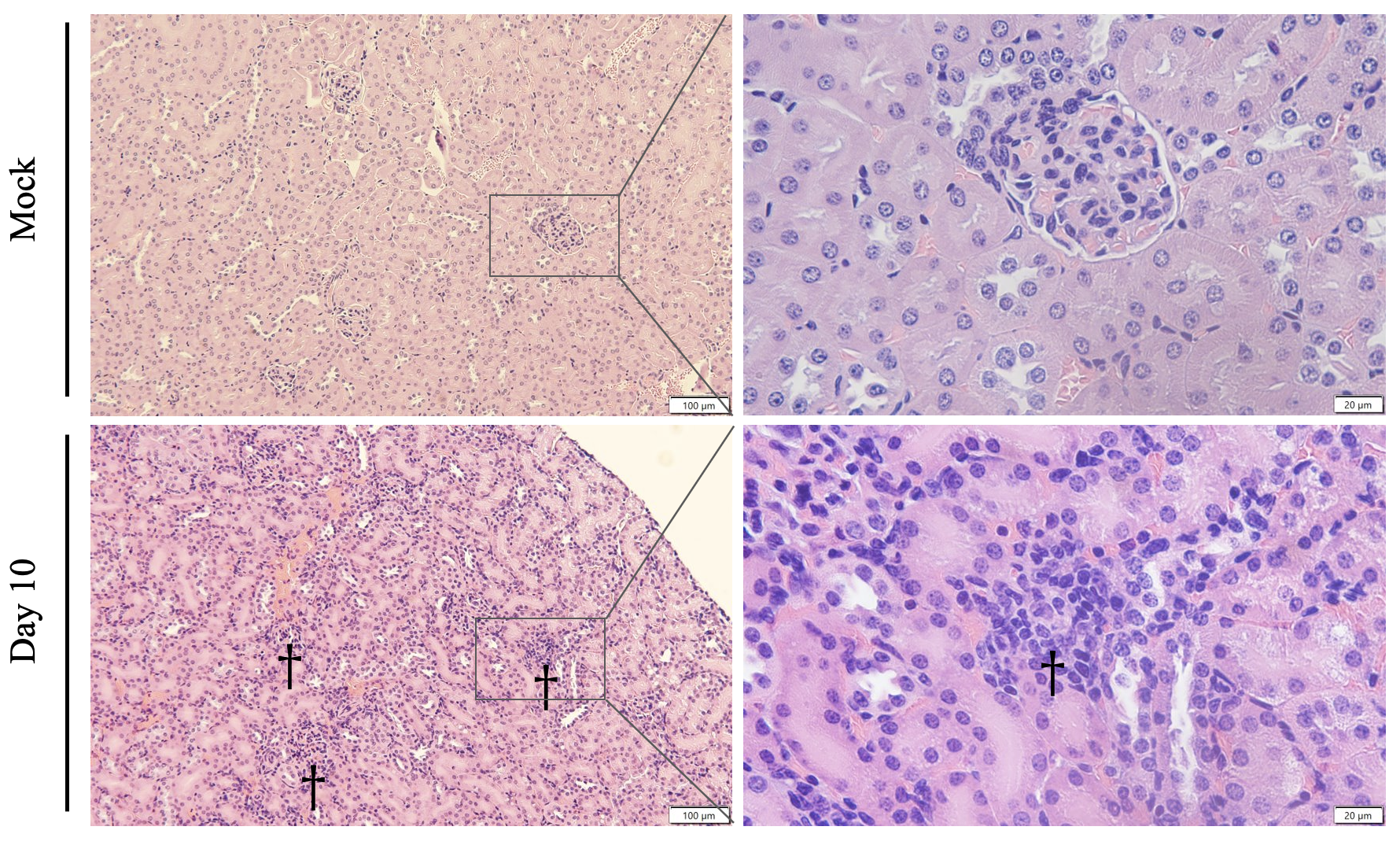

Supplement: S3 Fig — CD-1 mice were infected as described in Fig 1. Kidney tissues were collected at days 0 and 10 post-infection and subjected to hematoxylin and eosin staining. Foci of interstitial mononuclear inflammation (dagger) were observed in the cortex of the kidney of infected mice. Scale bar 100 μm and 20 μm (zoomed). (TIFF) [file pntd.0010459.s003.tiff]

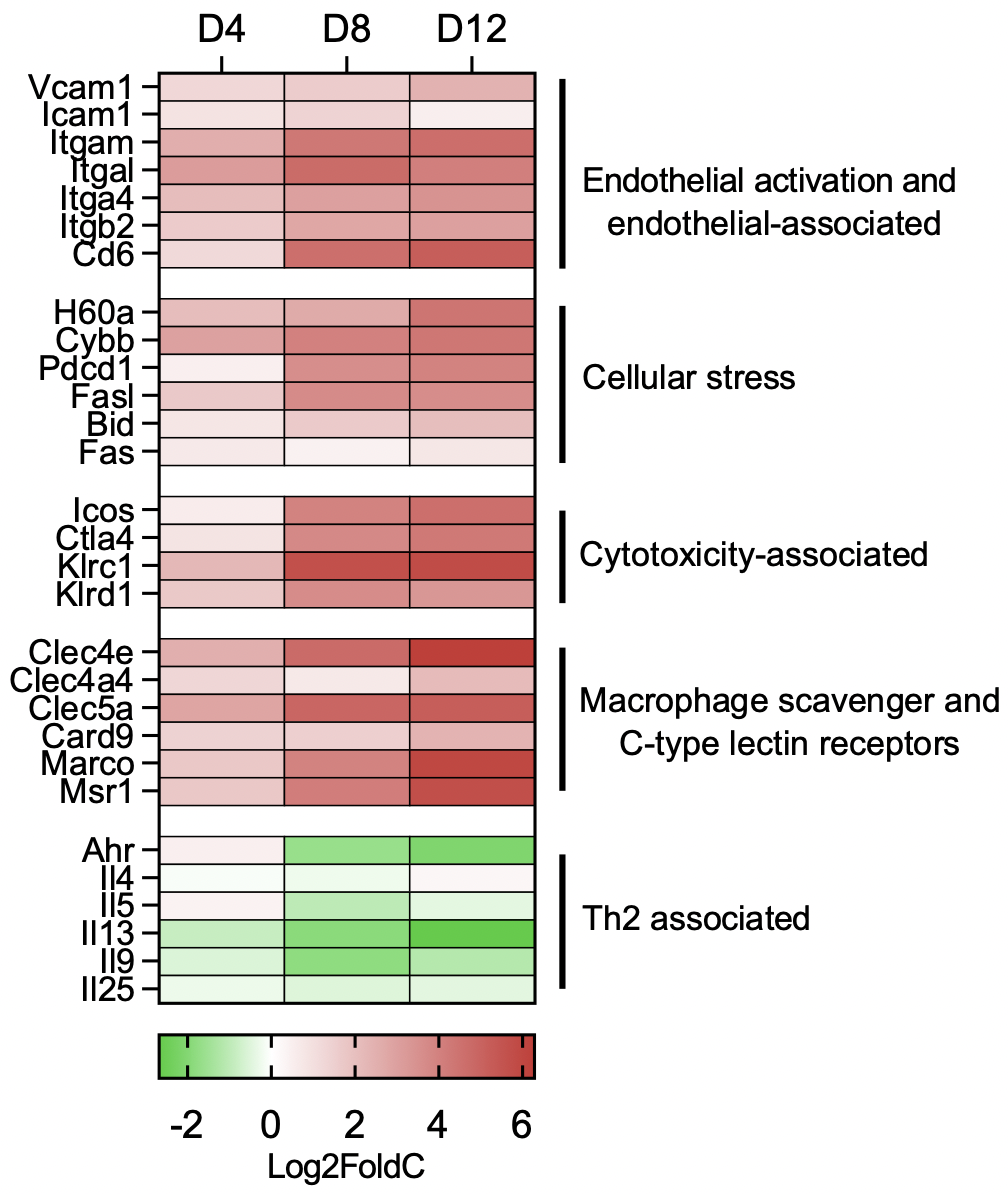

Supplement: S4 Fig — Lung transcriptomic profiling analyses were performed, as described in Fig 5. NanoString data show differentially increased or decreased gene expression (in Log2Fold change) of distinct cellular and immune responses at indicated days post-infection as compared to mock samples. (TIFF) [file pntd.0010459.s004.tiff]
