# Supplementary material for: Host transcriptomic profiling of CD-1 outbred mice with severe clinical outcomes following infection with Orientia tsutsugamushi
Source: PLoS Negl Trop Dis. 2022 Nov 23;16(11):e0010459. doi: 10.1371/journal.pntd.0010459 (PMC9683618; doi:10.1371/journal.pntd.0010459)
Supplement: S1 Table — (DOCX) [file pntd.0010459.s005.docx]

| Name | Orientation | Oligos |
| --- | --- | --- |
| *Otsu747* | Forward | TATGCCTGAGTAAGATACRTGAATRGAATT |
| *Otsu646* | Reverse | AACTGATTTTATTCAAACTAATGCTGCT |
| *Ang1* | Forward | CACATAGGGTGCAGCAACCA |
|  | Reverse | CGTCGTGTTCTGGAAGAATGA |
| *Ang2* | Forward | AGAATAAGCAAGTCTCGCTTCC |
|  | Reverse | TGAACCCTTTAGAGGCTCGGT |
| *Tie2* | Forward | CTGGAGGTTACTCAAGATGTGAC |
|  | Reverse | TCCGTATCCTTATAGCCTGTCC |
| *cxcl9* | Forward | TCC TTT TGG GCA TCA TCT TCC |
|  | Reverse | TTT GTA GTG GAT CGT GCC TCG |
| *cxcl10* | Forward | CCA AGT GCT GCC GTC ATT TTC |
|  | Reverse | GGC TCG CAG GGA TGA TTT CAA |
| *cxcl11* | Forward | GGCTTCCTTATGTTCAAACAGGG |
|  | Reverse | GCCGTTACTCGGGTAAATTACA |
| *ccl2* | Forward | TTAAAAACCTGGATCGGAACCAA |
|  | Reverse | GCATTAGCTTCAGATTTACGGGT |
| *IFNy* | Forward | AAC GCT ACA CAC TGC ATC TTG G |
|  | Reverse | GCC GTG GCA GTA ACA GCC |
| *TNFa* | Forward | CCC TCA CAC TCA GAT CAT CTT CT |
|  | Reverse | GCT ACG ACG TGG GCT ACA G |
| *IL-10* | Forward | GCTCTTACTGACTGGCATGAG |
|  | Reverse | CGCAGCTCTAGGAGCATGTG |
| *Irgm1* | Forward | AGACCCATTATGCTCCCCTGA |
|  | Reverse | CGGTGCTCCTACTGACCTCA |
| *Socs1* | Forward | CTGCGGCTTCTATTGGGGAC |
|  | Reverse | AAAAGGCAGTCGAAGGTCTCG |
| *Slamf7* | Forward | AGAACGCAGACTATGACACAATC |
|  | Reverse | AGGGAGCTGGGACTCTTTACC |
| *Klrk1* | Forward | GCACTAACTACCAGTCAACCTG |
|  | Reverse | CTCGAACAACGAACATTGGAGA |
| *Mr1* | Forward | CCTACCAGAGAATGATTGGCTG |
|  | Reverse | GCAACTCATGCAGGTTGGC |
| *Il1rl2* | Forward | GCAGCAGATACGTGTGAGGAC |
|  | Reverse | GTACCATGTCAGATTTACTGCCC |
| *Bcap31* | Forward | GCCACCTTCCTCTACGCAG |
|  | Reverse | TGCCATAGGTCACTACCAACTC |
| *Traf4* | Forward | CCCGGCTTCGACTACAAGTTC |
|  | Reverse | TCAGGGCATTTGAAGACTCCT |
| *Ahr* | Forward | AGCCGGTGCAGAAAACAGTAA |
|  | Reverse | AGGCGGTCTAACTCTGTGTTC |
