# Supplementary material for: Host transcriptomic profiling of CD-1 outbred mice with severe clinical outcomes following infection with Orientia tsutsugamushi
Source: PLoS Negl Trop Dis. 2022 Nov 23;16(11):e0010459. doi: 10.1371/journal.pntd.0010459 (PMC9683618; doi:10.1371/journal.pntd.0010459)
Supplement: S2 Table — (DOCX) [file pntd.0010459.s006.docx]

| **Supplemental Table 2. Complete list of differentially expressed genes in lung tissue (D4 vs. Mock)**  *, unadjusted test statistic; **, adjusted test statistic via the Benjamini-Yekutieli method | | | | |
| --- | --- | --- | --- | --- |
| **Gene** | **Log2 fold change** | **P-value*** | **BY P-value**** | **Probe ID** |
| Cxcl10 | 5.08 | 0.017658 | 1 | NM_021274.1 |
| Cd274 | 4.6 | 0.010418 | 1 | NM_021893.2 |
| Cxcl9 | 4.37 | 0.002343 | 1 | NM_008599.2 |
| Ifi204 | 4.03 | 0.031503 | 1 | NM_008329.2 |
| Irf7 | 3.8 | 0.038133 | 1 | NM_016850.2 |
| Tap1 | 3.67 | 0.002525 | 1 | NM_001161730.1 |
| Gzmb | 3.53 | 0.004842 | 1 | NM_013542.2 |
| Fcgr4 | 3.36 | 0.011003 | 1 | NM_144559.1 |
| Stat1 | 3.36 | 0.008782 | 1 | NM_009283.3 |
| Irgm1 | 3.28 | 0.015391 | 1 | NM_008326.1 |
| Il27 | 3.22 | 0.031857 | 1 | NM_145636.1 |
| S100a9 | 3.22 | 0.100049 | 1 | NM_009114.2 |
| Stat2 | 3.13 | 0.027576 | 1 | NM_019963.1 |
| Itgal | 3.11 | 0.03743 | 1 | NM_008400.2 |
| Gzma | 3 | 0.00638 | 1 | NM_010370.2 |
| Cybb | 2.95 | 0.08011 | 1 | NM_007807.2 |
| Lilrb4 | 2.89 | 0.08224 | 1 | NM_013532.2 |
| Clec5a | 2.83 | 0.024151 | 1 | NM_001038604.1 |
| Ccl5 | 2.76 | 0.041651 | 1 | NM_013653.1 |
| Lair1 | 2.66 | 0.035644 | 1 | NM_001113474.1 |
| S100a8 | 2.65 | 0.097773 | 1 | NM_013650.2 |
| Il1rn | 2.63 | 0.036782 | 1 | NM_031167.5 |
| Psmb9 | 2.61 | 0.011909 | 1 | NM_013585.2 |
| Ciita | 2.6 | 0.008542 | 1 | NM_007575.2 |
| Bst2 | 2.57 | 0.057156 | 1 | NM_198095.2 |
| Cxcr2 | 2.57 | 0.065735 | 1 | NM_009909.3 |
| Klrk1 | 2.56 | 0.008061 | 1 | NM_001083322.1 |
| Itgam | 2.53 | 0.031069 | 1 | NM_001082960.1 |
| Ifit2 | 2.51 | 0.061675 | 1 | NM_008332.2 |
| Clec4e | 2.5 | 0.040615 | 1 | NM_019948.2 |
| Prf1 | 2.48 | 0.002376 | 1 | NM_011073.2 |
| Klra7 | 2.47 | 0.030494 | 1 | NM_001110323.1 |
| Ctss | 2.45 | 0.094278 | 1 | NM_021281.2 |
| Tnfsf10 | 2.44 | 0.059267 | 1 | NM_009425.2 |
| Cxcl11 | 2.37 | 0.000548 | 1 | NM_019494.1 |
| Il1b | 2.36 | 0.029516 | 1 | NM_008361.3 |
| Ccl8 | 2.29 | 0.092496 | 1 | NM_021443.2 |
| Fcgr1 | 2.24 | 0.001277 | 1 | NM_010186.5 |
| B2m | 2.23 | 0.022981 | 1 | NM_009735.3 |
| Fcer1g | 2.23 | 0.037548 | 1 | NM_010185.4 |
| Klrc1 | 2.23 | 0.061128 | 1 | NM_001136068.1 |
| Il12rb2 | 2.21 | 0.094998 | 1 | NM_008354.3 |
| Socs1 | 2.2 | 0.03228 | 1 | NM_009896.2 |
| Klra4 | 2.18 | 0.141483 | 1 | NM_010649.3 |
| Ccl2 | 2.12 | 0.047127 | 1 | NM_011333.3 |
| Fcgr3 | 2.12 | 0.035739 | 1 | NM_010188.5 |
| Irf1 | 2.11 | 0.01912 | 1 | NM_008390.1 |
| H60a | 2.08 | 0.15491 | 1 | NM_010400.2 |
| Pou2f2 | 2.08 | 0.095321 | 1 | NM_001163554.1 |
| Itga4 | 2.07 | 0.078415 | 1 | NM_010576.3 |
| H2-Ab1 | 2.03 | 0.020804 | 1 | NM_207105.2 |
| Ccl4 | 2.01 | 0.022854 | 1 | NM_013652.1 |
| Ceacam1 | 2.01 | 0.278347 | 1 | NM_001039185.1 |
| Cfb | 2.01 | 0.037138 | 1 | NM_008198.2 |
| Psmb10 | 2.01 | 0.007222 | 1 | NM_013640.3 |
| Lck | 2 | 0.027896 | 1 | NM_010693.2 |
| Csf3r | 1.94 | 0.060328 | 1 | NM_001252651.1 |
| Lilrb3 | 1.94 | 0.012318 | 1 | NM_011095.2 |
| Il12rb1 | 1.92 | 0.277449 | 1 | NM_008353.2 |
| Tbx21 | 1.92 | 0.002229 | 1 | NM_019507.1 |
| Cd74 | 1.91 | 0.032016 | 1 | NM_001042605.1 |
| Ncf4 | 1.91 | 0.068948 | 1 | NM_008677.2 |
| H2-DMb2 | 1.89 | 0.053293 | 1 | NM_010388.4 |
| Trem1 | 1.89 | 0.145981 | 1 | NM_021406.3 |
| Il17ra | 1.85 | 0.049516 | 1 | NM_008359.1 |
| Ptprc | 1.85 | 0.097534 | 1 | NM_011210.3 |
| Spn | 1.84 | 0.050575 | 1 | NM_001037810.1 |
| Klra8 | 1.83 | 0.042651 | 1 | NM_010650.3 |
| H2-K1 | 1.82 | 0.11863 | 1 | NM_001001892.2 |
| Emr1 | 1.81 | 0.086559 | 1 | NM_010130.1 |
| Csf1 | 1.8 | 0.051057 | 1 | NM_001113530.1 |
| Ms4a1 | 1.77 | 0.308734 | 1 | NM_007641.5 |
| H2-Aa | 1.76 | 0.086191 | 1 | NM_010378.2 |
| Marco | 1.74 | 0.044438 | 1 | NM_010766.2 |
| Msr1 | 1.74 | 0.017388 | 1 | NM_001113326.1 |
| Sh2d1a | 1.74 | 0.023351 | 1 | NM_011364.3 |
| Camp | 1.71 | 0.493171 | 1 | NM_009921.2 |
| Ccr5 | 1.71 | 0.008097 | 1 | NM_009917.5 |
| Klrd1 | 1.71 | 0.032173 | 1 | NM_010654.2 |
| Il2rb | 1.7 | 0.027228 | 1 | NM_008368.3 |
| Arhgdib | 1.69 | 0.056246 | 1 | NM_007486.4 |
| Ifih1 | 1.68 | 0.076338 | 1 | NM_027835.2 |
| Nod2 | 1.68 | 0.045324 | 1 | NM_145857.2 |
| Ccrl2 | 1.67 | 0.070602 | 1 | NM_017466.4 |
| Irf5 | 1.67 | 0.044035 | 1 | NM_012057.3 |
| Bst1 | 1.66 | 0.047812 | 1 | NM_009763.3 |
| Fasl | 1.66 | 0.005103 | 1 | NM_010177.3 |
| Tagap | 1.66 | 0.04986 | 1 | NM_145968.2 |
| Il10ra | 1.65 | 0.021055 | 1 | NM_008348.2 |
| Ly86 | 1.64 | 0.076291 | 1 | NM_010745.2 |
| Ddx58 | 1.63 | 0.051951 | 1 | NM_172689.3 |
| Il1r2 | 1.62 | 0.009285 | 1 | NM_010555.4 |
| Itgb2 | 1.62 | 0.036529 | 1 | NM_008404.4 |
| Il2rg | 1.6 | 0.047933 | 1 | NM_013563.3 |
| Casp1 | 1.58 | 0.030062 | 1 | NM_009807.2 |
| Irf8 | 1.55 | 0.022423 | 1 | NM_008320.3 |
| Ptpn6 | 1.52 | 0.012885 | 1 | NM_013545.2 |
| H2-DMa | 1.51 | 0.011368 | 1 | NM_010386.3 |
| Sell | 1.51 | 0.010252 | 1 | NM_001164059.1 |
| Il18rap | 1.49 | 0.035525 | 1 | NM_010553.2 |
| Pml | 1.44 | 0.051596 | 1 | NM_008884.2 |
| Ltb | 1.43 | 0.183438 | 1 | NM_008518.2 |
| Nox1 | 1.43 | 0.198642 | 1 | NM_172203.1 |
| Card9 | 1.4 | 0.047728 | 1 | NM_001037747.1 |
| Cd19 | 1.38 | 0.387802 | 1 | NM_009844.2 |
| Il16 | 1.38 | 0.071509 | 1 | NM_010551.3 |
| Slamf7 | 1.38 | 0.016486 | 1 | NM_144539.5 |
| Kit | 1.37 | 0.157781 | 1 | NM_001122733.1 |
| Tapbp | 1.36 | 0.030164 | 1 | NM_009318.2 |
| Tlr1 | 1.34 | 0.122376 | 1 | NM_030682.1 |
| Ptpn22 | 1.32 | 0.052429 | 1 | NM_008979.1 |
| Tnfaip3 | 1.32 | 0.063439 | 1 | NM_009397.2 |
| Tnfrsf4 | 1.32 | 0.170559 | 1 | NM_011659.2 |
| Icam2 | 1.31 | 0.17156 | 1 | NM_010494.1 |
| Il21r | 1.3 | 0.084834 | 1 | NM_021887.1 |
| Ccl22 | 1.29 | 0.145586 | 1 | NM_009137.2 |
| Cd86 | 1.29 | 0.030289 | 1 | NM_019388.3 |
| Cfp | 1.27 | 0.078671 | 1 | NM_008823.3 |
| Clec4a4 | 1.26 | 0.026472 | 1 | NM_001005860.2 |
| Tgfb1 | 1.25 | 0.200277 | 1 | NM_011577.1 |
| Pecam1 | 1.24 | 0.222337 | 1 | NM_008816.2 |
| Cd1d1 | 1.23 | 0.159649 | 1 | NM_007639.3 |
| Tgfbi | 1.23 | 0.069918 | 1 | NM_009369.4 |
| Vcam1 | 1.23 | 0.01589 | 1 | NM_011693.2 |
| Lilra6 | 1.22 | 0.023039 | 1 | NM_011090.2 |
| Nox4 | 1.21 | 0.115391 | 1 | NM_015760.4 |
| Tnfrsf14 | 1.21 | 0.080096 | 1 | NM_178931.2 |
| Gata3 | 1.2 | 0.103741 | 1 | NM_008091.3 |
| Cd53 | 1.19 | 0.243463 | 1 | NM_007651.3 |
| Cd6 | 1.18 | 0.23917 | 1 | NM_001037801.2 |
| C2 | 1.17 | 0.070941 | 1 | NM_013484.2 |
| Prdm1 | 1.17 | 0.110031 | 1 | NM_007548.3 |
| C1qb | 1.16 | 0.09268 | 1 | NM_009777.2 |
| Tnf | 1.16 | 0.075856 | 1 | NM_013693.1 |
| Crlf2 | 1.15 | 0.167708 | 1 | NM_001164735.1 |
| Irak4 | 1.15 | 0.059463 | 1 | NM_029926.5 |
| Itgax | 1.15 | 0.217693 | 1 | NM_021334.2 |
| Csf2rb | 1.14 | 0.130584 | 1 | NM_007780.4 |
| Cdh5 | 1.13 | 0.324362 | 1 | NM_009868.3 |
| Ccl12 | 1.1 | 0.062274 | 1 | NM_011331.2 |
| Cd80 | 1.09 | 0.07899 | 1 | NM_009855.2 |
| Cxcl3 | 1.09 | 0.220335 | 1 | NM_203320.2 |
| Ly96 | 1.09 | 0.260054 | 1 | NM_016923.1 |
| Ccl7 | 1.08 | 0.226259 | 1 | NM_013654.2 |
| Tnfrsf1b | 1.08 | 0.118302 | 1 | NM_011610.3 |
| C1qa | 1.07 | 0.148874 | 1 | NM_007572.2 |
| Cd3e | 1.07 | 0.109744 | 1 | NM_007648.4 |
| Ptafr | 1.07 | 0.008917 | 1 | NM_001081211.1 |
| Il15ra | 1.06 | 0.053403 | 1 | NM_008358.2 |
| Jak3 | 1.06 | 0.087178 | 1 | NM_010589.5 |
| Cd3d | 1.05 | 0.182153 | 1 | NM_013487.2 |
| Plau | 1.05 | 0.136423 | 1 | NM_008873.2 |
| Tlr2 | 1.05 | 0.091778 | 1 | NM_011905.2 |
| Cd247 | 1.04 | 0.011169 | 1 | NM_001113391.2 |
| Nt5e | 1.04 | 0.196243 | 1 | NM_011851.3 |
| Ccl6 | 1.03 | 0.247888 | 1 | NM_009139.2 |
| Thy1 | 1.03 | 0.138784 | 1 | NM_009382.3 |
| Cx3cr1 | 1.02 | 0.077102 | 1 | NM_009987.3 |
| Pdcd1lg2 | 1.01 | 0.258674 | 1 | NM_021396.2 |
| Hc | 1 | 0.157987 | 1 | NM_010406.1 |
| Runx3 | 1 | 0.047438 | 1 | NM_019732.2 |
| Tlr8 | 1 | 0.224006 | 1 | NM_133212.2 |
| Csf1r | 0.99 | 0.044136 | 1 | NM_001037859.1 |
| Eomes | 0.99 | 0.012856 | 1 | NM_010136.2 |
| Il4ra | 0.98 | 0.257188 | 1 | NM_001008700.3 |
| Ccr2 | 0.97 | 0.369078 | 1 | NM_009915.2 |
| Sele | 0.97 | 0.100931 | 1 | NM_011345.2 |
| Ccr7 | 0.96 | 0.24675 | 1 | NM_007719.2 |
| Il15 | 0.96 | 0.187118 | 1 | NM_008357.2 |
| Aicda | 0.95 | 0.275597 | 1 | NM_009645.2 |
| Casp8 | 0.95 | 0.064368 | 1 | NM_009812.2 |
| Tnfsf13b | 0.93 | 0.100253 | 1 | NM_033622.1 |
| Btk | 0.92 | 0.191098 | 1 | NM_013482.2 |
| Prkcd | 0.91 | 0.028585 | 1 | NM_011103.2 |
| Tcf7 | 0.91 | 0.330505 | 1 | NM_009331.3 |
| Kir3dl1 | 0.9 | 0.162861 | 1 | NM_177749.3 |
| Nfkbiz | 0.9 | 0.026345 | 1 | NM_030612.1 |
| Pdgfb | 0.89 | 0.276947 | 1 | NM_011057.3 |
| Pdgfrb | 0.89 | 0.235773 | 1 | NM_008809.1 |
| Xcr1 | 0.89 | 0.156456 | 1 | NM_011798.4 |
| Ccrl1 | 0.88 | 0.325614 | 1 | NM_145700.2 |
| Cd79b | 0.88 | 0.371873 | 1 | NM_008339.2 |
| Cxcr1 | 0.88 | 0.218951 | 1 | NM_178241.4 |
| Icam1 | 0.86 | 0.366662 | 1 | NM_010493.2 |
| Ctla4 | 0.85 | 0.120832 | 1 | NM_009843.3 |
| Ikzf3 | 0.85 | 0.257252 | 1 | NM_011771.1 |
| Fyn | 0.84 | 0.150736 | 1 | NM_008054.2 |
| Cd59b | 0.83 | 0.256541 | 1 | NM_181858.1 |
| Csf2 | 0.83 | 0.26213 | 1 | NM_009969.4 |
| Ifngr2 | 0.83 | 0.146632 | 1 | NM_008338.3 |
| Tal1 | 0.82 | 0.389717 | 1 | NM_011527.2 |
| Cd69 | 0.81 | 0.424179 | 1 | NM_001033122.3 |
| Tnfsf15 | 0.8 | 0.110128 | 1 | NM_177371.3 |
| C7 | 0.79 | 0.258122 | 1 | XM_356827.6 |
| Ebi3 | 0.79 | 0.138577 | 1 | NM_015766.2 |
| Jak1 | 0.79 | 0.098265 | 1 | NM_146145.2 |
| G6pdx | 0.79 | 0.041098 | 1 | NM_008062.2 |
| Bid | 0.78 | 0.088876 | 1 | NM_007544.3 |
| C1ra | 0.78 | 0.103847 | 1 | NM_023143.3 |
| Map4k1 | 0.78 | 0.214997 | 1 | NM_008279.2 |
| Syk | 0.78 | 0.33566 | 1 | NM_011518.2 |
| Ccl3 | 0.77 | 0.026994 | 1 | NM_011337.1 |
| Cd2 | 0.77 | 0.304787 | 1 | NM_013486.2 |
| Batf | 0.75 | 0.076279 | 1 | NM_016767.2 |
| H2-Q10 | 0.75 | 0.390062 | 1 | NM_010391.4 |
| Ifnar2 | 0.75 | 0.066066 | 1 | NM_001110498.1 |
| Ets1 | 0.74 | 0.280454 | 1 | NM_001038642.1 |
| Hfe | 0.74 | 0.150267 | 1 | NM_010424.4 |
| Klra21 | 0.74 | 0.291941 | 1 | NM_053151.1 |
| Tlr3 | 0.74 | 0.09782 | 1 | NM_126166.2 |
| Tbk1 | 0.73 | 0.042773 | 1 | NM_019786.4 |
| Cd34 | 0.72 | 0.339679 | 1 | NM_001111059.1 |
| Cd48 | 0.72 | 0.108524 | 1 | NM_007649.4 |
| Relb | 0.72 | 0.088613 | 1 | NM_009046.2 |
| Zeb1 | 0.71 | 0.285164 | 1 | NM_011546.2 |
| Abcb1a | 0.7 | 0.350675 | 1 | NM_011076.1 |
| Ccl19 | 0.7 | 0.20175 | 1 | NM_011888.2 |
| Plaur | 0.7 | 0.215133 | 1 | NM_011113.3 |
| Tyk2 | 0.7 | 0.231169 | 1 | NM_018793.2 |
| Dpp4 | 0.69 | 0.227858 | 1 | NM_001159543.1 |
| Mme | 0.69 | 0.199249 | 1 | NM_008604.3 |
| Ccl9 | 0.68 | 0.170302 | 1 | NM_011338.2 |
| Folr4 | 0.68 | 0.327839 | 1 | NM_022888.2 |
| Ifi35 | 0.68 | 0.150325 | 1 | NM_027320.4 |
| Tyrobp | 0.68 | 0.218785 | 1 | NM_011662.2 |
| C4a | 0.67 | 0.208936 | 1 | NM_011413.2 |
| Casp3 | 0.67 | 0.052581 | 1 | NM_009810.2 |
| Ctsc | 0.66 | 0.106796 | 1 | NM_009982.2 |
| Ifng | 0.66 | 0.123357 | 1 | NM_008337.1 |
| Ikbke | 0.66 | 0.184535 | 1 | NM_019777.3 |
| Bcl3 | 0.65 | 0.182354 | 1 | NM_033601.3 |
| Fas | 0.65 | 0.057196 | 1 | NM_007987.2 |
| App | 0.64 | 0.231813 | 1 | NM_007471.2 |
| Cd40 | 0.64 | 0.308022 | 1 | NM_011611.2 |
| Nfkb1 | 0.64 | 0.110061 | 1 | NM_008689.2 |
| Il3 | 0.63 | 0.394663 | 1 | NM_010556.4 |
| Tcf4 | 0.63 | 0.057225 | 1 | NM_013685.1 |
| Tnfrsf9 | 0.63 | 0.304324 | 1 | NM_001077508.1 |
| Cxcr5 | 0.62 | 0.45775 | 1 | NM_007551.2 |
| Ifngr1 | 0.62 | 0.098519 | 1 | NM_010511.2 |
| Ikzf1 | 0.62 | 0.232264 | 1 | NM_001025597.1 |
| Ptger4 | 0.62 | 0.023509 | 1 | NM_008965.1 |
| Irak3 | 0.61 | 0.105441 | 1 | NM_028679.3 |
| Map4k4 | 0.61 | 0.325339 | 1 | NM_008696.2 |
| Gfi1 | 0.6 | 0.410962 | 1 | NM_010278.2 |
| Stat4 | 0.6 | 0.178462 | 1 | NM_011487.4 |
| Xcl1 | 0.6 | 0.498213 | 1 | NM_008510.1 |
| Cx3cl1 | 0.58 | 0.250286 | 1 | NM_009142.3 |
| Icos | 0.57 | 0.327503 | 1 | NM_017480.1 |
| Lilra5 | 0.57 | 0.355967 | 1 | NM_001081239.2 |
| Tgfbr1 | 0.57 | 0.220991 | 1 | NM_009370.2 |
| Klra5 | 0.56 | 0.47822 | 1 | NM_008463.2 |
| Lef1 | 0.56 | 0.280268 | 1 | NM_010703.3 |
| Mapk11 | 0.56 | 0.487191 | 1 | NM_011161.5 |
| Phlpp2 | 0.56 | 0.292497 | 1 | NM_001122594.2 |
| Foxp3 | 0.55 | 0.225688 | 1 | NM_054039.1 |
| Il27ra | 0.55 | 0.119244 | 1 | NM_016671.3 |
| Jak2 | 0.55 | 0.086568 | 1 | NM_001048177.1 |
| Nfatc2 | 0.55 | 0.00013 | 0.5 | NM_001037177.1 |
| Tnfrsf8 | 0.55 | 0.362257 | 1 | NM_009401.2 |
| Entpd1 | 0.54 | 0.414788 | 1 | NM_009848.3 |
| Gpr183 | 0.54 | 0.19491 | 1 | NM_183031.2 |
| Mapkapk2 | 0.54 | 0.00994 | 1 | NM_008551.1 |
| Nfkb2 | 0.54 | 0.031824 | 1 | NM_019408.2 |
| Cfi | 0.53 | 0.145978 | 1 | NM_007686.2 |
| Selplg | 0.53 | 0.479222 | 1 | NM_009151.3 |
| Tgfbr2 | 0.53 | 0.141885 | 1 | NM_009371.2 |
| C1s | 0.52 | 0.285443 | 1 | NM_144938.2 |
| Fn1 | 0.52 | 0.52156 | 1 | NM_010233.1 |
| Gpr44 | 0.52 | 0.427312 | 1 | NM_009962.2 |
| Ilf3 | 0.52 | 0.106952 | 1 | NM_010561.2 |
| Mbl2 | 0.52 | 0.462376 | 1 | NM_010776.1 |
| Ccr9 | 0.51 | 0.278249 | 1 | NM_009913.6 |
| Tlr9 | 0.5 | 0.138385 | 1 | NM_031178.2 |
| Polr2a | 0.49 | 0.165495 | 1 | NM_009089.2 |
| Ahr | 0.48 | 0.169669 | 1 | NM_013464.4 |
| Cmklr1 | 0.48 | 0.107183 | 1 | NM_008153.3 |
| Trem2 | 0.48 | 0.434152 | 1 | NM_031254.2 |
| Cd8b1 | 0.47 | 0.484182 | 1 | NM_009858.2 |
| Prim1 | 0.47 | 0.110267 | 1 | NM_008921.2 |
| Cxcl12 | 0.46 | 0.54616 | 1 | NM_021704.3 |
| Il10 | 0.46 | 0.576296 | 1 | NM_010548.1 |
| Il17b | 0.46 | 0.672133 | 1 | NM_019508.1 |
| Mapk14 | 0.46 | 0.101439 | 1 | NM_011951.2 |
| Nfkbia | 0.46 | 0.1657 | 1 | NM_010907.2 |
| Pdcd1 | 0.46 | 0.570687 | 1 | NM_008798.1 |
| Serping1 | 0.46 | 0.288621 | 1 | NM_009776.3 |
| Nfatc1 | 0.45 | 0.43605 | 1 | NM_016791.4 |
| Tnfsf12 | 0.45 | 0.297275 | 1 | NM_011614.3 |
| Ifnar1 | 0.44 | 0.192503 | 1 | NM_010508.1 |
| Cd27 | 0.43 | 0.305615 | 1 | NM_001042564.1 |
| Stat5a | 0.43 | 0.054575 | 1 | NM_011488.2 |
| Traf1 | 0.43 | 0.147628 | 1 | NM_009421.3 |
| Abl1 | 0.42 | 0.230693 | 1 | NM_009594.3 |
| Ptk2 | 0.42 | 0.401003 | 1 | NM_007982.2 |
| Hif1a | 0.41 | 0.178807 | 1 | NM_010431.1 |
| Il18 | 0.4 | 0.548665 | 1 | NM_008360.1 |
| Tigit | 0.4 | 0.364207 | 1 | NM_001146325.1 |
| Myd88 | 0.39 | 0.264035 | 1 | NM_010851.2 |
| Tslp | 0.39 | 0.109416 | 1 | NM_021367.1 |
| Il6 | 0.38 | 0.545493 | 1 | NM_031168.1 |
| Cr2 | 0.37 | 0.760763 | 1 | NM_007758.2 |
| Icosl | 0.37 | 0.235482 | 1 | NM_015790.3 |
| Il5 | 0.37 | 0.034416 | 1 | NM_010558.1 |
| Ptgs2 | 0.37 | 0.560573 | 1 | NM_011198.3 |
| Tgfb2 | 0.37 | 0.544341 | 1 | NM_009367.1 |
| Cxcr4 | 0.36 | 0.345837 | 1 | NM_009911.3 |
| Il12b | 0.36 | 0.652443 | 1 | NM_008352.1 |
| Il1a | 0.36 | 0.664024 | 1 | NM_010554.4 |
| Cxcl1 | 0.35 | 0.494732 | 1 | NM_008176.1 |
| Cxcl15 | 0.35 | 0.294811 | 1 | NM_011339.2 |
| Il12a | 0.35 | 0.48395 | 1 | NM_008351.1 |
| Mx1 | 0.35 | 0.306828 | 1 | NM_010846.1 |
| Traf5 | 0.35 | 0.564856 | 1 | NM_011633.1 |
| Cd44 | 0.34 | 0.144073 | 1 | NM_009851.2 |
| Klrb1 | 0.34 | 0.591493 | 1 | NM_001099918.1 |
| Rela | 0.34 | 0.302926 | 1 | NM_009045.4 |
| Tlr4 | 0.34 | 0.306291 | 1 | NM_021297.2 |
| Il10rb | 0.33 | 0.302888 | 1 | NM_008349.5 |
| Itga5 | 0.33 | 0.49484 | 1 | NM_010577.3 |
| Pla2g2e | 0.33 | 0.463493 | 1 | NM_012044.2 |
| Ptpn2 | 0.33 | 0.302663 | 1 | NM_001127177.1 |
| Batf3 | 0.32 | 0.447384 | 1 | NM_030060.2 |
| Ccr10 | 0.32 | 0.743547 | 1 | NM_007721.4 |
| Kir3dl2 | 0.32 | 0.698973 | 1 | NM_177748.2 |
| Notch1 | 0.32 | 0.279207 | 1 | NM_008714.2 |
| Ski | 0.32 | 0.223 | 1 | NM_011385.2 |
| Stat5b | 0.32 | 0.397752 | 1 | NM_011489.3 |
| Atm | 0.31 | 0.426704 | 1 | NM_007499.1 |
| Cd4 | 0.31 | 0.482396 | 1 | NM_013488.2 |
| Gusb | 0.31 | 0.422054 | 1 | NM_010368.1 |
| Abcf1 | 0.3 | 0.145145 | 1 | NM_013854.1 |
| Cdkn1a | 0.3 | 0.397808 | 1 | NM_007669.4 |
| Il2ra | 0.3 | 0.567433 | 1 | NM_008367.2 |
| Cd244 | 0.29 | 0.589838 | 1 | NM_018729.2 |
| Vtn | 0.29 | 0.648598 | 1 | NM_011707.2 |
| Il2 | 0.28 | 0.825037 | 1 | NM_008366.2 |
| Il33 | 0.28 | 0.626603 | 1 | NM_133775.1 |
| Itgb1 | 0.28 | 0.464104 | 1 | NM_010578.1 |
| C8g | 0.27 | 0.341502 | 1 | NM_027062.1 |
| Cd81 | 0.27 | 0.186506 | 1 | NM_133655.2 |
| Il17rb | 0.27 | 0.279079 | 1 | NM_019583.3 |
| Stat3 | 0.27 | 0.34932 | 1 | NM_213659.2 |
| Cd36 | 0.26 | 0.61045 | 1 | NM_007643.3 |
| Il6st | 0.26 | 0.064095 | 1 | NM_010560.2 |
| Phlpp1 | 0.25 | 0.559944 | 1 | NM_133821.3 |
| Cebpb | 0.24 | 0.208794 | 1 | NM_009883.3 |
| Map4k2 | 0.24 | 0.502678 | 1 | NM_009006.2 |
| Defb14 | 0.22 | 0.760246 | 1 | NM_183026.2 |
| Psmb7 | 0.22 | 0.3426 | 1 | NM_011187.1 |
| Src | 0.22 | 0.436212 | 1 | NM_001025395.2 |
| Cxcr6 | 0.21 | 0.618752 | 1 | NM_030712.4 |
| Mapk1 | 0.21 | 0.126877 | 1 | NM_011949.3 |
| Cxcr3 | 0.2 | 0.683647 | 1 | NM_009910.2 |
| Gm10499 | 0.2 | 0.851984 | 1 | XM_003086920.1 |
| Ikbkb | 0.2 | 0.686457 | 1 | NM_010546.2 |
| Itga6 | 0.2 | 0.689807 | 1 | NM_008397.3 |
| Tnfsf14 | 0.2 | 0.660475 | 1 | NM_019418.2 |
| Bax | 0.18 | 0.521079 | 1 | NM_007527.3 |
| Cd7 | 0.18 | 0.550203 | 1 | NM_009854.1 |
| C1qbp | 0.17 | 0.302232 | 1 | NM_007573.2 |
| Fcgrt | 0.17 | 0.214052 | 1 | NM_010189.3 |
| Hlx | 0.17 | 0.79974 | 1 | NM_008250.2 |
| Traf6 | 0.17 | 0.480782 | 1 | NM_009424.2 |
| Cd97 | 0.16 | 0.773821 | 1 | NM_011925.1 |
| Cfh | 0.16 | 0.671494 | 1 | NM_009888.3 |
| Cish | 0.16 | 0.59683 | 1 | NM_009895.3 |
| Psmc2 | 0.16 | 0.45774 | 1 | NM_011188.3 |
| Atg16l1 | 0.15 | 0.454675 | 1 | NM_029846.3 |
| Ifnb1 | 0.15 | 0.809977 | 1 | NM_010510.1 |
| Irak1 | 0.15 | 0.529735 | 1 | NM_008363.2 |
| Tbp | 0.15 | 0.449737 | 1 | NM_013684.3 |
| Fcer1a | 0.12 | 0.773869 | 1 | NM_010184.1 |
| Litaf | 0.12 | 0.778494 | 1 | NM_019980.1 |
| Tnfsf18 | 0.12 | 0.797463 | 1 | NM_183391.3 |
| Ube2l3 | 0.12 | 0.33766 | 1 | NM_009456.2 |
| Hcst | 0.11 | 0.846999 | 1 | NM_011827.3 |
| Ltbr | 0.11 | 0.509368 | 1 | NM_010736.3 |
| Btla | 0.1 | 0.893673 | 1 | NM_177584.3 |
| C4bp | 0.1 | 0.923043 | 1 | NM_007576.3 |
| Ikbkap | 0.09 | 0.75304 | 1 | NM_026079.3 |
| Il17re | 0.09 | 0.717299 | 1 | NM_001034029.1 |
| Ltb4r1 | 0.08 | 0.849972 | 1 | NM_008519.2 |
| Stat6 | 0.08 | 0.66332 | 1 | NM_009284.2 |
| Tnfrsf11a | 0.08 | 0.777516 | 1 | NM_009399.3 |
| Eef1g | 0.08 | 0.59724 | 1 | NM_026007.4 |
| Cd82 | 0.07 | 0.6781 | 1 | NM_001271430.1 |
| Tollip | 0.07 | 0.786071 | 1 | NM_023764.3 |
| Bcl2 | 0.06 | 0.857591 | 1 | NM_009741.3 |
| Cd164 | 0.06 | 0.801975 | 1 | NM_016898.2 |
| Traf3 | 0.06 | 0.792533 | 1 | NM_001048206.1 |
| Ccr6 | 0.05 | 0.950593 | 1 | NM_001190333.1 |
| Irak2 | 0.05 | 0.800845 | 1 | NM_001113553.1 |
| Maf | 0.05 | 0.871965 | 1 | NM_001025577.2 |
| Pdcd2 | 0.05 | 0.80818 | 1 | NM_008799.2 |
| Cd83 | 0.03 | 0.959231 | 1 | NM_009856.2 |
| Klra6 | 0.03 | 0.919269 | 1 | NM_008464.2 |
| Xbp1 | 0.03 | 0.92433 | 1 | NM_013842.2 |
| Il18r1 | 0.02 | 0.938449 | 1 | NM_001161842.1 |
| Ppia | 0.02 | 0.859018 | 1 | NM_008907.1 |
| Cd14 | 0.01 | 0.956129 | 1 | NM_009841.3 |
| Npc1 | 0.01 | 0.970007 | 1 | NM_008720.2 |
| Pparg | 0.01 | 0.973432 | 1 | NM_011146.1 |
| Polr1b | 0.01 | 0.987667 | 1 | NM_009086.2 |
| Bcl6 | 0 | 0.987988 | 1 | NM_009744.3 |
| Ctnnb1 | 0 | 0.994372 | 1 | NM_007614.2 |
| Nfatc3 | 0 | 0.991499 | 1 | NM_010901.2 |
| Sigirr | 0 | 0.986328 | 1 | NM_023059.3 |
| Smad3 | 0 | 0.995591 | 1 | NM_016769.3 |
| Traf2 | 0 | 0.982285 | 1 | NM_009422.2 |
| Sdha | 0 | 0.980612 | 1 | NM_023281.1 |
| Blnk | -0.01 | 0.958105 | 1 | NM_008528.4 |
| Il22 | -0.01 | 0.993321 | 1 | NM_016971.1 |
| Klrc3 | -0.01 | 0.994146 | 1 | NM_021378.1 |
| Rag2 | -0.01 | 0.985828 | 1 | NM_009020.3 |
| Tnfrsf13b | -0.01 | 0.988836 | 1 | NM_021349.1 |
| Cd99 | -0.02 | 0.945021 | 1 | NM_025584.2 |
| Psmd7 | -0.02 | 0.918581 | 1 | NM_010817.2 |
| Tmem173 | -0.02 | 0.92592 | 1 | NM_028261.1 |
| Tnfsf11 | -0.02 | 0.946194 | 1 | NM_011613.3 |
| Tnfsf8 | -0.02 | 0.92472 | 1 | NM_009403.2 |
| Rpl19 | -0.02 | 0.886374 | 1 | NM_009078.2 |
| Cradd | -0.04 | 0.685538 | 1 | NM_009950.2 |
| Irf3 | -0.04 | 0.904309 | 1 | NM_016849.3 |
| Fadd | -0.05 | 0.855341 | 1 | NM_010175.5 |
| H2-Eb1 | -0.05 | 0.973232 | 1 | NM_010382.2 |
| Mbp | -0.05 | 0.81904 | 1 | NM_010777.3 |
| Psmb5 | -0.05 | 0.430086 | 1 | NM_011186.1 |
| Ccr3 | -0.06 | 0.932501 | 1 | NM_009914.4 |
| Rorc | -0.06 | 0.773466 | 1 | NM_011281.2 |
| Tirap | -0.07 | 0.730258 | 1 | NM_001177847.1 |
| Trp53 | -0.07 | 0.80929 | 1 | NM_011640.1 |
| Adal | -0.08 | 0.781583 | 1 | NM_029475.1 |
| Gapdh | -0.09 | 0.577828 | 1 | NM_001001303.1 |
| Oaz1 | -0.09 | 0.534579 | 1 | NM_008753.4 |
| Cd9 | -0.1 | 0.778546 | 1 | NM_007657.3 |
| Il4 | -0.11 | 0.913904 | 1 | NM_021283.1 |
| Tnfrsf17 | -0.11 | 0.882782 | 1 | NM_011608.1 |
| Cxcl13 | -0.12 | 0.901768 | 1 | NM_018866.2 |
| Fcgr2b | -0.12 | 0.760506 | 1 | NM_001077189.1 |
| Pax5 | -0.12 | 0.901446 | 1 | NM_008782.2 |
| Socs3 | -0.12 | 0.552454 | 1 | NM_007707.2 |
| Tubb5 | -0.12 | 0.792949 | 1 | NM_011655.4 |
| C3 | -0.13 | 0.545997 | 1 | NM_009778.2 |
| Cd22 | -0.13 | 0.870633 | 1 | NM_001043317.2 |
| Chuk | -0.15 | 0.18673 | 1 | NM_001162410.1 |
| Notch2 | -0.16 | 0.22423 | 1 | NM_010928.1 |
| Tgfb3 | -0.16 | 0.556187 | 1 | NM_009368.2 |
| Tnfrsf13c | -0.16 | 0.839413 | 1 | NM_028075.2 |
| Bcap31 | -0.17 | 0.215778 | 1 | NM_012060.4 |
| C8a | -0.18 | 0.47596 | 1 | NM_146148.1 |
| Igf2r | -0.18 | 0.067943 | 1 | NM_010515.1 |
| Abcb10 | -0.19 | 0.666519 | 1 | NM_019552.2 |
| Cd79a | -0.2 | 0.873919 | 1 | NM_007655.3 |
| Ctsg | -0.2 | 0.847789 | 1 | NM_007800.1 |
| Lif | -0.2 | 0.70217 | 1 | NM_008501.2 |
| Icam4 | -0.21 | 0.635883 | 1 | NM_023892.2 |
| Irf4 | -0.23 | 0.403783 | 1 | NM_013674.1 |
| Slamf1 | -0.23 | 0.728742 | 1 | NM_013730.4 |
| Smad5 | -0.23 | 0.481514 | 1 | NM_008541.2 |
| Zap70 | -0.23 | 0.641214 | 1 | NM_009539.2 |
| Rae1 | -0.24 | 0.368764 | 1 | NM_175112.5 |
| Casp2 | -0.25 | 0.58177 | 1 | NM_007610.1 |
| Il19 | -0.25 | 0.689222 | 1 | NM_001009940.1 |
| Ccl11 | -0.26 | 0.440871 | 1 | NM_011330.3 |
| Lta | -0.26 | 0.722827 | 1 | NM_010735.1 |
| Cul9 | -0.28 | 0.243581 | 1 | NM_001081335.2 |
| Ikzf2 | -0.28 | 0.375491 | 1 | NM_011770.4 |
| Il25 | -0.28 | 0.771082 | 1 | NM_080729.2 |
| Ikzf4 | -0.29 | 0.521853 | 1 | NM_011772.2 |
| Il23r | -0.3 | 0.088759 | 1 | NM_144548.1 |
| Mif | -0.3 | 0.248882 | 1 | NM_010798.2 |
| Gp1bb | -0.31 | 0.64569 | 1 | NM_010327.2 |
| Il1rap | -0.31 | 0.248319 | 1 | NM_134103.2 |
| Il7r | -0.31 | 0.432837 | 1 | NM_008372.3 |
| Il21 | -0.33 | 0.681919 | 1 | NM_021782.2 |
| Ccl25 | -0.35 | 0.2632 | 1 | NM_009138.3 |
| Cd5 | -0.35 | 0.61923 | 1 | NM_007650.3 |
| Nfil3 | -0.35 | 0.188666 | 1 | NM_017373.3 |
| Zbtb7b | -0.35 | 0.471545 | 1 | NM_009565.4 |
| Cd96 | -0.37 | 0.098128 | 1 | NM_032465.2 |
| Rag1 | -0.37 | 0.307906 | 1 | NM_009019.2 |
| Gpi1 | -0.39 | 0.383676 | 1 | NM_008155.3 |
| Muc1 | -0.39 | 0.160065 | 1 | NM_013605.1 |
| Il1rl1 | -0.4 | 0.26273 | 1 | NM_001025602.2 |
| Hprt | -0.4 | 0.101219 | 1 | NM_013556.2 |
| Nos2 | -0.41 | 0.459475 | 1 | NM_010927.3 |
| Cd226 | -0.42 | 0.485351 | 1 | NM_001039149.1 |
| Il11ra1 | -0.42 | 0.086716 | 1 | NM_010549.3 |
| Il1r1 | -0.42 | 0.071457 | 1 | NM_001123382.1 |
| Il17f | -0.45 | 0.225097 | 1 | NM_145856.2 |
| Il22ra2 | -0.45 | 0.531553 | 1 | NM_178258.5 |
| Aire | -0.46 | 0.634793 | 1 | NM_009646.1 |
| C8b | -0.46 | 0.633064 | 1 | NM_133882.2 |
| Ccbp2 | -0.46 | 0.28586 | 1 | NM_021609.3 |
| Ifitm1 | -0.46 | 0.032499 | 1 | NM_001112715.1 |
| Il1rl2 | -0.46 | 0.032221 | 1 | NM_133193.3 |
| Il23a | -0.46 | 0.386705 | 1 | NM_031252.1 |
| Klrc2 | -0.46 | 0.292991 | 1 | NM_001098669.1 |
| Icam5 | -0.47 | 0.449363 | 1 | NM_008319.2 |
| Runx1 | -0.47 | 0.068578 | 1 | NM_001111021.1 |
| Btnl2 | -0.48 | 0.60358 | 1 | NM_079835.2 |
| Itga2b | -0.48 | 0.615263 | 1 | NM_010575.2 |
| Lcp2 | -0.48 | 0.391715 | 1 | NM_010696.3 |
| Mr1 | -0.48 | 0.021431 | 1 | NM_008209.4 |
| Ikbkg | -0.49 | 0.088937 | 1 | NM_178590.2 |
| Pla2g2a | -0.51 | 0.337117 | 1 | NM_001082531.1 |
| Tfrc | -0.51 | 0.035158 | 1 | NM_011638.3 |
| Tlr5 | -0.53 | 0.099561 | 1 | NM_016928.2 |
| Frmpd4 | -0.54 | 0.458602 | 1 | NM_001033330.2 |
| Il28a | -0.54 | 0.165621 | 1 | NM_001024673.2 |
| Il6ra | -0.55 | 0.420699 | 1 | NM_010559.2 |
| Il9 | -0.55 | 0.653644 | 1 | NM_008373.1 |
| Itln1 | -0.55 | 0.685885 | 1 | NM_010584.3 |
| Clu | -0.56 | 0.217058 | 1 | NM_013492.2 |
| Il13ra1 | -0.56 | 0.082052 | 1 | NM_133990.4 |
| Cd8a | -0.58 | 0.439176 | 1 | NM_001081110.2 |
| Masp2 | -0.58 | 0.564857 | 1 | NM_010767.3 |
| Ifna1 | -0.59 | 0.539635 | 1 | NM_010502.2 |
| Il7 | -0.59 | 0.211252 | 1 | NM_008371.2 |
| C6 | -0.63 | 0.167414 | 1 | NM_016704.2 |
| Cd109 | -0.63 | 0.41645 | 1 | NM_153098.3 |
| Cd3eap | -0.64 | 0.260539 | 1 | NM_145822.2 |
| Cd40lg | -0.64 | 0.296138 | 1 | NM_011616.2 |
| Traf4 | -0.64 | 0.180778 | 1 | NM_009423.4 |
| Fkbp5 | -0.67 | 0.268192 | 1 | NM_010220.3 |
| Klra1 | -0.67 | 0.336734 | 1 | NM_016659.3 |
| Ltf | -0.67 | 0.60698 | 1 | NM_008522.3 |
| Nox3 | -0.67 | 0.19701 | 1 | NM_198958.2 |
| H2-Ob | -0.72 | 0.638542 | 1 | NM_010389.3 |
| Cd160 | -0.73 | 0.093732 | 1 | NM_001163496.1 |
| Cd46 | -0.73 | 0.361137 | 1 | NM_010778.3 |
| Fcamr | -0.74 | 0.311586 | 1 | NM_001170632.1 |
| Cd55 | -0.77 | 0.03611 | 1 | NM_010016.2 |
| Ltb4r2 | -0.78 | 0.14768 | 1 | NM_020490.2 |
| C9 | -0.79 | 0.325434 | 1 | NM_013485.1 |
| Ccl26 | -0.84 | 0.258167 | 1 | NM_001013412.2 |
| Psmb11 | -0.85 | 0.310045 | 1 | NM_175204.4 |
| Il13 | -0.87 | 0.376451 | 1 | NM_008355.2 |
| Tnfaip6 | -0.88 | 0.114453 | 1 | NM_009398.2 |
| Btnl1 | -0.89 | 0.267224 | 1 | NM_001111094.1 |
| Ccl24 | -0.89 | 0.087545 | 1 | NM_019577.4 |
| Il17a | -0.89 | 0.368039 | 1 | NM_010552.3 |
| Masp1 | -0.9 | 0.084052 | 1 | NM_008555.2 |
| Il20 | -0.93 | 0.364009 | 1 | NM_021380.1 |
| Defb1 | -0.94 | 0.233941 | 1 | NM_007843.3 |
| Cd28 | -0.96 | 0.266128 | 1 | NM_007642.4 |
| Pigr | -1.05 | 0.102526 | 1 | NM_011082.3 |
| Alas1 | -1.14 | 0.102475 | 1 | NM_020559.2 |
| Cd209g | -1.21 | 0.087971 | 1 | NM_027343.3 |
| Ppbp | -1.25 | 0.364783 | 1 | NM_023785.2 |
| Cd163 | -1.32 | 0.033453 | 1 | NM_053094.2 |
| Cd24a | -1.33 | 0.028755 | 1 | NM_009846.2 |
| Ifna2 | -1.35 | 0.228811 | 1 | NM_010503.2 |
| Ccr4 | -1.37 | 0.13148 | 1 | NM_009916.2 |
| Ccl20 | -1.38 | 0.203031 | 1 | NM_016960.1 |
| Ncam1 | -1.44 | 0.050232 | 1 | NM_001113204.1 |
| H2-Ea-ps | -1.48 | 0.770161 | 1 | NM_010381.2 |
| Hamp | -2.05 | 0.206138 | 1 | NM_032541.1 |
| Cfd | -2.8 | 0.045615 | 1 | NM_013459.1 |
| Ccr8 | -2.99 | 0.077269 | 1 | NM_007720.2 |
