# Supplementary material for: Host transcriptomic profiling of CD-1 outbred mice with severe clinical outcomes following infection with Orientia tsutsugamushi
Source: PLoS Negl Trop Dis. 2022 Nov 23;16(11):e0010459. doi: 10.1371/journal.pntd.0010459 (PMC9683618; doi:10.1371/journal.pntd.0010459)
Supplement: S3 Table — (DOCX) [file pntd.0010459.s007.docx]

| **Supplemental Table 3. Complete list of differentially expressed genes in lung tissue (D8 vs. Mock)**  *, unadjusted test statistic; **, adjusted test statistic via the Benjamini-Yekutieli method | | | | |
| --- | --- | --- | --- | --- |
| **Gene** | **Log2 fold change** | **P-value*** | **BY P-value**** | **Probe ID** |
| Gzmb | 7.57 | 7.98E-06 | 0.01 | NM_013542.2 |
| Cxcl9 | 7.48 | 5.64E-05 | 0.03 | NM_008599.2 |
| Cxcl10 | 7.47 | 0.001459 | 0.12 | NM_021274.1 |
| Ccl5 | 6.25 | 0.003633 | 0.18 | NM_013653.1 |
| S100a9 | 5.95 | 0.029146 | 0.52 | NM_009114.2 |
| Ccl4 | 5.91 | 0.000537 | 0.08 | NM_013652.1 |
| Ccl2 | 5.84 | 0.000618 | 0.08 | NM_011333.3 |
| Cxcl11 | 5.75 | 0.000636 | 0.08 | NM_019494.1 |
| Klrc1 | 5.64 | 0.00089 | 0.1 | NM_001136068.1 |
| Ifi204 | 5.62 | 0.021078 | 0.43 | NM_008329.2 |
| Ifng | 5.13 | 0.001425 | 0.12 | NM_008337.1 |
| Irf7 | 5.12 | 0.013799 | 0.36 | NM_016850.2 |
| Cd274 | 5.04 | 0.014681 | 0.36 | NM_021893.2 |
| Fcgr4 | 5 | 0.012314 | 0.35 | NM_144559.1 |
| Clec5a | 4.89 | 0.007842 | 0.27 | NM_001038604.1 |
| Lck | 4.84 | 0.002695 | 0.16 | NM_010693.2 |
| Il1r2 | 4.82 | 0.000276 | 0.05 | NM_010555.4 |
| Cd8b1 | 4.76 | 0.001127 | 0.11 | NM_009858.2 |
| Il1rn | 4.76 | 0.01714 | 0.38 | NM_031167.5 |
| Clec4e | 4.72 | 0.009465 | 0.3 | NM_019948.2 |
| Itgal | 4.7 | 0.02412 | 0.47 | NM_008400.2 |
| Gzma | 4.66 | 0.004018 | 0.19 | NM_010370.2 |
| Lilrb4 | 4.65 | 0.040166 | 0.63 | NM_013532.2 |
| S100a8 | 4.65 | 0.038046 | 0.61 | NM_013650.2 |
| Tbx21 | 4.62 | 0.000442 | 0.07 | NM_019507.1 |
| Cd6 | 4.58 | 0.016081 | 0.37 | NM_001037801.2 |
| Cd3e | 4.53 | 0.000161 | 0.05 | NM_007648.4 |
| Klrk1 | 4.48 | 0.000132 | 0.04 | NM_001083322.1 |
| Sh2d1a | 4.47 | 0.001881 | 0.14 | NM_011364.3 |
| Tap1 | 4.4 | 0.006377 | 0.24 | NM_001161730.1 |
| Cfb | 4.39 | 0.000271 | 0.05 | NM_008198.2 |
| Ccl7 | 4.36 | 0.000867 | 0.1 | NM_013654.2 |
| Cxcr2 | 4.34 | 0.027516 | 0.5 | NM_009909.3 |
| Il12rb1 | 4.31 | 0.079111 | 0.97 | NM_008353.2 |
| Itgam | 4.29 | 0.016283 | 0.37 | NM_001082960.1 |
| Socs1 | 4.29 | 0.000169 | 0.05 | NM_009896.2 |
| Il27 | 4.25 | 0.025473 | 0.48 | NM_145636.1 |
| Il12rb2 | 4.21 | 0.027421 | 0.5 | NM_008354.3 |
| Irgm1 | 4.21 | 1.27E-05 | 0.01 | NM_008326.1 |
| Thy1 | 4.17 | 0.001095 | 0.11 | NM_009382.3 |
| Msr1 | 4.12 | 0.00507 | 0.23 | NM_001113326.1 |
| Il18rap | 4.11 | 0.000911 | 0.1 | NM_010553.2 |
| Ccr5 | 4.03 | 0.000126 | 0.04 | NM_009917.5 |
| Cd3d | 4.03 | 0.001851 | 0.14 | NM_013487.2 |
| Cybb | 3.99 | 0.050473 | 0.72 | NM_007807.2 |
| Tigit | 3.97 | 0.003091 | 0.17 | NM_001146325.1 |
| Stat1 | 3.95 | 7.65E-05 | 0.04 | NM_009283.3 |
| Icos | 3.94 | 0.007576 | 0.27 | NM_017480.1 |
| Il21r | 3.94 | 0.00763 | 0.27 | NM_021887.1 |
| Cd8a | 3.93 | 0.003432 | 0.18 | NM_001081110.2 |
| Marco | 3.93 | 0.001432 | 0.12 | NM_010766.2 |
| Prf1 | 3.88 | 0.001977 | 0.14 | NM_011073.2 |
| Fcgr1 | 3.87 | 0.002362 | 0.16 | NM_010186.5 |
| Ifit2 | 3.86 | 0.000232 | 0.05 | NM_008332.2 |
| Bst1 | 3.8 | 0.004589 | 0.21 | NM_009763.3 |
| Tnf | 3.74 | 0.005738 | 0.24 | NM_013693.1 |
| Ctla4 | 3.73 | 0.000306 | 0.06 | NM_009843.3 |
| Slamf7 | 3.71 | 0.000059 | 0.03 | NM_144539.5 |
| Fasl | 3.66 | 0.000131 | 0.04 | NM_010177.3 |
| Klrd1 | 3.63 | 0.003093 | 0.17 | NM_010654.2 |
| Runx3 | 3.62 | 0.000431 | 0.07 | NM_019732.2 |
| Fcgr3 | 3.61 | 0.017152 | 0.38 | NM_010188.5 |
| Ptpn22 | 3.61 | 0.002592 | 0.16 | NM_008979.1 |
| Klra7 | 3.58 | 0.016167 | 0.37 | NM_001110323.1 |
| Pdcd1 | 3.58 | 0.000267 | 0.05 | NM_008798.1 |
| Trem1 | 3.57 | 0.049615 | 0.72 | NM_021406.3 |
| Cd247 | 3.55 | 0.000996 | 0.1 | NM_001113391.2 |
| Ccl8 | 3.49 | 0.027777 | 0.5 | NM_021443.2 |
| Ctss | 3.48 | 0.052778 | 0.74 | NM_021281.2 |
| Spn | 3.46 | 0.007507 | 0.27 | NM_001037810.1 |
| Stat2 | 3.45 | 0.022765 | 0.45 | NM_019963.1 |
| Ccl3 | 3.44 | 2.99E-06 | 0.01 | NM_011337.1 |
| Lilrb3 | 3.43 | 0.006269 | 0.24 | NM_011095.2 |
| Arhgdib | 3.32 | 0.013823 | 0.36 | NM_007486.4 |
| Klra8 | 3.31 | 0.010889 | 0.32 | NM_010650.3 |
| Camp | 3.23 | 0.10826 | 1 | NM_009921.2 |
| Irf1 | 3.19 | 0.005293 | 0.23 | NM_008390.1 |
| Klra4 | 3.19 | 0.066317 | 0.87 | NM_010649.3 |
| Cd69 | 3.12 | 0.053371 | 0.74 | NM_001033122.3 |
| Fcer1g | 3.12 | 0.022705 | 0.45 | NM_010185.4 |
| Psmb9 | 3.12 | 0.008828 | 0.29 | NM_013585.2 |
| Tagap | 3.12 | 0.010112 | 0.31 | NM_145968.2 |
| Pou2f2 | 3.11 | 0.053307 | 0.74 | NM_001163554.1 |
| Cxcr6 | 3.06 | 0.002171 | 0.15 | NM_030712.4 |
| Il21 | 3.04 | 0.035097 | 0.58 | NM_021782.2 |
| Emr1 | 2.99 | 0.036076 | 0.59 | NM_010130.1 |
| Casp1 | 2.98 | 0.01174 | 0.34 | NM_009807.2 |
| Itga4 | 2.98 | 0.027847 | 0.5 | NM_010576.3 |
| Tnfrsf9 | 2.95 | 0.001691 | 0.13 | NM_001077508.1 |
| Lair1 | 2.94 | 0.030048 | 0.53 | NM_001113474.1 |
| Ptprc | 2.91 | 0.041286 | 0.64 | NM_011210.3 |
| Ncf4 | 2.9 | 0.031722 | 0.55 | NM_008677.2 |
| Il1b | 2.88 | 0.018122 | 0.4 | NM_008361.3 |
| Bst2 | 2.87 | 0.011782 | 0.34 | NM_198095.2 |
| Csf3r | 2.81 | 0.032926 | 0.55 | NM_001252651.1 |
| Itgb2 | 2.76 | 0.013095 | 0.35 | NM_008404.4 |
| Ptpn6 | 2.75 | 0.002744 | 0.16 | NM_013545.2 |
| Il2ra | 2.71 | 0.018711 | 0.41 | NM_008367.2 |
| Cd53 | 2.68 | 0.064942 | 0.86 | NM_007651.3 |
| Tlr1 | 2.65 | 0.032987 | 0.55 | NM_030682.1 |
| H60a | 2.64 | 0.094283 | 1 | NM_010400.2 |
| Il17ra | 2.62 | 0.032646 | 0.55 | NM_008359.1 |
| Cd5 | 2.61 | 0.011683 | 0.34 | NM_007650.3 |
| Batf | 2.59 | 0.002255 | 0.15 | NM_016767.2 |
| Il2rb | 2.58 | 0.0135 | 0.36 | NM_008368.3 |
| Cfp | 2.56 | 0.020402 | 0.43 | NM_008823.3 |
| Cxcl13 | 2.55 | 0.088239 | 1 | NM_018866.2 |
| Ciita | 2.53 | 0.011851 | 0.34 | NM_007575.2 |
| H2-DMb2 | 2.53 | 0.041254 | 0.64 | NM_010388.4 |
| Ltb | 2.53 | 0.073744 | 0.92 | NM_008518.2 |
| Stat4 | 2.53 | 0.001366 | 0.12 | NM_011487.4 |
| Tnfrsf4 | 2.5 | 0.049798 | 0.72 | NM_011659.2 |
| Irf8 | 2.49 | 0.013613 | 0.36 | NM_008320.3 |
| Cxcr3 | 2.48 | 0.000831 | 0.1 | NM_009910.2 |
| Il2rg | 2.48 | 0.026262 | 0.5 | NM_013563.3 |
| B2m | 2.46 | 0.024468 | 0.47 | NM_009735.3 |
| H2-K1 | 2.46 | 0.068178 | 0.89 | NM_001001892.2 |
| Il10 | 2.45 | 0.055919 | 0.77 | NM_010548.1 |
| Ccl12 | 2.43 | 0.003486 | 0.18 | NM_011331.2 |
| Ikbke | 2.41 | 0.007361 | 0.27 | NM_019777.3 |
| Ly86 | 2.38 | 0.043064 | 0.65 | NM_010745.2 |
| Ikzf3 | 2.37 | 0.027547 | 0.5 | NM_011771.1 |
| Tnfaip3 | 2.36 | 0.016083 | 0.37 | NM_009397.2 |
| Ptafr | 2.34 | 0.000422 | 0.07 | NM_001081211.1 |
| Psmb10 | 2.33 | 0.011962 | 0.34 | NM_013640.3 |
| Il16 | 2.31 | 0.030691 | 0.54 | NM_010551.3 |
| Il10ra | 2.28 | 0.006071 | 0.24 | NM_008348.2 |
| Il27ra | 2.28 | 0.001762 | 0.14 | NM_016671.3 |
| Bcl3 | 2.21 | 0.00139 | 0.12 | NM_033601.3 |
| Xcl1 | 2.21 | 0.013076 | 0.35 | NM_008510.1 |
| Cd2 | 2.19 | 0.052236 | 0.73 | NM_013486.2 |
| Cd74 | 2.18 | 0.009063 | 0.3 | NM_001042605.1 |
| C2 | 2.17 | 0.003178 | 0.17 | NM_013484.2 |
| Nod2 | 2.17 | 0.024175 | 0.47 | NM_145857.2 |
| Sele | 2.14 | 0.029452 | 0.52 | NM_011345.2 |
| Ifih1 | 2.11 | 0.002862 | 0.16 | NM_027835.2 |
| Irf5 | 2.11 | 0.027472 | 0.5 | NM_012057.3 |
| Cd80 | 2.06 | 0.019858 | 0.43 | NM_009855.2 |
| Sell | 2.06 | 0.012811 | 0.35 | NM_001164059.1 |
| H2-Ab1 | 2.05 | 0.027782 | 0.5 | NM_207105.2 |
| Eomes | 2.04 | 0.001604 | 0.13 | NM_010136.2 |
| Fyn | 2.03 | 0.020363 | 0.43 | NM_008054.2 |
| Cd1d1 | 2.02 | 0.070169 | 0.91 | NM_007639.3 |
| Tgfbi | 2 | 0.016232 | 0.37 | NM_009369.4 |
| Cd27 | 1.99 | 0.006168 | 0.24 | NM_001042564.1 |
| Prdm1 | 1.97 | 0.042587 | 0.65 | NM_007548.3 |
| Gfi1 | 1.93 | 0.014241 | 0.36 | NM_010278.2 |
| Ddx58 | 1.92 | 0.009535 | 0.3 | NM_172689.3 |
| H2-Aa | 1.9 | 0.050264 | 0.72 | NM_010378.2 |
| C1qb | 1.89 | 0.030014 | 0.53 | NM_009777.2 |
| Ebi3 | 1.84 | 0.014616 | 0.36 | NM_015766.2 |
| Abcb1a | 1.78 | 0.006839 | 0.25 | NM_011076.1 |
| Map4k1 | 1.78 | 0.052029 | 0.73 | NM_008279.2 |
| Csf1 | 1.75 | 0.037433 | 0.6 | NM_001113530.1 |
| H2-Q10 | 1.74 | 0.017299 | 0.38 | NM_010391.4 |
| Tnfsf10 | 1.74 | 0.131207 | 1 | NM_009425.2 |
| Casp8 | 1.71 | 0.012762 | 0.35 | NM_009812.2 |
| Nfatc2 | 1.7 | 0.006126 | 0.24 | NM_001037177.1 |
| Cd86 | 1.69 | 0.014756 | 0.36 | NM_019388.3 |
| Zap70 | 1.67 | 0.016421 | 0.37 | NM_009539.2 |
| Bid | 1.64 | 0.008157 | 0.28 | NM_007544.3 |
| C1qa | 1.64 | 0.070709 | 0.91 | NM_007572.2 |
| Lilra6 | 1.64 | 0.009589 | 0.3 | NM_011090.2 |
| Cd48 | 1.6 | 0.002037 | 0.14 | NM_007649.4 |
| Slamf1 | 1.59 | 0.042626 | 0.65 | NM_013730.4 |
| Pml | 1.58 | 0.032156 | 0.55 | NM_008884.2 |
| Vcam1 | 1.58 | 0.02157 | 0.44 | NM_011693.2 |
| Ccr2 | 1.57 | 0.007123 | 0.26 | NM_009915.2 |
| Cd7 | 1.57 | 0.006179 | 0.24 | NM_009854.1 |
| Ccrl2 | 1.56 | 0.079421 | 0.97 | NM_017466.4 |
| Ms4a1 | 1.56 | 0.346634 | 1 | NM_007641.5 |
| Ctsg | 1.55 | 0.140206 | 1 | NM_007800.1 |
| H2-DMa | 1.54 | 0.013098 | 0.35 | NM_010386.3 |
| Il15ra | 1.54 | 0.002536 | 0.16 | NM_008358.2 |
| Tlr2 | 1.54 | 0.042111 | 0.65 | NM_011905.2 |
| Il6 | 1.53 | 0.082164 | 0.99 | NM_031168.1 |
| Irak3 | 1.53 | 0.000544 | 0.08 | NM_028679.3 |
| Irak4 | 1.53 | 0.038175 | 0.61 | NM_029926.5 |
| Crlf2 | 1.51 | 0.085702 | 1 | NM_001164735.1 |
| Card9 | 1.5 | 0.057584 | 0.78 | NM_001037747.1 |
| Klra5 | 1.5 | 0.132015 | 1 | NM_008463.2 |
| C1s | 1.49 | 0.031831 | 0.55 | NM_144938.2 |
| Ly96 | 1.47 | 0.169424 | 1 | NM_016923.1 |
| G6pdx | 1.47 | 0.001313 | 0.12 | NM_008062.2 |
| Nfkbiz | 1.46 | 0.005634 | 0.24 | NM_030612.1 |
| Lef1 | 1.43 | 0.015078 | 0.36 | NM_010703.3 |
| Plau | 1.42 | 0.066317 | 0.87 | NM_008873.2 |
| Tnfrsf8 | 1.39 | 0.077939 | 0.96 | NM_009401.2 |
| Csf2rb | 1.35 | 0.104285 | 1 | NM_007780.4 |
| Icam1 | 1.35 | 0.041652 | 0.64 | NM_010493.2 |
| Tapbp | 1.35 | 0.01088 | 0.32 | NM_009318.2 |
| Folr4 | 1.34 | 0.102456 | 1 | NM_022888.2 |
| Il4ra | 1.33 | 0.136546 | 1 | NM_001008700.3 |
| Prim1 | 1.33 | 0.000782 | 0.09 | NM_008921.2 |
| Ikzf1 | 1.32 | 0.059313 | 0.8 | NM_001025597.1 |
| Tgfb1 | 1.29 | 0.188729 | 1 | NM_011577.1 |
| Ctsc | 1.28 | 0.028287 | 0.51 | NM_009982.2 |
| Cd4 | 1.25 | 0.011513 | 0.34 | NM_013488.2 |
| Hif1a | 1.25 | 0.004077 | 0.2 | NM_010431.1 |
| Cd226 | 1.24 | 0.093338 | 1 | NM_001039149.1 |
| Cd14 | 1.21 | 0.000585 | 0.08 | NM_009841.3 |
| Cx3cr1 | 1.19 | 0.079506 | 0.97 | NM_009987.3 |
| Fkbp5 | 1.18 | 0.069409 | 0.9 | NM_010220.3 |
| Cdkn1a | 1.17 | 0.035306 | 0.58 | NM_007669.4 |
| Relb | 1.17 | 0.019296 | 0.42 | NM_009046.2 |
| Casp3 | 1.15 | 0.007261 | 0.27 | NM_009810.2 |
| Il1a | 1.15 | 0.178463 | 1 | NM_010554.4 |
| Tlr9 | 1.13 | 0.005422 | 0.23 | NM_031178.2 |
| Klrc3 | 1.11 | 0.305125 | 1 | NM_021378.1 |
| Ifitm1 | 1.09 | 0.003032 | 0.17 | NM_001112715.1 |
| Mx1 | 1.07 | 0.018447 | 0.4 | NM_010846.1 |
| Prkcd | 1.07 | 0.021058 | 0.43 | NM_011103.2 |
| Cxcl1 | 1.06 | 0.128599 | 1 | NM_008176.1 |
| Nfkb2 | 1.06 | 0.009884 | 0.31 | NM_019408.2 |
| Ceacam1 | 1.05 | 0.524505 | 1 | NM_001039185.1 |
| Cd40 | 1.04 | 0.151263 | 1 | NM_011611.2 |
| Selplg | 1.02 | 0.22268 | 1 | NM_009151.3 |
| Irf4 | 1.01 | 0.015168 | 0.36 | NM_013674.1 |
| Ptger4 | 0.99 | 0.015221 | 0.36 | NM_008965.1 |
| Ifi35 | 0.96 | 0.010297 | 0.32 | NM_027320.4 |
| Jak3 | 0.96 | 0.122308 | 1 | NM_010589.5 |
| Tnfrsf14 | 0.96 | 0.14266 | 1 | NM_178931.2 |
| Cd160 | 0.95 | 0.071494 | 0.91 | NM_001163496.1 |
| Ccr9 | 0.94 | 0.070743 | 0.91 | NM_009913.6 |
| Tbk1 | 0.94 | 0.016753 | 0.38 | NM_019786.4 |
| Litaf | 0.93 | 0.022932 | 0.45 | NM_019980.1 |
| Myd88 | 0.93 | 0.067744 | 0.89 | NM_010851.2 |
| Batf3 | 0.92 | 0.002655 | 0.16 | NM_030060.2 |
| Il1rl1 | 0.92 | 0.020045 | 0.43 | NM_001025602.2 |
| Tcf7 | 0.92 | 0.260626 | 1 | NM_009331.3 |
| C1ra | 0.9 | 0.068127 | 0.89 | NM_023143.3 |
| Serping1 | 0.88 | 0.073193 | 0.92 | NM_009776.3 |
| C4a | 0.87 | 0.014649 | 0.36 | NM_011413.2 |
| Jak1 | 0.87 | 0.07295 | 0.92 | NM_146145.2 |
| Plaur | 0.86 | 0.151026 | 1 | NM_011113.3 |
| Ptpn2 | 0.86 | 0.015214 | 0.36 | NM_001127177.1 |
| Pecam1 | 0.85 | 0.351892 | 1 | NM_008816.2 |
| Gapdh | 0.85 | 0.008108 | 0.28 | NM_001001303.1 |
| Ccl19 | 0.84 | 0.27449 | 1 | NM_011888.2 |
| Cd34 | 0.82 | 0.294485 | 1 | NM_001111059.1 |
| Csf2 | 0.81 | 0.269758 | 1 | NM_009969.4 |
| Tyk2 | 0.81 | 0.18757 | 1 | NM_018793.2 |
| Ets1 | 0.79 | 0.235785 | 1 | NM_001038642.1 |
| Tlr8 | 0.78 | 0.309675 | 1 | NM_133212.2 |
| Entpd1 | 0.77 | 0.270513 | 1 | NM_009848.3 |
| Mapkapk2 | 0.75 | 0.000192 | 0.05 | NM_008551.1 |
| Il18 | 0.73 | 0.23891 | 1 | NM_008360.1 |
| Nfkb1 | 0.73 | 0.07719 | 0.96 | NM_008689.2 |
| Ifngr2 | 0.72 | 0.196356 | 1 | NM_008338.3 |
| H2-Eb1 | 0.71 | 0.192959 | 1 | NM_010382.2 |
| Socs3 | 0.71 | 0.02463 | 0.47 | NM_007707.2 |
| Tnfrsf1b | 0.71 | 0.23831 | 1 | NM_011610.3 |
| Abcf1 | 0.7 | 0.009082 | 0.3 | NM_013854.1 |
| Cd19 | 0.7 | 0.623964 | 1 | NM_009844.2 |
| Il12b | 0.7 | 0.346095 | 1 | NM_008352.1 |
| Clec4a4 | 0.69 | 0.176404 | 1 | NM_001005860.2 |
| Ifnar2 | 0.69 | 0.020554 | 0.43 | NM_001110498.1 |
| Klrc2 | 0.69 | 0.211391 | 1 | NM_001098669.1 |
| Ilf3 | 0.67 | 0.026869 | 0.5 | NM_010561.2 |
| Gusb | 0.67 | 0.149453 | 1 | NM_010368.1 |
| Gpr183 | 0.66 | 0.052074 | 0.73 | NM_183031.2 |
| Nfatc3 | 0.66 | 0.021179 | 0.43 | NM_010901.2 |
| Nfkbia | 0.66 | 0.077314 | 0.96 | NM_010907.2 |
| Btk | 0.65 | 0.315236 | 1 | NM_013482.2 |
| Cd96 | 0.65 | 0.186828 | 1 | NM_032465.2 |
| Itgax | 0.64 | 0.433129 | 1 | NM_021334.2 |
| Jak2 | 0.64 | 0.067306 | 0.88 | NM_001048177.1 |
| Itga5 | 0.62 | 0.19662 | 1 | NM_010577.3 |
| C3 | 0.59 | 0.140802 | 1 | NM_009778.2 |
| Stat5a | 0.58 | 0.036499 | 0.59 | NM_011488.2 |
| Ccl9 | 0.53 | 0.355877 | 1 | NM_011338.2 |
| Ifnb1 | 0.52 | 0.460185 | 1 | NM_010510.1 |
| Tmem173 | 0.52 | 0.059301 | 0.8 | NM_028261.1 |
| Cr2 | 0.51 | 0.685482 | 1 | NM_007758.2 |
| Ifngr1 | 0.51 | 0.160039 | 1 | NM_010511.2 |
| Polr2a | 0.51 | 0.058266 | 0.79 | NM_009089.2 |
| Cebpb | 0.5 | 0.047235 | 0.7 | NM_009883.3 |
| Psmb7 | 0.5 | 0.010355 | 0.32 | NM_011187.1 |
| Stat3 | 0.5 | 0.05756 | 0.78 | NM_213659.2 |
| Syk | 0.49 | 0.510664 | 1 | NM_011518.2 |
| Aicda | 0.48 | 0.538637 | 1 | NM_009645.2 |
| C1qbp | 0.48 | 0.033137 | 0.55 | NM_007573.2 |
| Cd40lg | 0.48 | 0.246282 | 1 | NM_011616.2 |
| Mif | 0.48 | 0.051095 | 0.73 | NM_010798.2 |
| Hcst | 0.47 | 0.263159 | 1 | NM_011827.3 |
| Tnfsf14 | 0.46 | 0.026898 | 0.5 | NM_019418.2 |
| Kir3dl1 | 0.45 | 0.399472 | 1 | NM_177749.3 |
| Mapk11 | 0.45 | 0.539083 | 1 | NM_011161.5 |
| Nfatc1 | 0.45 | 0.439238 | 1 | NM_016791.4 |
| Csf1r | 0.43 | 0.215078 | 1 | NM_001037859.1 |
| Ltb4r1 | 0.42 | 0.308986 | 1 | NM_008519.2 |
| Fas | 0.41 | 0.320072 | 1 | NM_007987.2 |
| Icam2 | 0.4 | 0.607705 | 1 | NM_010494.1 |
| Psmc2 | 0.39 | 0.080089 | 0.97 | NM_011188.3 |
| Traf1 | 0.38 | 0.170878 | 1 | NM_009421.3 |
| Il22 | 0.34 | 0.712411 | 1 | NM_016971.1 |
| Atm | 0.33 | 0.354044 | 1 | NM_007499.1 |
| Bax | 0.33 | 0.180717 | 1 | NM_007527.3 |
| Nox4 | 0.33 | 0.464895 | 1 | NM_015760.4 |
| Ccl6 | 0.31 | 0.691329 | 1 | NM_009139.2 |
| Nfil3 | 0.3 | 0.290088 | 1 | NM_017373.3 |
| Nox1 | 0.3 | 0.730345 | 1 | NM_172203.1 |
| Tlr4 | 0.3 | 0.321192 | 1 | NM_021297.2 |
| Cx3cl1 | 0.27 | 0.474307 | 1 | NM_009142.3 |
| Fcgr2b | 0.27 | 0.395729 | 1 | NM_001077189.1 |
| Il3 | 0.26 | 0.652264 | 1 | NM_010556.4 |
| Tlr3 | 0.26 | 0.320723 | 1 | NM_126166.2 |
| Cxcr5 | 0.25 | 0.720601 | 1 | NM_007551.2 |
| Foxp3 | 0.25 | 0.576396 | 1 | NM_054039.1 |
| Cd79b | 0.23 | 0.715769 | 1 | NM_008339.2 |
| Pdcd1lg2 | 0.22 | 0.768478 | 1 | NM_021396.2 |
| Gm10499 | 0.21 | 0.522846 | 1 | XM_003086920.1 |
| Tyrobp | 0.21 | 0.475211 | 1 | NM_011662.2 |
| Cdh5 | 0.16 | 0.879201 | 1 | NM_009868.3 |
| Gata3 | 0.14 | 0.771315 | 1 | NM_008091.3 |
| Rpl19 | 0.14 | 0.063824 | 0.85 | NM_009078.2 |
| Traf6 | 0.12 | 0.498314 | 1 | NM_009424.2 |
| Il17b | 0.11 | 0.852165 | 1 | NM_019508.1 |
| Irak2 | 0.1 | 0.565733 | 1 | NM_001113553.1 |
| Klrb1 | 0.1 | 0.876998 | 1 | NM_001099918.1 |
| Xcr1 | 0.1 | 0.850775 | 1 | NM_011798.4 |
| Ppia | 0.09 | 0.366961 | 1 | NM_008907.1 |
| Ifnar1 | 0.08 | 0.76607 | 1 | NM_010508.1 |
| Psmb5 | 0.08 | 0.286258 | 1 | NM_011186.1 |
| Tnfsf8 | 0.07 | 0.767314 | 1 | NM_009403.2 |
| Il10rb | 0.06 | 0.800088 | 1 | NM_008349.5 |
| Il2 | 0.06 | 0.963575 | 1 | NM_008366.2 |
| Nt5e | 0.06 | 0.91975 | 1 | NM_011851.3 |
| Cd44 | 0.05 | 0.786386 | 1 | NM_009851.2 |
| Mapk14 | 0.05 | 0.801687 | 1 | NM_011951.2 |
| Traf2 | 0.04 | 0.828909 | 1 | NM_009422.2 |
| Ccr7 | 0.03 | 0.961921 | 1 | NM_007719.2 |
| Il17f | 0.03 | 0.909934 | 1 | NM_145856.2 |
| Maf | 0.02 | 0.946031 | 1 | NM_001025577.2 |
| Ccr10 | 0.01 | 0.991545 | 1 | NM_007721.4 |
| Mapk1 | 0.01 | 0.946126 | 1 | NM_011949.3 |
| Notch1 | 0.01 | 0.970174 | 1 | NM_008714.2 |
| Cd82 | 0 | 0.989821 | 1 | NM_001271430.1 |
| Klra6 | -0.02 | 0.92492 | 1 | NM_008464.2 |
| Eef1g | -0.04 | 0.727463 | 1 | NM_026007.4 |
| Il15 | -0.06 | 0.931148 | 1 | NM_008357.2 |
| Itga6 | -0.06 | 0.909673 | 1 | NM_008397.3 |
| Kir3dl2 | -0.06 | 0.936268 | 1 | NM_177748.2 |
| Tgfbr2 | -0.07 | 0.766708 | 1 | NM_009371.2 |
| Sdha | -0.08 | 0.60929 | 1 | NM_023281.1 |
| Irak1 | -0.1 | 0.640835 | 1 | NM_008363.2 |
| Lta | -0.11 | 0.871919 | 1 | NM_010735.1 |
| Oaz1 | -0.12 | 0.126462 | 1 | NM_008753.4 |
| Cd164 | -0.14 | 0.517955 | 1 | NM_016898.2 |
| Tbp | -0.14 | 0.225269 | 1 | NM_013684.3 |
| Atg16l1 | -0.15 | 0.366726 | 1 | NM_029846.3 |
| Pdgfb | -0.15 | 0.836746 | 1 | NM_011057.3 |
| Tnfrsf11a | -0.15 | 0.578059 | 1 | NM_009399.3 |
| Tnfsf11 | -0.15 | 0.460449 | 1 | NM_011613.3 |
| Klra21 | -0.16 | 0.789246 | 1 | NM_053151.1 |
| Cmklr1 | -0.18 | 0.407276 | 1 | NM_008153.3 |
| Tnfsf13b | -0.18 | 0.629412 | 1 | NM_033622.1 |
| Cxcl3 | -0.19 | 0.760819 | 1 | NM_203320.2 |
| Fcamr | -0.2 | 0.729963 | 1 | NM_001170632.1 |
| Il18r1 | -0.2 | 0.124912 | 1 | NM_001161842.1 |
| Il23r | -0.2 | 0.530829 | 1 | NM_144548.1 |
| Rela | -0.2 | 0.529787 | 1 | NM_009045.4 |
| Cxcr4 | -0.22 | 0.330408 | 1 | NM_009911.3 |
| Sigirr | -0.23 | 0.243022 | 1 | NM_023059.3 |
| Ube2l3 | -0.23 | 0.224461 | 1 | NM_009456.2 |
| App | -0.24 | 0.588084 | 1 | NM_007471.2 |
| Psmd7 | -0.24 | 0.226046 | 1 | NM_010817.2 |
| Tnfaip6 | -0.24 | 0.509932 | 1 | NM_009398.2 |
| Il4 | -0.25 | 0.751669 | 1 | NM_021283.1 |
| Masp2 | -0.25 | 0.760691 | 1 | NM_010767.3 |
| Pdcd2 | -0.27 | 0.28026 | 1 | NM_008799.2 |
| Phlpp2 | -0.27 | 0.576894 | 1 | NM_001122594.2 |
| Ikzf2 | -0.28 | 0.366817 | 1 | NM_011770.4 |
| Xbp1 | -0.28 | 0.289686 | 1 | NM_013842.2 |
| Map4k4 | -0.3 | 0.591843 | 1 | NM_008696.2 |
| Stat6 | -0.3 | 0.180905 | 1 | NM_009284.2 |
| Cish | -0.31 | 0.289785 | 1 | NM_009895.3 |
| Pla2g2a | -0.31 | 0.473579 | 1 | NM_001082531.1 |
| Tubb5 | -0.33 | 0.137938 | 1 | NM_011655.4 |
| Mbl2 | -0.34 | 0.621635 | 1 | NM_010776.1 |
| Itgb1 | -0.35 | 0.36179 | 1 | NM_010578.1 |
| Tgfbr1 | -0.35 | 0.397952 | 1 | NM_009370.2 |
| Fn1 | -0.36 | 0.615966 | 1 | NM_010233.1 |
| Irf3 | -0.36 | 0.235681 | 1 | NM_016849.3 |
| Ptgs2 | -0.37 | 0.568515 | 1 | NM_011198.3 |
| Tnfrsf13c | -0.37 | 0.610979 | 1 | NM_028075.2 |
| Ltbr | -0.38 | 0.047163 | 0.7 | NM_010736.3 |
| Tnfsf18 | -0.38 | 0.523481 | 1 | NM_183391.3 |
| Notch2 | -0.39 | 0.039018 | 0.62 | NM_010928.1 |
| Cxcl15 | -0.4 | 0.218952 | 1 | NM_011339.2 |
| Tnfsf12 | -0.4 | 0.337952 | 1 | NM_011614.3 |
| Traf3 | -0.41 | 0.13856 | 1 | NM_001048206.1 |
| Il12a | -0.43 | 0.151582 | 1 | NM_008351.1 |
| Fcer1a | -0.44 | 0.323173 | 1 | NM_010184.1 |
| Traf5 | -0.44 | 0.483494 | 1 | NM_011633.1 |
| Gp1bb | -0.45 | 0.474227 | 1 | NM_010327.2 |
| Nos2 | -0.45 | 0.405965 | 1 | NM_010927.3 |
| Chuk | -0.46 | 0.014035 | 0.36 | NM_001162410.1 |
| Gpi1 | -0.47 | 0.047819 | 0.7 | NM_008155.3 |
| Pdgfrb | -0.47 | 0.494123 | 1 | NM_008809.1 |
| Rag2 | -0.47 | 0.271011 | 1 | NM_009020.3 |
| Igf2r | -0.48 | 0.049439 | 0.72 | NM_010515.1 |
| Lif | -0.48 | 0.207357 | 1 | NM_008501.2 |
| Tal1 | -0.49 | 0.586007 | 1 | NM_011527.2 |
| Bcl6 | -0.5 | 0.091682 | 1 | NM_009744.3 |
| Il17a | -0.51 | 0.39014 | 1 | NM_010552.3 |
| Il19 | -0.51 | 0.414393 | 1 | NM_001009940.1 |
| Il25 | -0.51 | 0.603061 | 1 | NM_080729.2 |
| Map4k2 | -0.51 | 0.206988 | 1 | NM_009006.2 |
| Polr1b | -0.51 | 0.206382 | 1 | NM_009086.2 |
| Il17rb | -0.52 | 0.24305 | 1 | NM_019583.3 |
| C7 | -0.53 | 0.325502 | 1 | XM_356827.6 |
| Ikbkb | -0.54 | 0.305317 | 1 | NM_010546.2 |
| Icam4 | -0.55 | 0.40783 | 1 | NM_023892.2 |
| Zeb1 | -0.55 | 0.334518 | 1 | NM_011546.2 |
| Lilra5 | -0.56 | 0.365159 | 1 | NM_001081239.2 |
| Rae1 | -0.56 | 0.080897 | 0.97 | NM_175112.5 |
| Smad3 | -0.56 | 0.085099 | 1 | NM_016769.3 |
| Tcf4 | -0.56 | 0.119266 | 1 | NM_013685.1 |
| Tnfsf15 | -0.57 | 0.202326 | 1 | NM_177371.3 |
| Abl1 | -0.58 | 0.146305 | 1 | NM_009594.3 |
| Bcl2 | -0.58 | 0.095712 | 1 | NM_009741.3 |
| Cxcl12 | -0.58 | 0.493942 | 1 | NM_021704.3 |
| Nox3 | -0.58 | 0.194952 | 1 | NM_198958.2 |
| Tollip | -0.59 | 0.093115 | 1 | NM_023764.3 |
| C8g | -0.6 | 0.216014 | 1 | NM_027062.1 |
| Pla2g2e | -0.6 | 0.312176 | 1 | NM_012044.2 |
| Stat5b | -0.6 | 0.169671 | 1 | NM_011489.3 |
| Ccbp2 | -0.61 | 0.125225 | 1 | NM_021609.3 |
| C4bp | -0.62 | 0.45803 | 1 | NM_007576.3 |
| Cd28 | -0.62 | 0.344822 | 1 | NM_007642.4 |
| Ccl25 | -0.63 | 0.052073 | 0.73 | NM_009138.3 |
| Defb14 | -0.65 | 0.395199 | 1 | NM_183026.2 |
| Il1rap | -0.66 | 0.00095 | 0.1 | NM_134103.2 |
| Icosl | -0.67 | 0.080469 | 0.97 | NM_015790.3 |
| Trp53 | -0.67 | 0.074183 | 0.93 | NM_011640.1 |
| Bcap31 | -0.68 | 0.000319 | 0.06 | NM_012060.4 |
| Cradd | -0.7 | 0.002519 | 0.16 | NM_009950.2 |
| Tirap | -0.7 | 0.001382 | 0.12 | NM_001177847.1 |
| Ikbkg | -0.72 | 0.011487 | 0.34 | NM_178590.2 |
| Il23a | -0.72 | 0.176042 | 1 | NM_031252.1 |
| Phlpp1 | -0.72 | 0.124797 | 1 | NM_133821.3 |
| Ikbkap | -0.73 | 0.078953 | 0.97 | NM_026079.3 |
| Aire | -0.74 | 0.435047 | 1 | NM_009646.1 |
| Rag1 | -0.74 | 0.179885 | 1 | NM_009019.2 |
| Ccr6 | -0.76 | 0.289436 | 1 | NM_001190333.1 |
| Hc | -0.76 | 0.29447 | 1 | NM_010406.1 |
| Klra1 | -0.76 | 0.195714 | 1 | NM_016659.3 |
| Btla | -0.78 | 0.2267 | 1 | NM_177584.3 |
| Ccl22 | -0.78 | 0.229587 | 1 | NM_009137.2 |
| Il13ra1 | -0.78 | 0.029014 | 0.52 | NM_133990.4 |
| Itga2b | -0.78 | 0.313996 | 1 | NM_010575.2 |
| Src | -0.78 | 0.024429 | 0.47 | NM_001025395.2 |
| Hprt | -0.8 | 0.025424 | 0.48 | NM_013556.2 |
| Fcgrt | -0.81 | 0.008755 | 0.29 | NM_010189.3 |
| Tslp | -0.81 | 0.039137 | 0.62 | NM_021367.1 |
| Fadd | -0.82 | 0.038956 | 0.62 | NM_010175.5 |
| Kit | -0.84 | 0.32933 | 1 | NM_001122733.1 |
| Cd59b | -0.85 | 0.197465 | 1 | NM_181858.1 |
| Tnfrsf13b | -0.86 | 0.01259 | 0.35 | NM_021349.1 |
| Ptk2 | -0.87 | 0.145046 | 1 | NM_007982.2 |
| Dpp4 | -0.88 | 0.159512 | 1 | NM_001159543.1 |
| Blnk | -0.89 | 0.003127 | 0.17 | NM_008528.4 |
| Mbp | -0.9 | 0.005931 | 0.24 | NM_010777.3 |
| Ccl11 | -0.94 | 0.384573 | 1 | NM_011330.3 |
| Cd81 | -0.94 | 0.003845 | 0.19 | NM_133655.2 |
| Itln1 | -0.94 | 0.431769 | 1 | NM_010584.3 |
| Runx1 | -0.95 | 0.017409 | 0.38 | NM_001111021.1 |
| Ski | -0.97 | 0.021823 | 0.44 | NM_011385.2 |
| Adal | -0.98 | 0.052614 | 0.74 | NM_029475.1 |
| Gpr44 | -0.99 | 0.094003 | 1 | NM_009962.2 |
| Il6st | -1.01 | 0.04521 | 0.68 | NM_010560.2 |
| Npc1 | -1.01 | 0.016629 | 0.38 | NM_008720.2 |
| Il5 | -1.02 | 0.08088 | 0.97 | NM_010558.1 |
| Ifna1 | -1.03 | 0.26148 | 1 | NM_010502.2 |
| Il22ra2 | -1.03 | 0.204805 | 1 | NM_178258.5 |
| Hfe | -1.04 | 0.07321 | 0.92 | NM_010424.4 |
| Ppbp | -1.04 | 0.283331 | 1 | NM_023785.2 |
| Ltf | -1.05 | 0.177096 | 1 | NM_008522.3 |
| Tnfrsf17 | -1.05 | 0.085899 | 1 | NM_011608.1 |
| Cd36 | -1.06 | 0.042539 | 0.65 | NM_007643.3 |
| Vtn | -1.06 | 0.15246 | 1 | NM_011707.2 |
| Il28a | -1.07 | 0.249854 | 1 | NM_001024673.2 |
| Cd97 | -1.09 | 0.147559 | 1 | NM_011925.1 |
| Ikzf4 | -1.09 | 0.048046 | 0.7 | NM_011772.2 |
| Ccl26 | -1.11 | 0.13082 | 1 | NM_001013412.2 |
| Ccr3 | -1.11 | 0.204347 | 1 | NM_009914.4 |
| Defb1 | -1.11 | 0.23368 | 1 | NM_007843.3 |
| Il7r | -1.11 | 0.006278 | 0.24 | NM_008372.3 |
| Cxcr1 | -1.14 | 0.141003 | 1 | NM_178241.4 |
| Cfi | -1.18 | 0.192861 | 1 | NM_007686.2 |
| Tfrc | -1.2 | 0.005236 | 0.23 | NM_011638.3 |
| C8a | -1.21 | 0.145266 | 1 | NM_146148.1 |
| Cul9 | -1.21 | 0.005904 | 0.24 | NM_001081335.2 |
| Frmpd4 | -1.21 | 0.13625 | 1 | NM_001033330.2 |
| Cd9 | -1.22 | 0.00431 | 0.2 | NM_007657.3 |
| Ifna2 | -1.23 | 0.056671 | 0.78 | NM_010503.2 |
| Il1rl2 | -1.25 | 0.000184 | 0.05 | NM_133193.3 |
| Pigr | -1.25 | 0.043259 | 0.65 | NM_011082.3 |
| Traf4 | -1.26 | 6.35E-06 | 0.01 | NM_009423.4 |
| Clu | -1.27 | 0.037262 | 0.6 | NM_013492.2 |
| Cd163 | -1.28 | 0.015613 | 0.37 | NM_053094.2 |
| Cd22 | -1.31 | 0.171021 | 1 | NM_001043317.2 |
| Casp2 | -1.32 | 0.045479 | 0.68 | NM_007610.1 |
| Cfh | -1.32 | 0.020128 | 0.43 | NM_009888.3 |
| Mme | -1.32 | 0.033775 | 0.56 | NM_008604.3 |
| Trem2 | -1.32 | 0.222592 | 1 | NM_031254.2 |
| Zbtb7b | -1.35 | 0.014726 | 0.36 | NM_009565.4 |
| Cd83 | -1.39 | 0.060383 | 0.81 | NM_009856.2 |
| H2-Ea-ps | -1.41 | 0.764081 | 1 | NM_010381.2 |
| Pparg | -1.42 | 0.028862 | 0.52 | NM_011146.1 |
| Smad5 | -1.45 | 0.022002 | 0.44 | NM_008541.2 |
| Cd46 | -1.47 | 0.118224 | 1 | NM_010778.3 |
| Ctnnb1 | -1.47 | 0.004739 | 0.22 | NM_007614.2 |
| Icam5 | -1.47 | 0.037189 | 0.6 | NM_008319.2 |
| Psmb11 | -1.47 | 0.085657 | 1 | NM_175204.4 |
| Rorc | -1.47 | 0.005251 | 0.23 | NM_011281.2 |
| Il33 | -1.48 | 0.073506 | 0.92 | NM_133775.1 |
| Il17re | -1.49 | 0.006552 | 0.25 | NM_001034029.1 |
| Btnl2 | -1.5 | 0.04774 | 0.7 | NM_079835.2 |
| Hlx | -1.52 | 0.030888 | 0.54 | NM_008250.2 |
| Cd99 | -1.53 | 0.002718 | 0.16 | NM_025584.2 |
| Abcb10 | -1.54 | 0.032988 | 0.55 | NM_019552.2 |
| Cd79a | -1.56 | 0.290795 | 1 | NM_007655.3 |
| Masp1 | -1.57 | 0.01504 | 0.36 | NM_008555.2 |
| Il6ra | -1.62 | 0.043499 | 0.65 | NM_010559.2 |
| Pax5 | -1.62 | 0.177064 | 1 | NM_008782.2 |
| Btnl1 | -1.64 | 0.03547 | 0.58 | NM_001111094.1 |
| Ahr | -1.65 | 0.003438 | 0.18 | NM_013464.4 |
| C8b | -1.66 | 0.097565 | 1 | NM_133882.2 |
| Il20 | -1.66 | 0.134182 | 1 | NM_021380.1 |
| Cd55 | -1.69 | 0.002445 | 0.16 | NM_010016.2 |
| Cd244 | -1.7 | 0.062784 | 0.84 | NM_018729.2 |
| Hamp | -1.7 | 0.255789 | 1 | NM_032541.1 |
| Mr1 | -1.71 | 0.000132 | 0.04 | NM_008209.4 |
| Alas1 | -1.72 | 0.039416 | 0.62 | NM_020559.2 |
| Ccl24 | -1.76 | 0.1117 | 1 | NM_019577.4 |
| Cd109 | -1.76 | 0.093694 | 1 | NM_153098.3 |
| Ccr4 | -1.78 | 0.070807 | 0.91 | NM_009916.2 |
| Il9 | -1.81 | 0.188203 | 1 | NM_008373.1 |
| Tgfb2 | -1.81 | 0.033149 | 0.55 | NM_009367.1 |
| Ltb4r2 | -1.82 | 0.009825 | 0.31 | NM_020490.2 |
| C6 | -1.84 | 0.010696 | 0.32 | NM_016704.2 |
| C9 | -1.86 | 0.075382 | 0.94 | NM_013485.1 |
| Cd3eap | -1.87 | 0.013821 | 0.36 | NM_145822.2 |
| H2-Ob | -1.88 | 0.054008 | 0.74 | NM_010389.3 |
| Il13 | -1.89 | 0.083794 | 1 | NM_008355.2 |
| Ccl20 | -1.9 | 0.022256 | 0.44 | NM_016960.1 |
| Tlr5 | -1.95 | 0.000701 | 0.09 | NM_016928.2 |
| Muc1 | -2.02 | 0.008963 | 0.3 | NM_013605.1 |
| Tgfb3 | -2.05 | 0.004403 | 0.21 | NM_009368.2 |
| Cd24a | -2.09 | 0.014736 | 0.36 | NM_009846.2 |
| Lcp2 | -2.19 | 0.016369 | 0.37 | NM_010696.3 |
| Il1r1 | -2.4 | 0.001901 | 0.14 | NM_001123382.1 |
| Ncam1 | -2.66 | 0.008238 | 0.28 | NM_001113204.1 |
| Cfd | -2.76 | 0.052171 | 0.73 | NM_013459.1 |
| Il7 | -2.78 | 0.002578 | 0.16 | NM_008371.2 |
| Il11ra1 | -2.79 | 5.95E-05 | 0.03 | NM_010549.3 |
| Ccrl1 | -2.89 | 0.021973 | 0.44 | NM_145700.2 |
| Cd209g | -3.51 | 0.001903 | 0.14 | NM_027343.3 |
| Ccr8 | -4.69 | 0.004786 | 0.22 | NM_007720.2 |
