# Supplementary material for: Host transcriptomic profiling of CD-1 outbred mice with severe clinical outcomes following infection with Orientia tsutsugamushi
Source: PLoS Negl Trop Dis. 2022 Nov 23;16(11):e0010459. doi: 10.1371/journal.pntd.0010459 (PMC9683618; doi:10.1371/journal.pntd.0010459)
Supplement: S4 Table — (DOCX) [file pntd.0010459.s008.docx]

| **Supplemental Table 4. Complete list of differentially expressed genes in lung tissue (D12 vs. Mock)**  *, unadjusted test statistic; **, adjusted test statistic via the Benjamini-Yekutieli method | | | | |
| --- | --- | --- | --- | --- |
| **Gene** | **Log2 fold change** | **P-value*** | **BY P-value**** | **Probe ID** |
| Cxcl9 | 8.23 | 4.141E-05 | 0.04 | NM_008599.2 |
| Cxcl10 | 7.74 | 3.86E-06 | 0.01 | NM_021274.1 |
| Ccl2 | 6.95 | 0.0001511 | 0.05 | NM_011333.3 |
| Gzmb | 6.87 | 6.49E-06 | 0.01 | NM_013542.2 |
| Ifi204 | 6.4 | 0.0093778 | 0.26 | NM_008329.2 |
| Clec4e | 6.28 | 0.004834 | 0.2 | NM_019948.2 |
| Cxcl11 | 6.26 | 0.0000101 | 0.01 | NM_019494.1 |
| Marco | 5.97 | 0.0007084 | 0.08 | NM_010766.2 |
| Klrc1 | 5.8 | 0.0005576 | 0.07 | NM_001136068.1 |
| Ccl4 | 5.75 | 0.0006918 | 0.08 | NM_013652.1 |
| Msr1 | 5.69 | 0.0004962 | 0.07 | NM_001113326.1 |
| Fcgr4 | 5.61 | 0.0085475 | 0.25 | NM_144559.1 |
| Il1r2 | 5.52 | 0.0151331 | 0.34 | NM_010555.4 |
| Ccl8 | 5.49 | 0.0200373 | 0.38 | NM_021443.2 |
| Ccl7 | 5.39 | 0.0004774 | 0.07 | NM_013654.2 |
| Il12rb1 | 5.39 | 0.0533351 | 0.69 | NM_008353.2 |
| Irf7 | 5.38 | 0.0039609 | 0.19 | NM_016850.2 |
| Ccl5 | 5.3 | 0.0160336 | 0.35 | NM_013653.1 |
| Cd6 | 5.15 | 0.0181856 | 0.36 | NM_001037801.2 |
| Clec5a | 5.15 | 0.0033112 | 0.18 | NM_001038604.1 |
| Lilrb4 | 5.15 | 0.0328037 | 0.5 | NM_013532.2 |
| Cd274 | 5.1 | 0.0131725 | 0.32 | NM_021893.2 |
| Lck | 4.86 | 0.0085319 | 0.25 | NM_010693.2 |
| Ctss | 4.83 | 0.0286837 | 0.47 | NM_021281.2 |
| Tigit | 4.83 | 0.0042319 | 0.19 | NM_001146325.1 |
| Cfb | 4.8 | 0.0004915 | 0.07 | NM_008198.2 |
| Cd3e | 4.76 | 0.0017661 | 0.12 | NM_007648.4 |
| S100a9 | 4.69 | 0.0439184 | 0.6 | NM_009114.2 |
| Ccr5 | 4.65 | 0.0004246 | 0.07 | NM_009917.5 |
| Il18rap | 4.65 | 0.0053616 | 0.2 | NM_010553.2 |
| Itgam | 4.6 | 0.0049731 | 0.2 | NM_001082960.1 |
| Ptpn22 | 4.59 | 0.000883 | 0.09 | NM_008979.1 |
| Cxcl13 | 4.58 | 0.029101 | 0.47 | NM_018866.2 |
| Icos | 4.57 | 0.0075393 | 0.24 | NM_017480.1 |
| Ifng | 4.55 | 0.0010622 | 0.09 | NM_008337.1 |
| Klrk1 | 4.55 | 0.0001156 | 0.05 | NM_001083322.1 |
| S100a8 | 4.49 | 0.0411692 | 0.58 | NM_013650.2 |
| Fcgr1 | 4.48 | 0.0001675 | 0.05 | NM_010186.5 |
| Il1rn | 4.43 | 0.0177669 | 0.36 | NM_031167.5 |
| Lair1 | 4.42 | 0.0085164 | 0.25 | NM_001113474.1 |
| H60a | 4.4 | 0.0369929 | 0.54 | NM_010400.2 |
| Cd3d | 4.39 | 0.0068007 | 0.24 | NM_013487.2 |
| Cd8b1 | 4.36 | 0.0004293 | 0.07 | NM_009858.2 |
| Il27 | 4.35 | 0.0268649 | 0.47 | NM_145636.1 |
| Cybb | 4.34 | 0.0413718 | 0.58 | NM_007807.2 |
| Sh2d1a | 4.29 | 0.0033598 | 0.18 | NM_011364.3 |
| Ctla4 | 4.26 | 0.0004572 | 0.07 | NM_009843.3 |
| Il12rb2 | 4.25 | 0.0299356 | 0.47 | NM_008354.3 |
| Fcgr3 | 4.22 | 0.0105519 | 0.28 | NM_010188.5 |
| Socs1 | 4.19 | 0.0003674 | 0.07 | NM_009896.2 |
| Tap1 | 4.19 | 0.0065619 | 0.23 | NM_001161730.1 |
| Tnf | 4.19 | 0.0024667 | 0.15 | NM_013693.1 |
| Itgal | 4.07 | 0.0231744 | 0.42 | NM_008400.2 |
| Lilrb3 | 4.07 | 0.0031731 | 0.17 | NM_011095.2 |
| Thy1 | 4.07 | 0.004251 | 0.19 | NM_009382.3 |
| Ifit2 | 4.06 | 0.0133145 | 0.32 | NM_008332.2 |
| Il21r | 4.04 | 0.0021809 | 0.14 | NM_021887.1 |
| Fcer1g | 4.01 | 0.0131059 | 0.32 | NM_010185.4 |
| Ccl3 | 3.99 | 0.00042 | 0.07 | NM_011337.1 |
| Irgm1 | 3.99 | 0.0003734 | 0.07 | NM_008326.1 |
| Pdcd1 | 3.94 | 0.0001192 | 0.05 | NM_008798.1 |
| Cxcr6 | 3.89 | 0.0002514 | 0.06 | NM_030712.4 |
| Trem1 | 3.86 | 0.028661 | 0.47 | NM_021406.3 |
| Bst1 | 3.75 | 0.0031521 | 0.17 | NM_009763.3 |
| Slamf7 | 3.71 | 5.722E-05 | 0.04 | NM_144539.5 |
| C1qb | 3.7 | 0.0076674 | 0.24 | NM_009777.2 |
| Tbx21 | 3.64 | 0.0024326 | 0.15 | NM_019507.1 |
| Cd8a | 3.63 | 0.0081134 | 0.25 | NM_001081110.2 |
| Fasl | 3.61 | 0.0001202 | 0.05 | NM_010177.3 |
| Cfp | 3.55 | 0.0122864 | 0.31 | NM_008823.3 |
| Emr1 | 3.52 | 0.0204595 | 0.39 | NM_010130.1 |
| C1qa | 3.5 | 0.0141578 | 0.33 | NM_007572.2 |
| Tgfbi | 3.5 | 0.0018898 | 0.13 | NM_009369.4 |
| Ptafr | 3.47 | 0.0005434 | 0.07 | NM_001081211.1 |
| Tagap | 3.44 | 0.0119753 | 0.3 | NM_145968.2 |
| Cxcr3 | 3.41 | 0.0010757 | 0.09 | NM_009910.2 |
| Itga4 | 3.41 | 0.0311759 | 0.49 | NM_010576.3 |
| Spn | 3.4 | 0.0168415 | 0.36 | NM_001037810.1 |
| Ncf4 | 3.34 | 0.0252548 | 0.45 | NM_008677.2 |
| Stat1 | 3.33 | 0.0001616 | 0.05 | NM_009283.3 |
| Arhgdib | 3.31 | 0.0165768 | 0.35 | NM_007486.4 |
| Cd247 | 3.29 | 0.0004672 | 0.07 | NM_001113391.2 |
| Klrd1 | 3.28 | 0.0118607 | 0.3 | NM_010654.2 |
| Tlr1 | 3.27 | 0.022492 | 0.41 | NM_030682.1 |
| Tnfrsf9 | 3.26 | 0.0010484 | 0.09 | NM_001077508.1 |
| Stat2 | 3.25 | 0.0240704 | 0.44 | NM_019963.1 |
| Irf5 | 3.21 | 0.0077341 | 0.24 | NM_012057.3 |
| Cxcr2 | 3.17 | 0.0445936 | 0.61 | NM_009909.3 |
| Casp1 | 3.12 | 0.0090353 | 0.25 | NM_009807.2 |
| Il1a | 3.1 | 0.0279257 | 0.47 | NM_010554.4 |
| Tnfaip3 | 3.08 | 0.0079476 | 0.25 | NM_009397.2 |
| Cd80 | 3.04 | 0.008153 | 0.25 | NM_009855.2 |
| Il21 | 3.03 | 0.0195498 | 0.38 | NM_021782.2 |
| Itgb2 | 2.98 | 0.0086046 | 0.25 | NM_008404.4 |
| Runx3 | 2.98 | 0.0007771 | 0.08 | NM_019732.2 |
| Ly86 | 2.95 | 0.0273314 | 0.47 | NM_010745.2 |
| Psmb9 | 2.91 | 0.014157 | 0.33 | NM_013585.2 |
| Ptprc | 2.89 | 0.0418404 | 0.58 | NM_011210.3 |
| Cd53 | 2.87 | 0.0474336 | 0.64 | NM_007651.3 |
| Irf1 | 2.87 | 0.0059699 | 0.22 | NM_008390.1 |
| Il17ra | 2.81 | 0.0274017 | 0.47 | NM_008359.1 |
| Ptpn6 | 2.8 | 0.0009947 | 0.09 | NM_013545.2 |
| Batf | 2.79 | 0.0013435 | 0.1 | NM_016767.2 |
| Prf1 | 2.77 | 0.0027964 | 0.16 | NM_011073.2 |
| Il10ra | 2.71 | 0.0056786 | 0.21 | NM_008348.2 |
| Il15ra | 2.69 | 0.000232 | 0.06 | NM_008358.2 |
| B2m | 2.64 | 0.0268377 | 0.47 | NM_009735.3 |
| C2 | 2.61 | 0.007807 | 0.24 | NM_013484.2 |
| Tnfrsf4 | 2.57 | 0.043982 | 0.6 | NM_011659.2 |
| Cxcl1 | 2.54 | 0.0144363 | 0.33 | NM_008176.1 |
| Il2rg | 2.54 | 0.0195508 | 0.38 | NM_013563.3 |
| Cd27 | 2.52 | 0.0121301 | 0.3 | NM_001042564.1 |
| Fkbp5 | 2.52 | 0.0085682 | 0.25 | NM_010220.3 |
| Ccl9 | 2.51 | 0.0058336 | 0.21 | NM_011338.2 |
| Cd86 | 2.51 | 0.0037737 | 0.19 | NM_019388.3 |
| Cd69 | 2.5 | 0.0843141 | 0.95 | NM_001033122.3 |
| Ikbke | 2.5 | 0.0075467 | 0.24 | NM_019777.3 |
| Cd2 | 2.48 | 0.0459496 | 0.62 | NM_013486.2 |
| Ikzf3 | 2.48 | 0.0414922 | 0.58 | NM_011771.1 |
| Il1b | 2.48 | 0.0357543 | 0.53 | NM_008361.3 |
| Pou2f2 | 2.47 | 0.0795593 | 0.91 | NM_001163554.1 |
| Gzma | 2.46 | 0.0136028 | 0.32 | NM_010370.2 |
| Bst2 | 2.42 | 0.0147676 | 0.33 | NM_198095.2 |
| Vcam1 | 2.42 | 0.0062075 | 0.22 | NM_011693.2 |
| Csf3r | 2.39 | 0.0523108 | 0.69 | NM_001252651.1 |
| Ltb | 2.39 | 0.0775314 | 0.9 | NM_008518.2 |
| Card9 | 2.38 | 0.0210368 | 0.4 | NM_001037747.1 |
| Crlf2 | 2.37 | 0.0343155 | 0.52 | NM_001164735.1 |
| Il2ra | 2.37 | 0.0256645 | 0.45 | NM_008367.2 |
| Psmb10 | 2.34 | 0.0091163 | 0.25 | NM_013640.3 |
| Cd14 | 2.32 | 0.0144942 | 0.33 | NM_009841.3 |
| Irf8 | 2.31 | 0.0174784 | 0.36 | NM_008320.3 |
| Cd1d1 | 2.3 | 0.0457267 | 0.62 | NM_007639.3 |
| Hif1a | 2.29 | 0.0011183 | 0.09 | NM_010431.1 |
| Nod2 | 2.29 | 0.0291945 | 0.47 | NM_145857.2 |
| Cd5 | 2.28 | 0.0170529 | 0.36 | NM_007650.3 |
| Cd74 | 2.28 | 0.0052208 | 0.2 | NM_001042605.1 |
| Csf1 | 2.25 | 0.0225245 | 0.41 | NM_001113530.1 |
| Stat4 | 2.24 | 0.0069645 | 0.24 | NM_011487.4 |
| Tlr8 | 2.23 | 0.0508263 | 0.67 | NM_133212.2 |
| Nfatc2 | 2.22 | 0.0007688 | 0.08 | NM_001037177.1 |
| Tlr2 | 2.2 | 0.0202768 | 0.39 | NM_011905.2 |
| Il10 | 2.18 | 0.0651155 | 0.79 | NM_010548.1 |
| Il2rb | 2.18 | 0.0252927 | 0.45 | NM_008368.3 |
| H2-K1 | 2.16 | 0.081829 | 0.93 | NM_001001892.2 |
| Lef1 | 2.16 | 0.0074408 | 0.24 | NM_010703.3 |
| Csf2rb | 2.15 | 0.0470107 | 0.63 | NM_007780.4 |
| Xcl1 | 2.15 | 0.0106403 | 0.28 | NM_008510.1 |
| Clec4a4 | 2.12 | 0.0046731 | 0.2 | NM_001005860.2 |
| H2-Ab1 | 2.12 | 0.0320991 | 0.5 | NM_207105.2 |
| Ly96 | 2.12 | 0.0896013 | 0.99 | NM_016923.1 |
| Itga5 | 2.11 | 0.0205441 | 0.39 | NM_010577.3 |
| Map4k1 | 2.1 | 0.0376423 | 0.55 | NM_008279.2 |
| Ifih1 | 2.09 | 0.0051877 | 0.2 | NM_027835.2 |
| Sele | 2.08 | 0.0003561 | 0.07 | NM_011345.2 |
| Cd7 | 2.07 | 0.00186 | 0.13 | NM_009854.1 |
| Bid | 2.05 | 0.0025605 | 0.15 | NM_007544.3 |
| Ccr2 | 2.04 | 0.0009589 | 0.09 | NM_009915.2 |
| Fyn | 2.04 | 0.0322712 | 0.5 | NM_008054.2 |
| Il27ra | 2.04 | 0.0054343 | 0.2 | NM_016671.3 |
| Prdm1 | 2.03 | 0.0335366 | 0.51 | NM_007548.3 |
| Il16 | 2.02 | 0.0294012 | 0.47 | NM_010551.3 |
| Plau | 2 | 0.0347664 | 0.52 | NM_008873.2 |
| Tgfb1 | 1.97 | 0.0999047 | 1 | NM_011577.1 |
| H2-DMa | 1.96 | 0.0039534 | 0.19 | NM_010386.3 |
| Cd160 | 1.94 | 0.0075088 | 0.24 | NM_001163496.1 |
| Tnfrsf8 | 1.92 | 0.0292491 | 0.47 | NM_009401.2 |
| Ciita | 1.91 | 0.038997 | 0.56 | NM_007575.2 |
| Il6 | 1.9 | 0.0701734 | 0.83 | NM_031168.1 |
| H2-DMb2 | 1.89 | 0.0617671 | 0.76 | NM_010388.4 |
| Ccl12 | 1.88 | 0.0052739 | 0.2 | NM_011331.2 |
| Ccrl2 | 1.86 | 0.0530213 | 0.69 | NM_017466.4 |
| H2-Aa | 1.85 | 0.0527402 | 0.69 | NM_010378.2 |
| Ctsc | 1.84 | 0.0181627 | 0.36 | NM_009982.2 |
| Gfi1 | 1.84 | 0.0058481 | 0.21 | NM_010278.2 |
| Casp3 | 1.82 | 0.0052277 | 0.2 | NM_009810.2 |
| Cxcl3 | 1.79 | 0.1589974 | 1 | NM_203320.2 |
| Hcst | 1.79 | 0.0060347 | 0.22 | NM_011827.3 |
| Casp8 | 1.77 | 0.017741 | 0.36 | NM_009812.2 |
| Irak4 | 1.76 | 0.0276192 | 0.47 | NM_029926.5 |
| Tapbp | 1.75 | 0.0042471 | 0.19 | NM_009318.2 |
| Bcl3 | 1.73 | 0.0014115 | 0.1 | NM_033601.3 |
| Fcgr2b | 1.73 | 0.0050661 | 0.2 | NM_001077189.1 |
| Plaur | 1.73 | 0.0281336 | 0.47 | NM_011113.3 |
| Zap70 | 1.72 | 0.0258008 | 0.45 | NM_009539.2 |
| Btk | 1.67 | 0.0727558 | 0.86 | NM_013482.2 |
| Batf3 | 1.66 | 0.001266 | 0.1 | NM_030060.2 |
| Trem2 | 1.63 | 0.055157 | 0.7 | NM_031254.2 |
| Ccl6 | 1.62 | 0.1218392 | 1 | NM_009139.2 |
| Sell | 1.61 | 0.0095782 | 0.26 | NM_001164059.1 |
| Il18 | 1.59 | 0.0693955 | 0.83 | NM_008360.1 |
| Nfkbiz | 1.59 | 0.0052353 | 0.2 | NM_030612.1 |
| Eomes | 1.58 | 0.0038811 | 0.19 | NM_010136.2 |
| Socs3 | 1.57 | 0.0410448 | 0.58 | NM_007707.2 |
| Ctsg | 1.54 | 0.1514919 | 1 | NM_007800.1 |
| Irak3 | 1.54 | 0.0088526 | 0.25 | NM_028679.3 |
| Maf | 1.53 | 0.0123968 | 0.31 | NM_001025577.2 |
| Csf1r | 1.52 | 0.0164107 | 0.35 | NM_001037859.1 |
| Cd48 | 1.5 | 0.0026111 | 0.15 | NM_007649.4 |
| Pml | 1.49 | 0.0259436 | 0.45 | NM_008884.2 |
| Tnfrsf14 | 1.49 | 0.0584968 | 0.73 | NM_178931.2 |
| Tnfrsf1b | 1.49 | 0.0628318 | 0.76 | NM_011610.3 |
| G6pdx | 1.48 | 0.0011136 | 0.09 | NM_008062.2 |
| Lilra6 | 1.47 | 0.0128931 | 0.31 | NM_011090.2 |
| Csf2 | 1.45 | 0.1149513 | 1 | NM_009969.4 |
| Il4ra | 1.45 | 0.1258069 | 1 | NM_001008700.3 |
| Prkcd | 1.45 | 0.0141691 | 0.33 | NM_011103.2 |
| Ifngr1 | 1.44 | 0.0219081 | 0.41 | NM_010511.2 |
| Cdkn1a | 1.42 | 0.0350353 | 0.52 | NM_007669.4 |
| Ltb4r1 | 1.39 | 0.0315028 | 0.49 | NM_008519.2 |
| Gapdh | 1.38 | 0.0013488 | 0.1 | NM_001001303.1 |
| Selplg | 1.37 | 0.1337783 | 1 | NM_009151.3 |
| Slamf1 | 1.37 | 0.0588486 | 0.73 | NM_013730.4 |
| Prim1 | 1.36 | 0.0018498 | 0.13 | NM_008921.2 |
| Abcb1a | 1.34 | 0.0026209 | 0.15 | NM_011076.1 |
| Ddx58 | 1.34 | 0.0179144 | 0.36 | NM_172689.3 |
| Tnfsf14 | 1.34 | 0.0005103 | 0.07 | NM_019418.2 |
| Cd4 | 1.32 | 0.034996 | 0.52 | NM_013488.2 |
| Tnfrsf11a | 1.3 | 0.0165236 | 0.35 | NM_009399.3 |
| Syk | 1.28 | 0.1760462 | 1 | NM_011518.2 |
| Tyk2 | 1.28 | 0.0875971 | 0.98 | NM_018793.2 |
| Entpd1 | 1.25 | 0.1325421 | 1 | NM_009848.3 |
| Ebi3 | 1.24 | 0.0416876 | 0.58 | NM_015766.2 |
| Mapkapk2 | 1.23 | 6.074E-05 | 0.04 | NM_008551.1 |
| C1s | 1.22 | 0.1437591 | 1 | NM_144938.2 |
| H2-Q10 | 1.2 | 0.0546127 | 0.7 | NM_010391.4 |
| Nt5e | 1.17 | 0.1830825 | 1 | NM_011851.3 |
| Ifngr2 | 1.15 | 0.0795591 | 0.91 | NM_008338.3 |
| Nfkb2 | 1.15 | 0.0035614 | 0.18 | NM_019408.2 |
| Tbk1 | 1.15 | 0.0083205 | 0.25 | NM_019786.4 |
| Cd34 | 1.14 | 0.1326526 | 1 | NM_001111059.1 |
| Fn1 | 1.14 | 0.1996376 | 1 | NM_010233.1 |
| Myd88 | 1.14 | 0.0290451 | 0.47 | NM_010851.2 |
| Folr4 | 1.13 | 0.1493931 | 1 | NM_022888.2 |
| Cd226 | 1.12 | 0.1192841 | 1 | NM_001039149.1 |
| Nfil3 | 1.12 | 0.0886159 | 0.98 | NM_017373.3 |
| Nos2 | 1.12 | 0.1209268 | 1 | NM_010927.3 |
| Tlr9 | 1.08 | 0.0147689 | 0.33 | NM_031178.2 |
| Ikzf1 | 1.07 | 0.0765916 | 0.89 | NM_001025597.1 |
| Tlr4 | 1.07 | 0.0254111 | 0.45 | NM_021297.2 |
| Gpr183 | 1.06 | 0.0107328 | 0.28 | NM_183031.2 |
| Ptger4 | 1.03 | 0.0039995 | 0.19 | NM_008965.1 |
| Ptpn2 | 1.03 | 0.0051876 | 0.2 | NM_001127177.1 |
| Nfkbia | 1.01 | 0.0386803 | 0.56 | NM_010907.2 |
| Gusb | 1.01 | 0.0598726 | 0.74 | NM_010368.1 |
| C1ra | 0.98 | 0.0547535 | 0.7 | NM_023143.3 |
| Ccr9 | 0.98 | 0.0708204 | 0.84 | NM_009913.6 |
| Litaf | 0.98 | 0.0043998 | 0.2 | NM_019980.1 |
| Tmem173 | 0.97 | 0.0012212 | 0.1 | NM_028261.1 |
| Jak2 | 0.96 | 0.0278494 | 0.47 | NM_001048177.1 |
| Jak3 | 0.95 | 0.1261171 | 1 | NM_010589.5 |
| Tyrobp | 0.95 | 0.0434109 | 0.6 | NM_011662.2 |
| Cebpb | 0.94 | 0.0019347 | 0.13 | NM_009883.3 |
| Relb | 0.93 | 0.0071823 | 0.24 | NM_009046.2 |
| Cd44 | 0.91 | 0.002987 | 0.17 | NM_009851.2 |
| Mx1 | 0.91 | 0.0863738 | 0.97 | NM_010846.1 |
| Camp | 0.87 | 0.5569863 | 1 | NM_009921.2 |
| Cd163 | 0.86 | 0.0696456 | 0.83 | NM_053094.2 |
| Ets1 | 0.85 | 0.216888 | 1 | NM_001038642.1 |
| Ceacam1 | 0.84 | 0.6063904 | 1 | NM_001039185.1 |
| Gm10499 | 0.84 | 0.1185757 | 1 | XM_003086920.1 |
| H2-Eb1 | 0.83 | 0.256508 | 1 | NM_010382.2 |
| Il1rl1 | 0.83 | 0.0139817 | 0.33 | NM_001025602.2 |
| Bax | 0.81 | 0.0380412 | 0.55 | NM_007527.3 |
| Ccl19 | 0.8 | 0.1681242 | 1 | NM_011888.2 |
| Ifnar2 | 0.8 | 0.0129747 | 0.31 | NM_001110498.1 |
| Irf4 | 0.79 | 0.0290615 | 0.47 | NM_013674.1 |
| Ifnb1 | 0.78 | 0.3232229 | 1 | NM_010510.1 |
| Fas | 0.76 | 0.0387032 | 0.56 | NM_007987.2 |
| Nfatc1 | 0.76 | 0.2467893 | 1 | NM_016791.4 |
| Jak1 | 0.75 | 0.1101651 | 1 | NM_146145.2 |
| Cd96 | 0.74 | 0.0616241 | 0.76 | NM_032465.2 |
| Psmb7 | 0.74 | 0.0282039 | 0.47 | NM_011187.1 |
| Ifitm1 | 0.73 | 0.0776491 | 0.9 | NM_001112715.1 |
| Ifi35 | 0.72 | 0.070156 | 0.83 | NM_027320.4 |
| Serping1 | 0.72 | 0.0307103 | 0.48 | NM_009776.3 |
| Cd40 | 0.71 | 0.2711878 | 1 | NM_011611.2 |
| Abcf1 | 0.7 | 0.0222998 | 0.41 | NM_013854.1 |
| Mif | 0.7 | 0.0257494 | 0.45 | NM_010798.2 |
| Cxcl12 | 0.69 | 0.3574634 | 1 | NM_021704.3 |
| Ilf3 | 0.64 | 0.0328009 | 0.5 | NM_010561.2 |
| C1qbp | 0.63 | 0.02251 | 0.41 | NM_007573.2 |
| Il3 | 0.63 | 0.3284916 | 1 | NM_010556.4 |
| Psmc2 | 0.63 | 0.0287682 | 0.47 | NM_011188.3 |
| Aicda | 0.61 | 0.4607817 | 1 | NM_009645.2 |
| Pecam1 | 0.61 | 0.4724157 | 1 | NM_008816.2 |
| Stat5a | 0.61 | 0.0276807 | 0.47 | NM_011488.2 |
| Cmklr1 | 0.6 | 0.0598588 | 0.74 | NM_008153.3 |
| Nfatc3 | 0.58 | 0.0196345 | 0.38 | NM_010901.2 |
| Traf6 | 0.58 | 0.1887481 | 1 | NM_009424.2 |
| Kir3dl2 | 0.57 | 0.4980274 | 1 | NM_177748.2 |
| Stat3 | 0.56 | 0.0907087 | 1 | NM_213659.2 |
| H2-Ea-ps | 0.55 | 0.9218131 | 1 | NM_010381.2 |
| Mapk11 | 0.54 | 0.4443533 | 1 | NM_011161.5 |
| Pdcd1lg2 | 0.54 | 0.4988704 | 1 | NM_021396.2 |
| C4a | 0.52 | 0.062277 | 0.76 | NM_011413.2 |
| Ifnar1 | 0.52 | 0.1505978 | 1 | NM_010508.1 |
| Tnfsf11 | 0.52 | 0.1710879 | 1 | NM_011613.3 |
| Icam1 | 0.51 | 0.2787167 | 1 | NM_010493.2 |
| Klra6 | 0.47 | 0.0769938 | 0.9 | NM_008464.2 |
| Klrb1 | 0.47 | 0.4303455 | 1 | NM_001099918.1 |
| Mapk14 | 0.47 | 0.0982372 | 1 | NM_011951.2 |
| Cdh5 | 0.45 | 0.656084 | 1 | NM_009868.3 |
| Il10rb | 0.43 | 0.16048 | 1 | NM_008349.5 |
| C3 | 0.4 | 0.2229978 | 1 | NM_009778.2 |
| Psmb5 | 0.39 | 0.136642 | 1 | NM_011186.1 |
| Cd82 | 0.38 | 0.0758712 | 0.89 | NM_001271430.1 |
| Cxcr4 | 0.37 | 0.0287564 | 0.47 | NM_009911.3 |
| Tlr3 | 0.37 | 0.2118409 | 1 | NM_126166.2 |
| Eef1g | 0.37 | 0.0532734 | 0.69 | NM_026007.4 |
| Tcf7 | 0.36 | 0.6290699 | 1 | NM_009331.3 |
| Itga6 | 0.35 | 0.5104104 | 1 | NM_008397.3 |
| Traf1 | 0.35 | 0.2032527 | 1 | NM_009421.3 |
| Nfkb1 | 0.34 | 0.269219 | 1 | NM_008689.2 |
| Klrc2 | 0.33 | 0.3644498 | 1 | NM_001098669.1 |
| Mbl2 | 0.32 | 0.3239537 | 1 | NM_010776.1 |
| Polr2a | 0.32 | 0.2014049 | 1 | NM_009089.2 |
| Lcp2 | 0.31 | 0.875152 | 1 | NM_010696.3 |
| Rpl19 | 0.31 | 0.0031428 | 0.17 | NM_009078.2 |
| Il17f | 0.3 | 0.3785319 | 1 | NM_145856.2 |
| Itgax | 0.29 | 0.7100769 | 1 | NM_021334.2 |
| Atg16l1 | 0.28 | 0.1554484 | 1 | NM_029846.3 |
| Il4 | 0.27 | 0.7662538 | 1 | NM_021283.1 |
| Mapk1 | 0.27 | 0.0862489 | 0.97 | NM_011949.3 |
| Il23r | 0.26 | 0.6376026 | 1 | NM_144548.1 |
| Lif | 0.26 | 0.5907744 | 1 | NM_008501.2 |
| Irak2 | 0.25 | 0.0739073 | 0.87 | NM_001113553.1 |
| Kir3dl1 | 0.25 | 0.6830236 | 1 | NM_177749.3 |
| Cd40lg | 0.24 | 0.6724737 | 1 | NM_011616.2 |
| Tgfbr2 | 0.22 | 0.3970318 | 1 | NM_009371.2 |
| Irak1 | 0.21 | 0.3652517 | 1 | NM_008363.2 |
| Il2 | 0.2 | 0.8700941 | 1 | NM_008366.2 |
| Tgfbr1 | 0.2 | 0.606505 | 1 | NM_009370.2 |
| Ltbr | 0.18 | 0.6172841 | 1 | NM_010736.3 |
| Ppia | 0.18 | 0.0789684 | 0.91 | NM_008907.1 |
| Il15 | 0.17 | 0.776235 | 1 | NM_008357.2 |
| Il22 | 0.17 | 0.8452495 | 1 | NM_016971.1 |
| Rela | 0.16 | 0.6104717 | 1 | NM_009045.4 |
| Cish | 0.15 | 0.7972467 | 1 | NM_009895.3 |
| Traf2 | 0.14 | 0.4981577 | 1 | NM_009422.2 |
| Phlpp1 | 0.11 | 0.7785558 | 1 | NM_133821.3 |
| Pdcd2 | 0.09 | 0.6722952 | 1 | NM_008799.2 |
| Tnfsf12 | 0.09 | 0.8174298 | 1 | NM_011614.3 |
| Xbp1 | 0.09 | 0.6560177 | 1 | NM_013842.2 |
| Il1rl2 | 0.08 | 0.7080678 | 1 | NM_133193.3 |
| Atm | 0.05 | 0.8791977 | 1 | NM_007499.1 |
| Klra8 | 0.04 | 0.9404208 | 1 | NM_010650.3 |
| Ikbkb | 0.03 | 0.949235 | 1 | NM_010546.2 |
| C4bp | 0.02 | 0.9805694 | 1 | NM_007576.3 |
| Klra5 | 0.01 | 0.993182 | 1 | NM_008463.2 |
| Traf3 | 0 | 0.9931365 | 1 | NM_001048206.1 |
| Itgb1 | -0.01 | 0.9673955 | 1 | NM_010578.1 |
| Gpi1 | -0.02 | 0.8941486 | 1 | NM_008155.3 |
| Map4k4 | -0.02 | 0.9678251 | 1 | NM_008696.2 |
| Stat6 | -0.05 | 0.7848321 | 1 | NM_009284.2 |
| Psmd7 | -0.06 | 0.6910985 | 1 | NM_010817.2 |
| Tirap | -0.06 | 0.8186016 | 1 | NM_001177847.1 |
| Gp1bb | -0.07 | 0.9336411 | 1 | NM_010327.2 |
| Abl1 | -0.08 | 0.8026062 | 1 | NM_009594.3 |
| Icam2 | -0.11 | 0.8768861 | 1 | NM_010494.1 |
| Il12a | -0.11 | 0.8259358 | 1 | NM_008351.1 |
| Lta | -0.12 | 0.8662897 | 1 | NM_010735.1 |
| Cd244 | -0.14 | 0.8007817 | 1 | NM_018729.2 |
| Nox4 | -0.15 | 0.8075365 | 1 | NM_015760.4 |
| Tollip | -0.15 | 0.5924719 | 1 | NM_023764.3 |
| Traf5 | -0.15 | 0.8028883 | 1 | NM_011633.1 |
| Cx3cr1 | -0.16 | 0.6603217 | 1 | NM_009987.3 |
| Il7r | -0.16 | 0.7143115 | 1 | NM_008372.3 |
| Klra4 | -0.16 | 0.8808534 | 1 | NM_010649.3 |
| Notch1 | -0.16 | 0.545036 | 1 | NM_008714.2 |
| Nox3 | -0.16 | 0.6611003 | 1 | NM_198958.2 |
| Pla2g2a | -0.16 | 0.8085803 | 1 | NM_001082531.1 |
| Tnfsf10 | -0.16 | 0.8598753 | 1 | NM_009425.2 |
| Oaz1 | -0.16 | 0.0607702 | 0.75 | NM_008753.4 |
| Il17a | -0.17 | 0.7554272 | 1 | NM_010552.3 |
| Cd164 | -0.18 | 0.4468157 | 1 | NM_016898.2 |
| Chuk | -0.19 | 0.1356865 | 1 | NM_001162410.1 |
| Klra21 | -0.2 | 0.7652028 | 1 | NM_053151.1 |
| Tnfsf13b | -0.21 | 0.5526675 | 1 | NM_033622.1 |
| Notch2 | -0.22 | 0.2298004 | 1 | NM_010928.1 |
| Hamp | -0.24 | 0.8765279 | 1 | NM_032541.1 |
| Sigirr | -0.24 | 0.2212164 | 1 | NM_023059.3 |
| Fcgrt | -0.25 | 0.4658868 | 1 | NM_010189.3 |
| Pla2g2e | -0.25 | 0.5816023 | 1 | NM_012044.2 |
| Tnfsf8 | -0.25 | 0.3619055 | 1 | NM_009403.2 |
| Klrc3 | -0.26 | 0.7910786 | 1 | NM_021378.1 |
| Irf3 | -0.27 | 0.340211 | 1 | NM_016849.3 |
| Sdha | -0.27 | 0.1612008 | 1 | NM_023281.1 |
| Ccr10 | -0.28 | 0.6922935 | 1 | NM_007721.4 |
| Hfe | -0.28 | 0.4698 | 1 | NM_010424.4 |
| Il18r1 | -0.28 | 0.2308332 | 1 | NM_001161842.1 |
| Pdgfrb | -0.28 | 0.6592347 | 1 | NM_008809.1 |
| Stat5b | -0.29 | 0.416318 | 1 | NM_011489.3 |
| Il19 | -0.3 | 0.5730308 | 1 | NM_001009940.1 |
| Il13ra1 | -0.31 | 0.2169045 | 1 | NM_133990.4 |
| Klra7 | -0.32 | 0.6564035 | 1 | NM_001110323.1 |
| Ccl11 | -0.33 | 0.3055863 | 1 | NM_011330.3 |
| Tubb5 | -0.34 | 0.2712629 | 1 | NM_011655.4 |
| App | -0.36 | 0.4243048 | 1 | NM_007471.2 |
| Il12b | -0.36 | 0.6033105 | 1 | NM_008352.1 |
| Ube2l3 | -0.36 | 0.0586885 | 0.73 | NM_009456.2 |
| Ccl24 | -0.37 | 0.5654889 | 1 | NM_019577.4 |
| Ifna1 | -0.37 | 0.6686585 | 1 | NM_010502.2 |
| Npc1 | -0.38 | 0.1071438 | 1 | NM_008720.2 |
| Polr1b | -0.38 | 0.3108788 | 1 | NM_009086.2 |
| Cd59b | -0.39 | 0.4864941 | 1 | NM_181858.1 |
| Rag2 | -0.4 | 0.2844053 | 1 | NM_009020.3 |
| Src | -0.4 | 0.2567385 | 1 | NM_001025395.2 |
| C8a | -0.42 | 0.1921928 | 1 | NM_146148.1 |
| Cd28 | -0.42 | 0.4979309 | 1 | NM_007642.4 |
| Il5 | -0.42 | 0.5638523 | 1 | NM_010558.1 |
| Il23a | -0.44 | 0.3594158 | 1 | NM_031252.1 |
| Il25 | -0.44 | 0.6714154 | 1 | NM_080729.2 |
| Tcf4 | -0.44 | 0.2302389 | 1 | NM_013685.1 |
| Igf2r | -0.45 | 0.1011049 | 1 | NM_010515.1 |
| Il28a | -0.45 | 0.2189115 | 1 | NM_001024673.2 |
| Tnfrsf13b | -0.45 | 0.0844676 | 0.95 | NM_021349.1 |
| Gata3 | -0.47 | 0.3853846 | 1 | NM_008091.3 |
| Tbp | -0.48 | 0.1278172 | 1 | NM_013684.3 |
| C8g | -0.5 | 0.1854442 | 1 | NM_027062.1 |
| Phlpp2 | -0.51 | 0.321867 | 1 | NM_001122594.2 |
| Foxp3 | -0.53 | 0.2804676 | 1 | NM_054039.1 |
| Ptgs2 | -0.53 | 0.3771349 | 1 | NM_011198.3 |
| Rae1 | -0.53 | 0.0799647 | 0.91 | NM_175112.5 |
| Cfi | -0.54 | 0.1215879 | 1 | NM_007686.2 |
| Pdgfb | -0.54 | 0.4641382 | 1 | NM_011057.3 |
| Tnfrsf17 | -0.54 | 0.2776507 | 1 | NM_011608.1 |
| Il1rap | -0.55 | 0.1387316 | 1 | NM_134103.2 |
| Xcr1 | -0.55 | 0.5734494 | 1 | NM_011798.4 |
| Cradd | -0.56 | 0.0073493 | 0.24 | NM_009950.2 |
| Map4k2 | -0.6 | 0.1641548 | 1 | NM_009006.2 |
| Ikbkap | -0.61 | 0.1025682 | 1 | NM_026079.3 |
| Ccr7 | -0.62 | 0.402182 | 1 | NM_007719.2 |
| Mbp | -0.62 | 0.2116894 | 1 | NM_010777.3 |
| Frmpd4 | -0.65 | 0.3563273 | 1 | NM_001033330.2 |
| Tal1 | -0.65 | 0.4743554 | 1 | NM_011527.2 |
| Fcer1a | -0.66 | 0.1838169 | 1 | NM_010184.1 |
| Icosl | -0.66 | 0.0763872 | 0.89 | NM_015790.3 |
| Tnfaip6 | -0.67 | 0.1361627 | 1 | NM_009398.2 |
| Cd97 | -0.68 | 0.2937244 | 1 | NM_011925.1 |
| Adal | -0.69 | 0.109952 | 1 | NM_029475.1 |
| Smad3 | -0.69 | 0.0507648 | 0.67 | NM_016769.3 |
| Defb1 | -0.7 | 0.1499153 | 1 | NM_007843.3 |
| Fcamr | -0.7 | 0.266069 | 1 | NM_001170632.1 |
| Ccl22 | -0.71 | 0.1252857 | 1 | NM_009137.2 |
| Ifna2 | -0.71 | 0.3942399 | 1 | NM_010503.2 |
| Fadd | -0.72 | 0.0564311 | 0.71 | NM_010175.5 |
| Cd109 | -0.73 | 0.3575026 | 1 | NM_153098.3 |
| Cul9 | -0.73 | 0.0293611 | 0.47 | NM_001081335.2 |
| Rag1 | -0.73 | 0.0947777 | 1 | NM_009019.2 |
| Itln1 | -0.74 | 0.5533294 | 1 | NM_010584.3 |
| Ccr3 | -0.75 | 0.3466477 | 1 | NM_009914.4 |
| Defb14 | -0.75 | 0.3442068 | 1 | NM_183026.2 |
| Ikbkg | -0.75 | 0.0111251 | 0.29 | NM_178590.2 |
| Il17b | -0.76 | 0.0432384 | 0.6 | NM_019508.1 |
| Ccl26 | -0.77 | 0.5320338 | 1 | NM_001013412.2 |
| Itga2b | -0.78 | 0.3638153 | 1 | NM_010575.2 |
| Ski | -0.79 | 0.1099241 | 1 | NM_011385.2 |
| Lilra5 | -0.8 | 0.2755213 | 1 | NM_001081239.2 |
| Zeb1 | -0.81 | 0.1955178 | 1 | NM_011546.2 |
| Klra1 | -0.82 | 0.2031 | 1 | NM_016659.3 |
| Psmb11 | -0.82 | 0.3398245 | 1 | NM_175204.4 |
| Rorc | -0.82 | 0.0337173 | 0.51 | NM_011281.2 |
| Cr2 | -0.83 | 0.5158125 | 1 | NM_007758.2 |
| Cd99 | -0.87 | 0.0213747 | 0.4 | NM_025584.2 |
| Ikzf2 | -0.87 | 0.0576861 | 0.73 | NM_011770.4 |
| Tgfb2 | -0.88 | 0.1713884 | 1 | NM_009367.1 |
| Tslp | -0.88 | 0.0538282 | 0.69 | NM_021367.1 |
| Hprt | -0.89 | 0.0110484 | 0.29 | NM_013556.2 |
| Bcl6 | -0.9 | 0.0165516 | 0.35 | NM_009744.3 |
| Ccl25 | -0.91 | 0.0100224 | 0.27 | NM_009138.3 |
| Ikzf4 | -0.92 | 0.1631445 | 1 | NM_011772.2 |
| Tnfrsf13c | -0.92 | 0.2749116 | 1 | NM_028075.2 |
| Ptk2 | -0.93 | 0.1405942 | 1 | NM_007982.2 |
| Runx1 | -0.94 | 0.0112339 | 0.29 | NM_001111021.1 |
| Nox1 | -0.95 | 0.3487175 | 1 | NM_172203.1 |
| Trp53 | -0.96 | 0.0194289 | 0.38 | NM_011640.1 |
| Btla | -0.97 | 0.1972172 | 1 | NM_177584.3 |
| Dpp4 | -0.98 | 0.1351868 | 1 | NM_001159543.1 |
| Bcap31 | -0.99 | 6.868E-05 | 0.04 | NM_012060.4 |
| C8b | -1.01 | 0.2312607 | 1 | NM_133882.2 |
| Cd81 | -1.01 | 0.0071775 | 0.24 | NM_133655.2 |
| Ms4a1 | -1.01 | 0.5258097 | 1 | NM_007641.5 |
| Aire | -1.08 | 0.2888354 | 1 | NM_009646.1 |
| Cd46 | -1.09 | 0.1821291 | 1 | NM_010778.3 |
| Vtn | -1.11 | 0.1366206 | 1 | NM_011707.2 |
| Casp2 | -1.14 | 0.0524525 | 0.69 | NM_007610.1 |
| Zbtb7b | -1.14 | 0.0179511 | 0.36 | NM_009565.4 |
| Icam5 | -1.16 | 0.124993 | 1 | NM_008319.2 |
| Il9 | -1.16 | 0.3629811 | 1 | NM_008373.1 |
| Pparg | -1.18 | 0.0435893 | 0.6 | NM_011146.1 |
| Icam4 | -1.2 | 0.0681325 | 0.82 | NM_023892.2 |
| Ccr4 | -1.25 | 0.1508965 | 1 | NM_009916.2 |
| Il6st | -1.25 | 0.0010461 | 0.09 | NM_010560.2 |
| Masp1 | -1.26 | 0.0458523 | 0.62 | NM_008555.2 |
| Tnfsf15 | -1.26 | 0.0531211 | 0.69 | NM_177371.3 |
| Cx3cl1 | -1.27 | 0.0271936 | 0.47 | NM_009142.3 |
| C9 | -1.28 | 0.1480568 | 1 | NM_013485.1 |
| Cd79a | -1.31 | 0.3548082 | 1 | NM_007655.3 |
| Btnl1 | -1.32 | 0.1541574 | 1 | NM_001111094.1 |
| Il20 | -1.36 | 0.1950836 | 1 | NM_021380.1 |
| Il6ra | -1.36 | 0.0656785 | 0.79 | NM_010559.2 |
| Tnfsf18 | -1.36 | 0.2368281 | 1 | NM_183391.3 |
| Ppbp | -1.37 | 0.2064549 | 1 | NM_023785.2 |
| Cd19 | -1.38 | 0.3725296 | 1 | NM_009844.2 |
| Ctnnb1 | -1.38 | 0.0076081 | 0.24 | NM_007614.2 |
| Ltf | -1.38 | 0.1166267 | 1 | NM_008522.3 |
| Abcb10 | -1.39 | 0.0541188 | 0.69 | NM_019552.2 |
| Bcl2 | -1.39 | 0.0088652 | 0.25 | NM_009741.3 |
| Tfrc | -1.4 | 0.0039389 | 0.19 | NM_011638.3 |
| Ccbp2 | -1.42 | 0.0187814 | 0.37 | NM_021609.3 |
| Pigr | -1.42 | 0.0016694 | 0.12 | NM_011082.3 |
| Cxcl15 | -1.44 | 0.026894 | 0.47 | NM_011339.2 |
| Cd79b | -1.45 | 0.1154602 | 1 | NM_008339.2 |
| Cxcr1 | -1.45 | 0.0885159 | 0.98 | NM_178241.4 |
| Gpr44 | -1.49 | 0.0348763 | 0.52 | NM_009962.2 |
| Mr1 | -1.49 | 0.0002259 | 0.06 | NM_008209.4 |
| Cfh | -1.54 | 0.0191314 | 0.38 | NM_009888.3 |
| Il17rb | -1.54 | 0.0120666 | 0.3 | NM_019583.3 |
| Traf4 | -1.55 | 0.0044486 | 0.2 | NM_009423.4 |
| Cd3eap | -1.57 | 0.0180463 | 0.36 | NM_145822.2 |
| Btnl2 | -1.59 | 0.01935 | 0.38 | NM_079835.2 |
| Il22ra2 | -1.6 | 0.0891479 | 0.99 | NM_178258.5 |
| Cd83 | -1.63 | 0.0403955 | 0.58 | NM_009856.2 |
| Ltb4r2 | -1.68 | 0.0081189 | 0.25 | NM_020490.2 |
| Il33 | -1.72 | 0.0561591 | 0.71 | NM_133775.1 |
| Tlr5 | -1.72 | 0.005023 | 0.2 | NM_016928.2 |
| Cd9 | -1.74 | 0.0145131 | 0.33 | NM_007657.3 |
| Cxcr5 | -1.75 | 0.0798983 | 0.91 | NM_007551.2 |
| Smad5 | -1.77 | 0.0153849 | 0.34 | NM_008541.2 |
| H2-Ob | -1.81 | 0.0587444 | 0.73 | NM_010389.3 |
| Il1r1 | -1.83 | 0.0112586 | 0.29 | NM_001123382.1 |
| Il17re | -1.84 | 0.0054066 | 0.2 | NM_001034029.1 |
| C7 | -1.92 | 0.0365021 | 0.54 | XM_356827.6 |
| Hlx | -1.92 | 0.0125983 | 0.31 | NM_008250.2 |
| Clu | -1.96 | 0.0154202 | 0.34 | NM_013492.2 |
| Masp2 | -2 | 0.1810228 | 1 | NM_010767.3 |
| Cd36 | -2.08 | 0.0090559 | 0.25 | NM_007643.3 |
| Ahr | -2.1 | 0.0019728 | 0.13 | NM_013464.4 |
| Tgfb3 | -2.11 | 0.0034017 | 0.18 | NM_009368.2 |
| Blnk | -2.17 | 0.0002266 | 0.06 | NM_008528.4 |
| Ccr6 | -2.17 | 0.0363171 | 0.54 | NM_001190333.1 |
| Ccl20 | -2.2 | 0.0147384 | 0.33 | NM_016960.1 |
| Mme | -2.39 | 0.0170835 | 0.36 | NM_008604.3 |
| Muc1 | -2.39 | 0.0006453 | 0.08 | NM_013605.1 |
| Il7 | -2.41 | 0.0045208 | 0.2 | NM_008371.2 |
| Cd22 | -2.42 | 0.0529579 | 0.69 | NM_001043317.2 |
| Ncam1 | -2.42 | 0.0106758 | 0.28 | NM_001113204.1 |
| Alas1 | -2.54 | 0.0178249 | 0.36 | NM_020559.2 |
| Cd24a | -2.57 | 0.0089773 | 0.25 | NM_009846.2 |
| Ccrl1 | -2.64 | 0.0362486 | 0.54 | NM_145700.2 |
| Cd55 | -2.68 | 0.0005849 | 0.07 | NM_010016.2 |
| Il13 | -2.68 | 0.0851822 | 0.96 | NM_008355.2 |
| C6 | -2.7 | 0.0078301 | 0.24 | NM_016704.2 |
| Kit | -2.73 | 0.0321858 | 0.5 | NM_001122733.1 |
| Pax5 | -3.32 | 0.0489636 | 0.65 | NM_008782.2 |
| Cfd | -3.36 | 0.0385243 | 0.56 | NM_013459.1 |
| Cd209g | -3.42 | 0.0013624 | 0.1 | NM_027343.3 |
| Il11ra1 | -3.92 | 0.0023764 | 0.15 | NM_010549.3 |
| Hc | -4.27 | 0.0050029 | 0.2 | NM_010406.1 |
| Ccr8 | -4.47 | 0.0006951 | 0.08 | NM_007720.2 |
